# Supplementary material for: A modular biomimetic strategy for the synthesis of macrolide P-glycoprotein inhibitors via Rh-catalyzed C-H activation
Source: Nat Commun. 2020 May 1;11:2151. doi: 10.1038/s41467-020-16084-0 (PMC7195407; doi:10.1038/s41467-020-16084-0)
Supplement: Supplementary file 1 — Supplementary Information [file 41467_2020_16084_MOESM1_ESM.pdf]

Supplementary Information

A modular biomimetic strategy for the synthesis of macrolide P-glycoprotein inhibitors via Rh-catalyzed C-H activation

Lu Chen[1,2], Haitian Quan[1,2], Zhongliang Xu[1,2], Hao Wang[1,2], Yuanzhi Xia[3], Liguang Lou*[1,2] and Weibo Yang*[1,2,4]

[1] Chinese Academy of Sciences Key Laboratory of Receptor Research, Shanghai Institute of Materia Medica (SIMM), Chinese Academy of Sciences, Shanghai, China

[2]University of Chinese Academy of Sciences, Beijing 100049, China

[3]College of Chemistry and Materials Engineering, Wenzhou University, Wenzhou 325035, China

[4]Key Laboratory for Functional Material, Educational Department of Liaoning Province, University of Science and Technology Liaoning, Anshan 114051, China

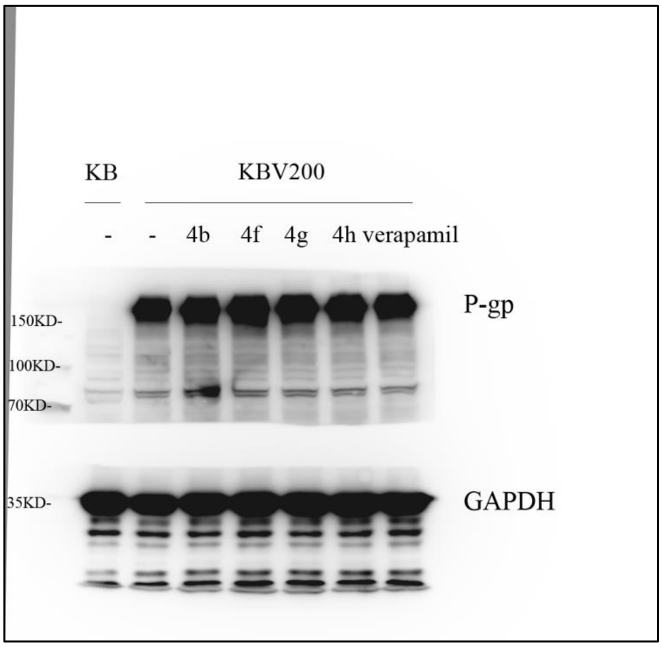

Supplementary Figure 1 4b, 4f, 4g and 4h did not affect P-gp expression in KBV200 cells. KB or KBV200 cells were treated with 10 μM **4b, 4f, 4g and 4h** or verapamil for 24 hours, and P-gp and GAPDH were analyzed by Western blotting.

Results from the first time

A

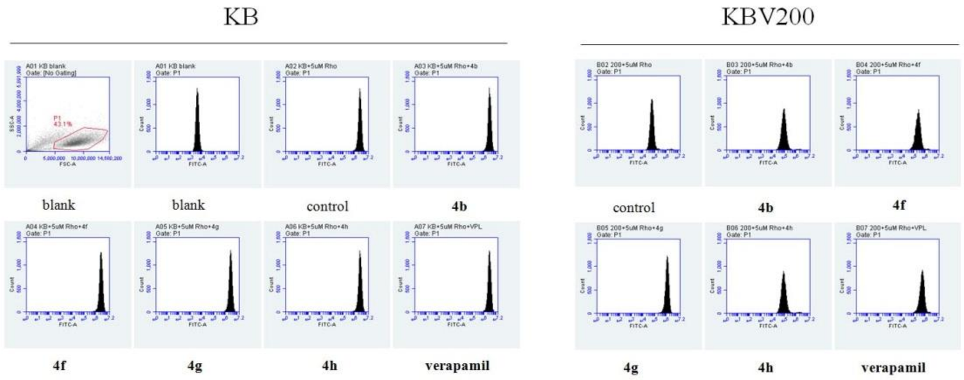

B

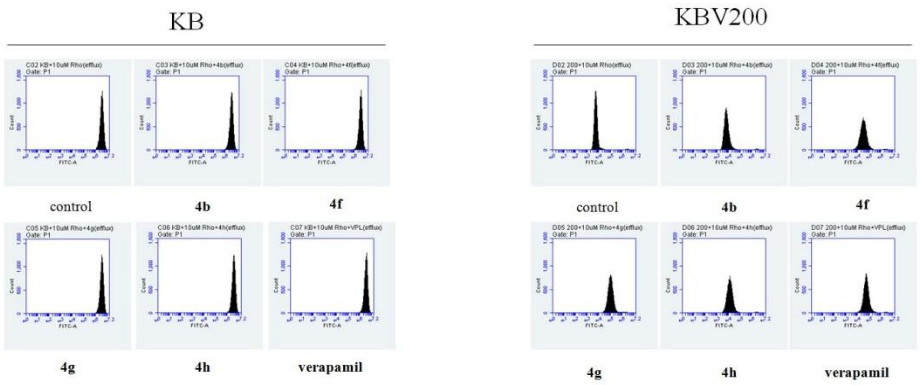

Results from the second time

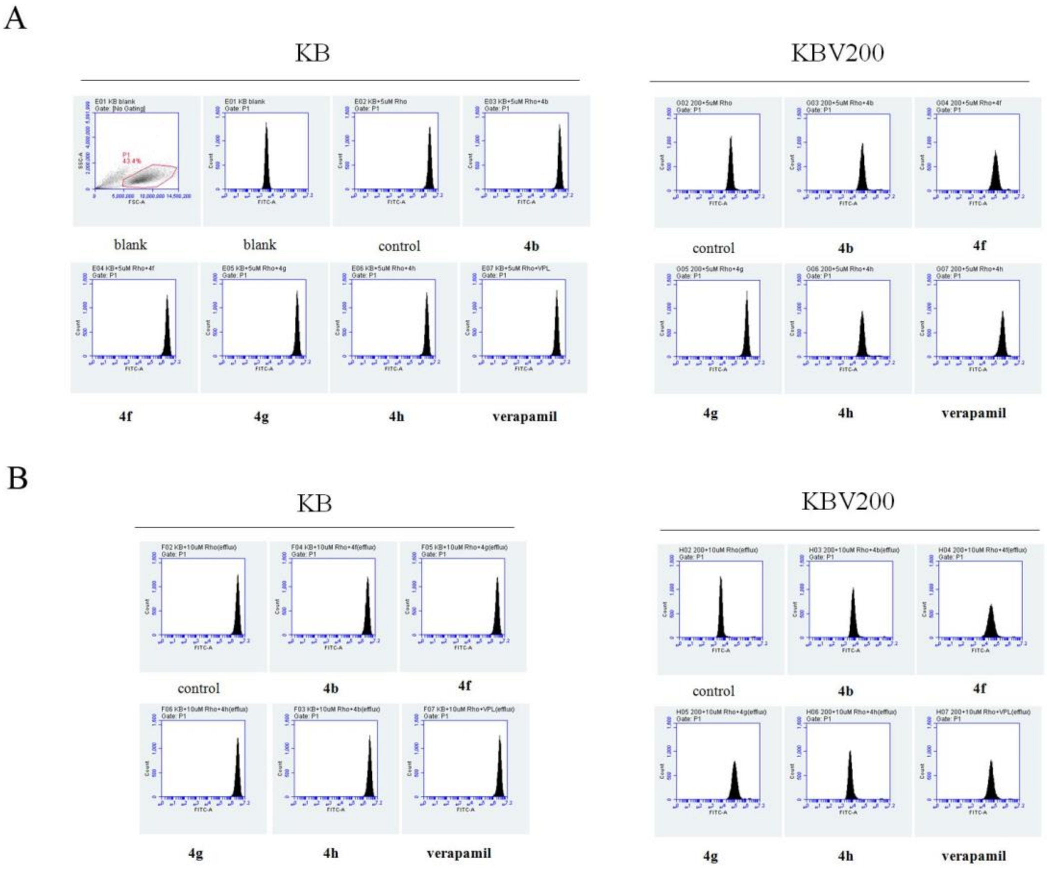

Supplementary Figure 2 4b, 4f, 4g and 4h increased Rho-123 uptake and inhibited Rho-123 efflux in KBV200 cells. (A) KB or KBV200 cells were incubated with 5 μM Rho-123, in the presence or absence of 10 μM compounds at 37 ºC for 1 hour, and Rho-123 accumulation was determined by flow cytometry; (B) KB or KBV200 cells were incubated with 10 μM Rho-123 for 1 hour, and then washed, incubated in Rho-123-free medium, with or without 10 μM compounds for additional 1 hour, and efflux of Rho-123 were determined by flow cytometry. Results shown are from two independent experiments.

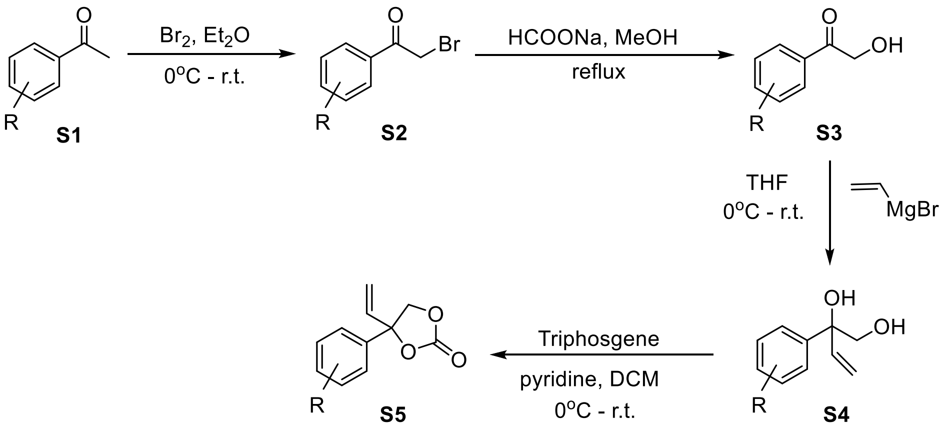

Supplementary Figure 3 Typical procedure for the preparation of vinylethylene carbonates. Bromine (5 mmol, 1.0 equiv.) was added to a solution of the respective ketone (5 mmol, 1.0 equiv.) in Et_2O (6 mL) at 0 °C. And then 15 minutes later, the solution was stirred for 6-12 h (until the color of the solution changed from red to light-yellow) at r.t.. Upon completion, the reaction was quenched with ice water (6 mL), and extracted with Et_2O (3 × 5 mL). The combined organic phases were washed with saturated aqueous $NaHCO_3$ solution (10 mL), brine (10 mL), and dried over Na_2SO_4. After being filtered and concentrated, the residue was used for the next step without further purification (the crude **S2**). To a solution of resultant crude **S2** (1.0 equiv.) in methanol (3 mL/mmol) was added sodium formate (3.0 equiv.) at r.t.. Upon completion, the reaction mixture was filtered. The filtrate was concentrated under reduced pressure. The residue was dissolved with ethyl acetate, washed with water and brine solution, dried over Na_2SO_4 and evaporated. The crude material was purified by column chromatography to get **S3**. To a solution of the **S3** (1.0 equiv.) in THF (4 mL/mmol) was added vinylmagnesium bromide (1.0 M in THF, 2.5 equiv.) at 0 °C. The reaction was stirred under an N_2 atmosphere at room temperature for 2-3 h. The reaction mixture was then quenched with saturated aqueous NH_4Cl, and extracted with ethyl acetate. The combined organic layers were dried over anhydrous Na_2SO_4, filtered and concentrated. Then the crude product was purified by FCC to get the **S4**. To a solution of **S4** in DCM (1.5 mL/mmol) added pyridine (4 equiv.) and triphosgene (0.5 equiv.) at 0 °C. The reaction was stirred under an N_2 atmosphere at room temperature for 1-2 h. The reaction mixture was then quenched with saturated aqueous NH_4Cl, washed with H_2O, and

extracted with DCM. The combined organic layers were dried over anhydrous Na_2SO_4, filtered and concentrated. The residue was purified by flash chromatography on silica to afford the corresponding carbonate (**S5**).[1,2]

Supplementary Methods

General comments:

NMR spectra were recorded at room temperature on the following spectrometers: Bruker Avance III 400 Spectrometer (400 MHz), Bruker Avance III 500 (Cryo) Spectrometer (500 MHz) and Bruker Avance III 600 Spectrometer (600 MHz) . Chemical shifts are given in ppm and coupling constants in Hz. ^1H spectra were calibrated in relation to the reference measurement of TMS (0.00 ppm). ^{13}C spectra were calibrated in relation to deuterated solvents. The following abbreviations were used for ^1H NMR spectra to indicate the signal multiplicity: s (singlet), d (doublet), t (triplet), q (quartet) and m (multiplet) as well as combinations of them. For HRMS data, the ESI-positive method was applied on the Agilent G6520 Q-TOF, or EI method was applied on the Themo Fisher Scientific Thermo DFS. Chemicals were purchased from commercial suppliers. Unless stated otherwise, all the substrates and solvents were purified and dried according to standard methods prior to use.

General Procedure for C-H activation

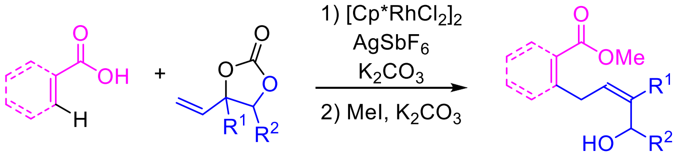

1 **2** **3**

A teflon-capped vial was charged with the respective benzoic acid (0.1 mmol, 1.0 equiv.), vinylethylene carbonate (0.15 mmol, 1.5 equiv.), K_2CO_3 (0.05 mmol, 0.5 equiv.), $[Cp^*RhCl_2]_2$ (5 mol%), $AgSbF_6$ (20 mol%) under an air atmosphere. The reaction mixture was mixed uniformly, otherwise it could affect the *Z/E* ratio and yield,

then stirred at 60 °C for 12 h. After that, the vial was cooled to r.t., added K_2CO_3 (0.3 mmol, 3.0 equiv.) and MeI (0.6 mmol, 0.6 equiv.), then diluted with DMF (1 mL). The mixture was stirred at r.t. for 3 h, and then quenched with saturated brine and extracted with ethyl acetate. The combined organic layers were dried over anhydrous Na_2SO_4, concentrated in vacuo to give the residue. The ratio of Z/E-isomers were determined by ^{1}H NMR analysis of crude reaction mixture. The crude residue was purified by FCC (PE/EA = 3:1, R_f = 0.2~0.4) to get the target product as light oil.

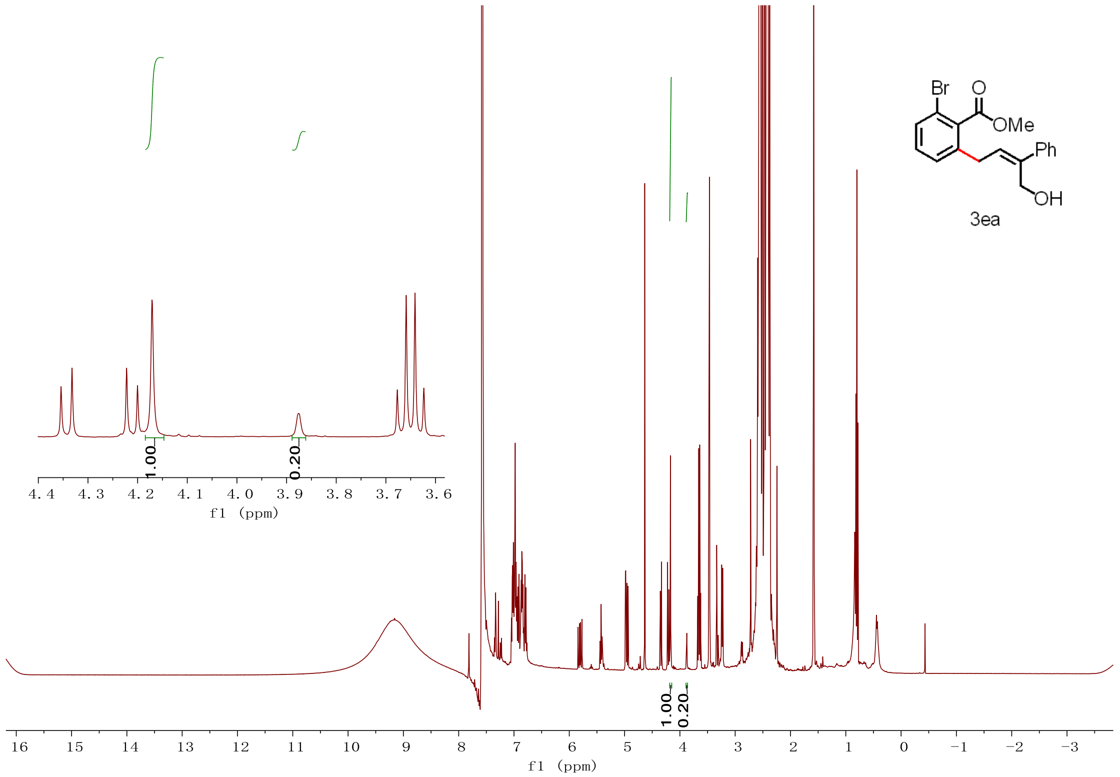

Supplementary Figure 4 The ^{1}H NMR analysis of crude reaction mixture of **3ea**. The ratio of *Z/E* isomers of **3ea** is 5:1.

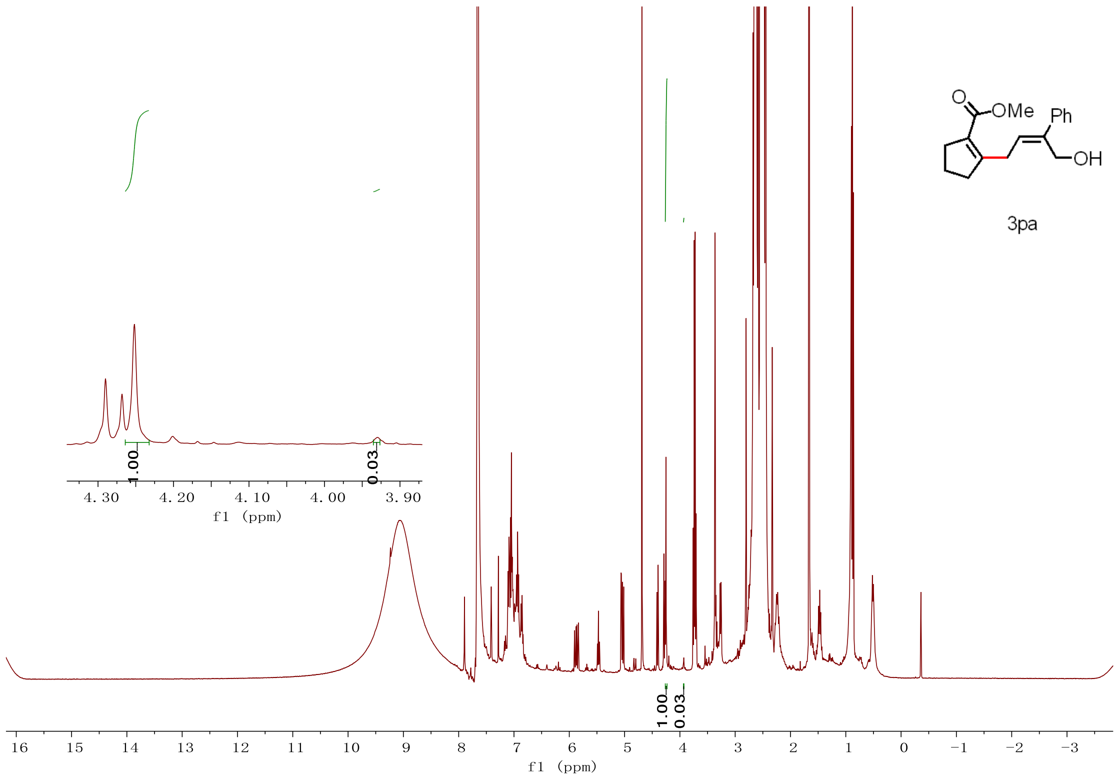

Supplementary Figure 5 The ^{1}H NMR analysis of crude reaction mixture of **3pa**. The ratio of *Z/E* isomers of **3pa** is >20:1.

Reaction in gram scale of 3a

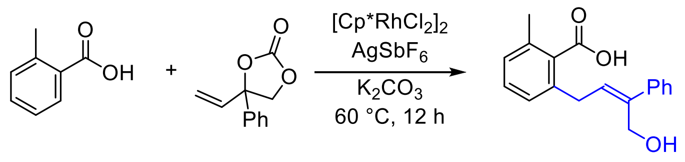

A teflon-capped vial was charged with the respective benzoic acid (5 mmol, 1.0 equiv.), vinylethylene carbonate (7.5 mmol, 1.5 equiv.), K_2CO_3 (2.5 mmol, 0.5 equiv.), $[Cp^*RhCl_2]_2$ (5 mol%), $AgSbF_6$ (20 mol%) under an air atmosphere. The reaction mixture was mixed uniformly, and then stirred at 60 °C for 12 h. After that, the vial was cooled to r.t., and then quenched with saturated brine and extracted with ethyl acetate. The combined organic layer was dried over anhydrous Na_2SO_4, concentrated in vacuo to give the residue. The crude residue was purified by FCC (PE/EA = 1:1, R_f = 0.1) to get the target product as light oil (1.03 g, 73%).

H/D exchange experiment

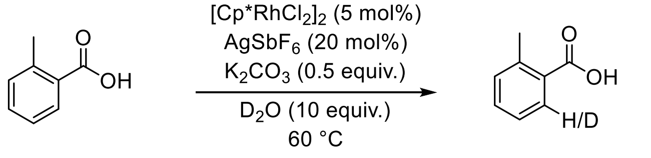

1a **1a-d:** 52% (75% D)

A teflon-capped vial was charged with 2-methyl benzoic acid (0.1 mmol, 1 equiv.), D_2O (1.0 mmol, 10 equiv.), K_2CO_3 (0.05 mmol, 0.5 equiv.), $[Cp^*RhCl_2]_2$ (5 mol%), $AgSbF_6$ (20 mol%) under an air atmosphere. The reaction mixture was mixed uniformly, and then stirred at 60 °C for 2 h. The reaction mixture was quenched with saturated aqueous NaCl and extracted with ethyl acetate. The combined organic layer was dried over anhydrous Na_2SO_4, concentrated in vacuo to give the residue. The crude residue was purified by FCC to get the product. The ratio was identified by ^{1}HNMR.

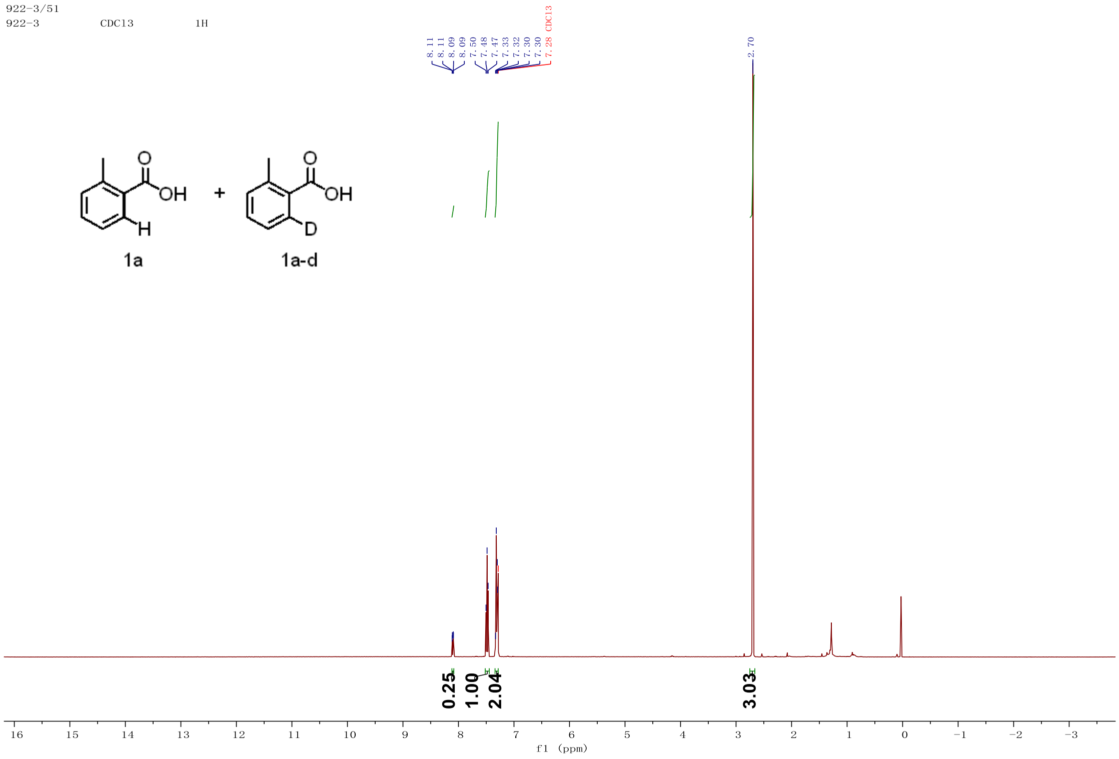

Supplementary Figure 6 The ^{1}H NMR analysis of **1a** and **1a-d**. The Rh (III)-catalyzed C−H activation allylation reaction was carried out in the presence of isotopically

labelled D_2O, and a significant 75% deuterium was incorporated at the ortho-position of **1a**.

Kinetic Isotope Effect

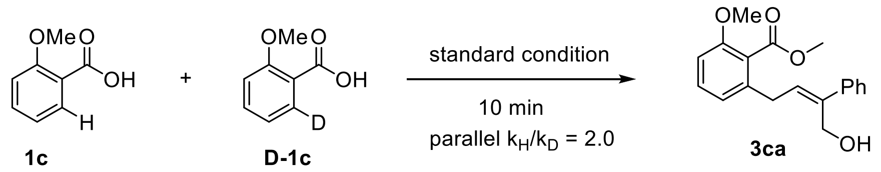

A teflon-capped vial was charged with the 2-methoxyl benzoic acid or 2-tetradeuterio-6-methoxyl benzoic acid (0.1 mmol, 1.0 equiv.), vinylethylene carbonate (0.15 mmol, 1.5 equiv.), K_2CO_3 (0.05 mmol, 0.5 equiv.), $[Cp^*RhCl_2]_2$ (5 mol%), $AgSbF_6$ (20 mol%) under an air atmosphere. The reaction mixture was mixed uniformly, and then stirred at 60 °C for 10 min. After that, the vial was cooled to r.t., added K_2CO_3 (0.3 mmol, 3.0 equiv.) and MeI (0.6 mmol, 0.6 equiv.), then diluted with DMF (1 mL). The mixture was stirred at r.t. for 3 h, and then quenched with saturated brine and extracted with ethyl acetate. The combined organic layer was dried over anhydrous Na_2SO_4, concentrated in vacuo to give the residue. The crude reaction mixture was diluted with $CDCl_3$, CH_2Br_2 as internal standard was added and the mixture was analyzed via ^1H NMR spectroscopy. The yields of **3aa** and **[D]-3aa** were obtained in 4.4% and 2.2% respectively (determined as an average of 2 runs), resulting in an k_H/k_D of 2.0.

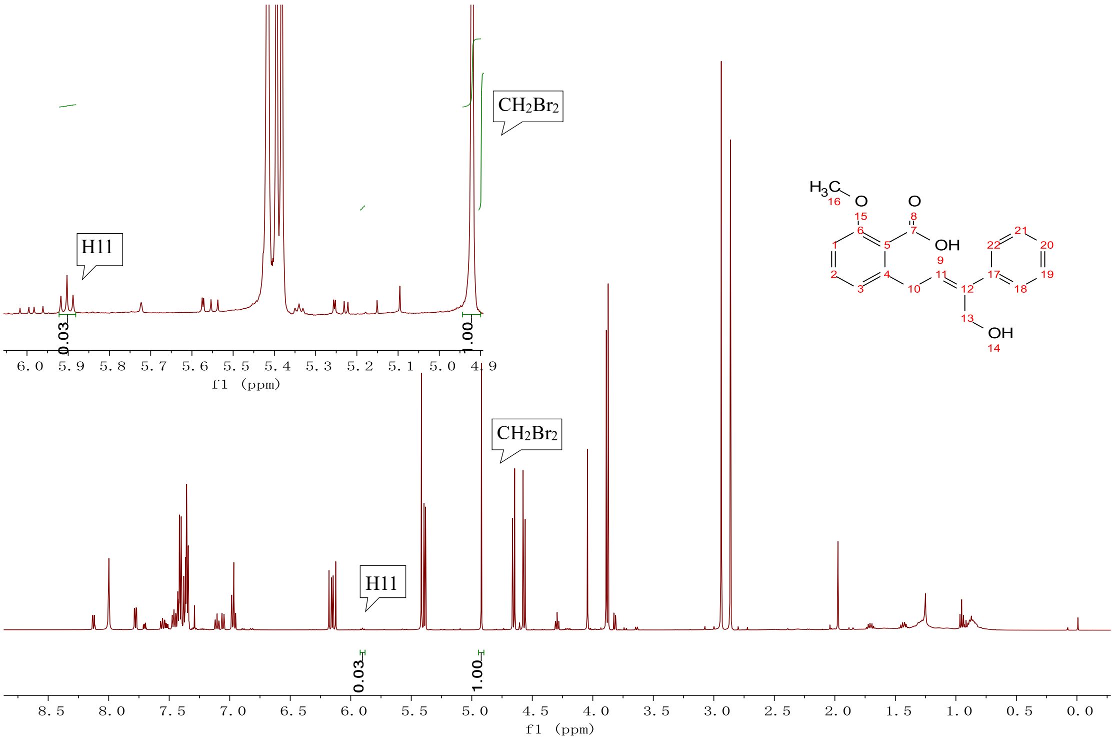

Supplementary Figure 7 The ^{1}H NMR analysis of reaction mixture of **3aa**. Yields were determined by the ^{1}H NMR using CH_2Br_2 as the internal standard, and the yield of **3aa** was obtained in 4.4%.

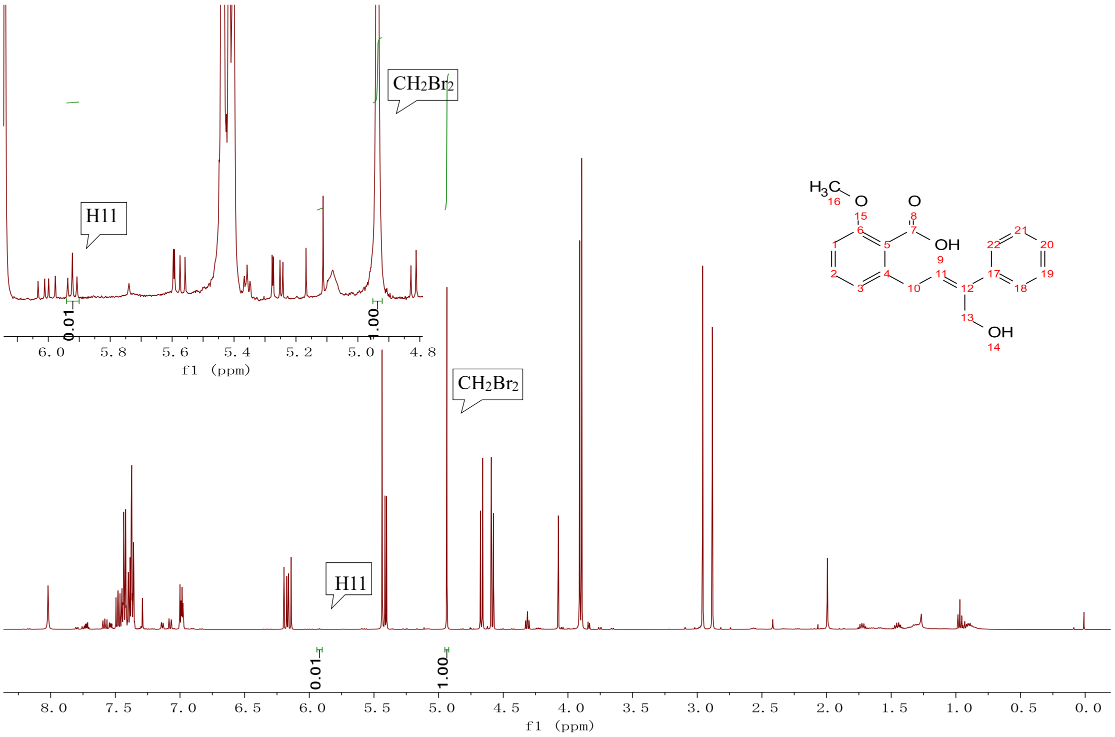

Supplementary Figure 8 The ^{1}H NMR analysis of reaction mixture of **[D]-3aa**. Yields were determined by the ^{1}H NMR using CH_2Br_2 as the internal standard, and the yield

of **[D]-3aa** was obtained in 2.2%.

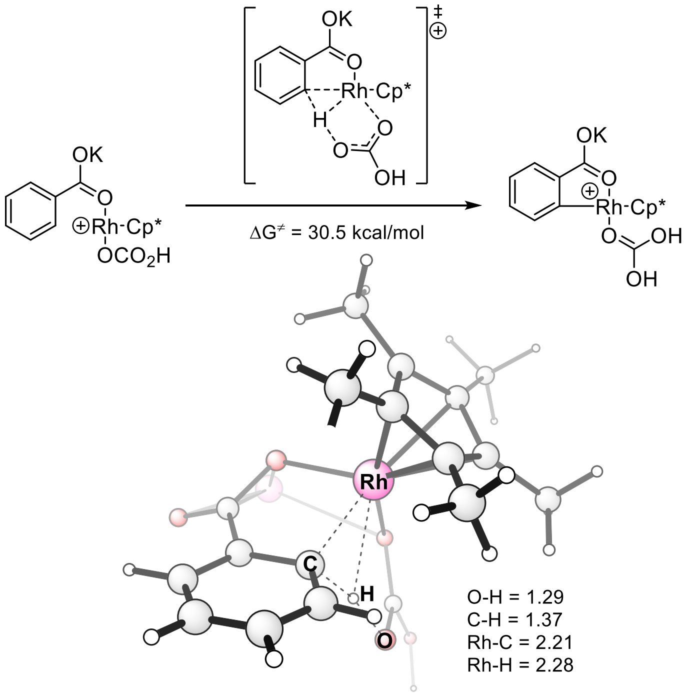

Supplementary Figure 9 DFT (at the B3LYP/6-31G*-LANL2DZ level) calculated free energy barrier (in kcal/mol) and TS structure (selected distances in Å) for the bicarbonate-assisted C-H activation via the CMD mechanism.[3,4]

General Procedure for the synthesis of 4

General procedure A:

To a solution of 3 (0.3 mmol) and the respective linear peptide (1.5 equiv.) in DMF (3 mL) was added EDCI (1.2 equiv.), HOBt (1.5 equiv.) and Et_3N (4.5 equiv.). After stirred overnight at room temperature, the mixture was partitioned between H_2O and EtOAc. The organic phase was washed with brine, then it was dried over anhydrous Na_2SO_4, filtered and concentrated to give the crude product. MeOH (3 mL) was added to give a solution, then LiOH (10 equiv.) in H_2O (3 mL) was added. The mixture was stirred at ambient temperature for 1 h. After removed solvent in vacuum, DCM was added and adjust pH = 3~4 by 1 N HCl. Hereafter, the resulting solution was concentrated under reduced pressure. The resulting product was dissolved in DMF: DCM (1:4, 0.5 mM), then EDCI (3.0 equiv.) and HOBt (3.0 equiv.) was added. After

stirred overnight at room temperature, solvent was removed at 30 °C. the crude residue was purified by flash column chromatography on silica gel (MeOH: DCM = 50:1, R_f = 0.5~0.7) to yield compounds as white solid.

General procedure B:

To a solution of 3 (0.3 mmol) and the respective linear peptide (1.5 equiv.) in DMF (3 mL) was added HATU (1.5 equiv.) and DIPEA (4.0 equiv.). After stirred overnight at room temperature, the mixture was partitioned between H_2O and EtOAc. The organic phase was washed with brine, then it was dried over anhydrous Na_2SO_4, filtered and concentrated to give the crude product. Next procedure followed procedure A.

General Procedure for the synthesis of 5a, 6a

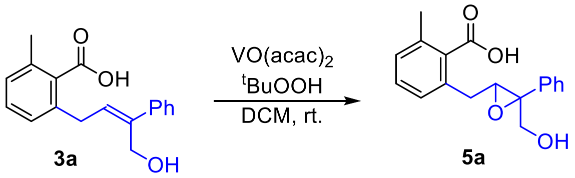

A screw-capped vial was charged with the **3a** (0.1 mmol, 1 equiv.), $VO(acac)_2$ (5 mol%) ^{t}BuOOH (0.05 mmol, 0.5 equiv.) and DCM (1.0 mL). The reaction mixture was stirred at r.t for 12 h under dark condition. The solvent was evaporated under reduced pressure and the residue was purified by FCC to get the target product **5a** as light oil.

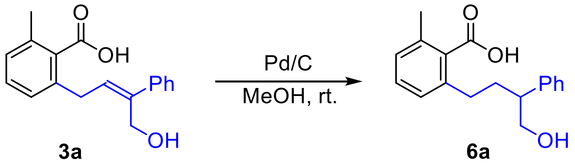

A screw-capped vial was charged with the **3a** (0.1 mmol, 1.0 equiv.), the Pd/C (10%) catalyst (10 mol%) and MeOH (1.0 mL). The reaction mixture was stirred for 5 h under H_2 at room temperature. After filtered, concentrated under reduced pressure, The mixture was purified by FCC to get the target product **6a** as light oil.

4-phenyl-4-vinyl-1,3-dioxolan-2-one (2a)

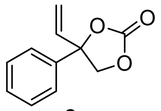

2a

^{1}H NMR (400 MHz, $CDCl_3$): δ 7.51 – 7.35 (m, 5H), 6.18 (dd, *J* = 17.2, 10.7 Hz, 1H), 5.49 – 5.40 (m, 2H), 4.68 (d, *J* = 8.4 Hz, 1H), 4.60 (d, *J* = 8.4 Hz, 1H).

4-(p-tolyl)-4-vinyl-1,3-dioxolan-2-one (2b)

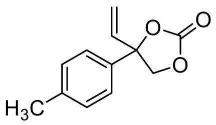

2b

^{1}H NMR (400 MHz, $CDCl_3$): δ 7.25 – 7.21 (m, 4 H), 6.14 (dd, *J* = 17.24, 10.72 Hz, 1 H), 5.43 – 5.39 (m, 2 H), 4.62 (d, *J* = 8.40 Hz, 1 H), 4.56 (d, *J* = 8.48 Hz, 1 H), 2.37 (s, 3 H).

4-(2-methoxyphenyl)-4-vinyl-1,3-dioxolan-2-one (2c)

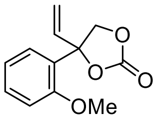

2c

^{1}H NMR (400 MHz, $CDCl_3$) δ 7.52 (dd, *J* = 7.7, 1.4 Hz, 1H), 7.40 – 7.34 (m, 1H), 7.04 (t, *J* = 7.6 Hz, 1H), 6.96 (d, *J* = 8.3 Hz, 1H), 6.25 (dd, *J* = 17.1, 10.7 Hz, 1H), 5.42 (d, *J* = 17.1 Hz, 1H), 5.27 (d, *J* = 10.7 Hz, 1H), 4.76 (d, *J* = 8.9 Hz, 1H), 4.56 (d, *J* = 8.9 Hz, 1H), 3.87 (s, 3H).

4-(3-methoxyphenyl)-4-vinyl-1,3-dioxolan-2-one (2d)

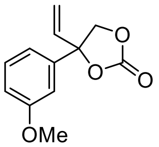

2d

^{1}H NMR (400 MHz, $CDCl_3$) δ 7.39 – 7.30 (m, 1H), 6.97 – 6.87 (m, 3H), 6.15 (dd, *J* = 17.2, 10.7 Hz, 1H), 5.44 (d, *J* = 9.7 Hz, 1H), 5.41 (d, *J* = 3.3 Hz, 1H), 4.66 (d, *J* = 8.5 Hz, 1H), 4.56 (d, *J* = 8.5 Hz, 1H), 3.82 (s, 3H).

4-(4-(benzyloxy)phenyl)-4-vinyl-1,3-dioxolan-2-one (2e)

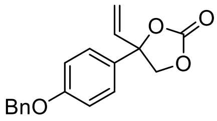

2e

^{1}H NMR (400 MHz, $CDCl_3$): δ 7.44 – 7.28 (m, 7 H), 7.03 – 6.99 (m, 2 H), 6.14 (dd, *J* = 17.08, 10.76 Hz, 1 H), 5.42 – 5.41 (m, 2 H), 5.08 (s, 2 H), 4.60 (d, *J* = 8.44 Hz, 1 H), 4.56 (d, *J* = 8.48 Hz, 1 H).

4-(4-fluorophenyl)-4-vinyl-1,3-dioxolan-2-one (2f)

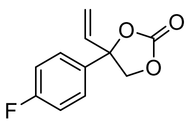

2f

^{1}H NMR (400 MHz, $CDCl_3$) δ 7.42 – 7.31 (m, 2H), 7.15 (t, *J* = 8.6 Hz, 2H), 6.16 (dd, *J* = 17.1, 10.7 Hz, 1H), 5.46 (s, 1H), 5.45 (d, *J* = 28.8 Hz, 1H), 4.66 (d, *J* = 8.5 Hz, 1H), 4.57 (d, *J* = 8.5 Hz, 1H).

4-(4-bromophenyl)-4-vinyl-1,3-dioxolan-2-one (2g)

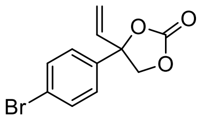

2g

^{1}H NMR (400 MHz, $CDCl_3$): δ 7.59 – 7.55 (m, 2 H), 7.25 – 7.22 (m, 2 H), 6.12 (dd, *J* = 17.16, 10.76 Hz, 1 H), 5.46 – 5.39 (m, 2 H), 4.65 (d, *J* = 8.52 Hz, 1 H), 4.53 (d, *J* = 8.52 Hz, 1 H).

4-(4-(trifluoromethyl)phenyl)-4-vinyl-1,3-dioxolan-2-one (2h)

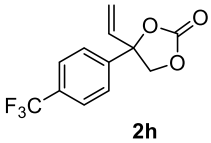

2h

^{1}H NMR (400 MHz, $CDCl_3$): δ 7.72 – 7.70 (m, 2 H), 7.51 – 7.49 (m, 2 H), 6.15 (dd, *J* = 17.20, 10.76 Hz, 1 H), 5.49 – 5.41 (m, 2 H), 4.70 (d, *J* = 8.52 Hz, 1 H), 4.56 (d, *J* = 8.56 Hz, 1 H).

4-(3-fluorophenyl)-4-vinyl-1,3-dioxolan-2-one (2i)

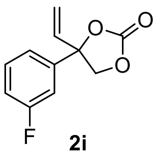

2i

^{1}H NMR (400 MHz, $CDCl_3$): δ 7.44 – 7.38 (m, 1 H), 7.14 – 7.06 (m, 3 H), 6.13 (dd, *J* = 17.12, 10.76 Hz, 1 H), 5.47 – 5.42 (m, 2 H), 4.65 (d, *J* = 8.48 Hz, 1 H), 4.55 (d, *J* = 8.52 Hz, 1 H).

4-(3,4-dichlorophenyl)-4-vinyl-1,3-dioxolan-2-one (2j)

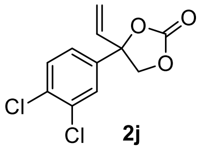

2j

^{1}H NMR (400 MHz, $CDCl_3$): δ 7.53 – 7.47 (m, 2 H), 7.22 – 7.19 (m, 1 H), 6.11 (dd, *J* = 17.12, 10.72 Hz, 1 H), 5.49 – 5.42 (m, 2 H), 4.65 (d, *J* = 8.60 Hz, 1 H), 4.52 (d, *J* = 8.56 Hz, 1 H).

4-(3-methoxyphenyl)-5-methyl-4-vinyl-1,3-dioxolan-2-one (2k)

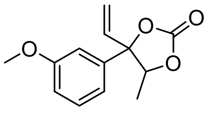

2k

^{1}H NMR (400 MHz, $CDCl_3$): δ 7.38 – 7.30 (m, 1H), 6.96 – 6.90 (m, 1H), 6.90 (d, *J* = 1.4 Hz, 1H), 6.89 – 6.84 (m, 1H), 6.25 (dd, *J* = 17.2, 10.9 Hz, 1H), 5.55 (d, *J* = 17.2 Hz, 1H), 5.44 (d, *J* = 10.8 Hz, 1H), 4.93 (q, *J* = 6.5 Hz, 1H), 3.84 (s, 3H), 1.07 (d, *J* = 6.5 Hz, 3H).

4-(thiophen-3-yl)-4-vinyl-1,3-dioxolan-2-one (2l)

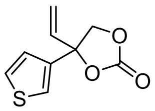

2l

^{1}H NMR (400 MHz, $CDCl_3$) δ 7.44 (dd, *J* = 5.0, 3.0 Hz, 1H), 7.37 (dd, *J* = 2.9, 1.3 Hz, 1H), 7.08 (dd, *J* = 5.1, 1.2 Hz, 1H), 6.19 (dd, *J* = 17.2, 10.7 Hz, 1H), 5.47 (dd, *J* = 14.0, 3.2 Hz, 2H), 4.59 (q, *J* = 8.5 Hz, 2H).

8b-vinyl-3a,4,5,8b-tetrahydro-[1,3]dioxolo[4,5-e]benzofuran-2-one (2m)

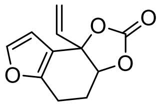

2m

^{1}H NMR (400 MHz, $CDCl_3$): δ 7.37 (d, *J* = 1.9 Hz, 1H), 6.37 (d, *J* = 2.0 Hz, 1H), 6.11 (dd, *J* = 17.2, 10.6 Hz, 1H), 5.48 (d, *J* = 10.7 Hz, 1H), 5.41 – 5.31 (m, 1H), 4.71 (dd, *J* = 4.3, 2.3 Hz, 1H), 2.90 (ddd, *J* = 16.5, 11.0, 5.3 Hz, 1H), 2.73 (ddd, *J* = 16.8, 6.2, 2.3 Hz, 1H), 2.52 (dddd, *J* = 15.1, 5.4, 4.2, 2.3 Hz, 1H), 2.01 (dddd, *J* = 15.0, 11.0, 6.2, 2.3 Hz, 1H).

Methyl (Z)-2-(4-hydroxy-3-phenylbut-2-en-1-yl)-6-methylbenzoate (3aa)

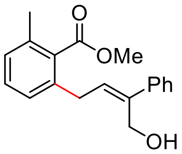

3aa

According to the general procedure, the product was yield 77% (>20:1). ^{1}H NMR (400 MHz, $CDCl_3$): δ 7.50 – 7.44 (m, 2H), 7.34 – 7.29 (m, 2H), 7.28 – 7.26 (m, 1H), 7.24 (d, *J* = 7.7 Hz, 1H), 7.11 (dd, *J* = 11.6, 7.6 Hz, 2H), 5.91 (t, *J* = 7.4 Hz, 1H), 4.62 (s, 2H), 3.83 (s, 3H), 3.68 (d, *J* = 7.4 Hz, 2H), 2.32 (s, 3H). ^{13}C NMR (101 MHz, $CDCl_3$): δ 170.87, 140.70, 139.46, 137.70, 135.59, 133.39, 129.87, 129.49, 128.54, 128.45, 127.27, 127.16, 126.32, 59.56, 52.25, 33.11, 19.86 HRMS (ESI) calcd. for $C_{19}H_{20}NaO_3$ [M+Na$^+$]: 319.1305, found: 319.1312.

Methyl (Z)-2-(4-hydroxy-3-phenylbut-2-en-1-yl)-5-methylbenzoate (3ba)

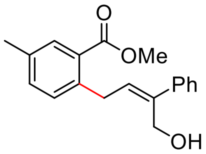

3ba

According to the general procedure, the product was yield 71% (>20:1). ^{1}H NMR (400 MHz, $CDCl_3$): δ 7.76 (d, *J* = 1.8 Hz, 1H), 7.56 – 7.46 (m, 2H), 7.33 (tt, *J* = 6.8, 0.9 Hz, 3H), 7.29 – 7.22 (m, 2H), 5.90 (t, *J* = 7.3 Hz, 1H), 4.69 (s, 2H), 4.00 (d, *J* = 7.2 Hz, 2H), 3.88 (s, 3H), 2.39 (s, 3H). ^{13}C NMR (101 MHz, $CDCl_3$): δ 168.26, 141.18, 139.26, 138.96, 136.17, 133.29, 131.37, 131.13, 130.42, 129.10, 128.36, 127.05, 126.28, 59.86, 52.20, 33.41, 20.85. HRMS (ESI) calcd. for $C_{19}H_{21}O_3$ [M+H$^+$]: 297.1485, found: 297.1481.

Methyl (Z)-2-(4-hydroxy-3-phenylbut-2-en-1-yl)-6-methoxybenzoate (3ca)

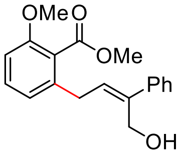

3ca

According to the general procedure, the product was yield 64% (>20:1). ^{1}H NMR (400 MHz, $CDCl_3$): δ 7.54 – 7.43 (m, 2H), 7.37 – 7.30 (m, 3H), 7.30 – 7.23 (m, 1H), 6.92 (d, *J* = 7.7 Hz, 1H), 6.85 (d, *J* = 8.3 Hz, 1H), 5.94 (t, *J* = 7.4 Hz, 1H), 4.64 (s, 2H), 3.86

(s, 3H), 3.85 (s, 3H), 3.67 (d, *J* = 7.4 Hz, 2H). ^{13}C NMR (101 MHz, $CDCl_3$): δ 169.31, 156.83, 140.67, 139.63, 139.14, 130.91, 129.07, 128.43, 127.28, 126.34, 123.26, 121.84, 109.40, 59.50, 55.99, 52.56, 32.71. HRMS (ESI) calcd. for $C_{19}H_{20}NaO_4$ [M+Na$^+$]: 335.1254, found: 335.1251.

Methyl (Z)-3-(4-hydroxy-3-phenylbut-2-en-1-yl)-[1,1'-biphenyl]-2-carboxylate (3da)

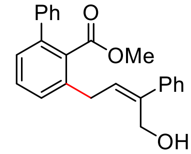

3da

According to the general procedure, the product was yield 49% (>20:1). ^{1}H NMR (400 MHz, $CDCl_3$): δ 7.52 – 7.48 (m, 2H), 7.46 (d, *J* = 7.7 Hz, 1H), 7.44 – 7.39 (m, 2H), 7.40 – 7.37 (m, 2H), 7.37 – 7.29 (m, 5H), 7.29 – 7.24 (m, 1H), 5.95 (t, *J* = 7.4 Hz, 1H), 4.67 (s, 2H), 3.82 (d, *J* = 7.4 Hz, 2H), 3.44 (s, 3H). ^{13}C NMR (101 MHz, $CDCl_3$): δ 170.93, 140.92, 140.79, 140.72, 139.64, 138.22, 132.66, 130.03, 129.17, 128.80, 128.42, 128.35, 128.15, 128.12, 127.46, 127.27, 126.31, 59.54, 52.23, 33.22. HRMS (ESI) calcd. for $C_{24}H_{22}NaO_3$ [M+Na$^+$]: 381.1461, found: 381.1465.

methyl (Z)-2-bromo-6-(4-hydroxy-3-phenylbut-2-en-1-yl)benzoate (3ea)

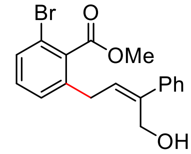

3ea

According to the general procedure, the product was yield 62% (5:1). ^{1}H NMR (400 MHz, $CDCl_3$): δ 7.52 – 7.46 (m, 3H), 7.39 – 7.33 (m, 2H), 7.31 – 7.27 (m, 2H), 7.26 (d, *J* = 7.5 Hz, 1H), 5.91 (t, *J* = 7.4 Hz, 1H), 4.64 (s, 2H), 3.90 (s, 3H), 3.70 (d, *J* = 7.5 Hz, 2H). ^{13}C NMR (101 MHz, $CDCl_3$): δ 168.77, 140.39, 140.21, 139.65, 135.50,

130.96, 130.85, 128.52, 128.48, 128.29, 127.48, 126.35, 119.79, 59.50, 52.81, 33.03. HRMS (ESI) calcd. for $C_{18}H_{17}NaO_3$ [M+Na$^+$]: 383.0253, found: 383.0264.

methyl 2,6-bis((Z)-4-hydroxy-3-phenylbut-2-en-1-yl)-4-methylbenzoate (3fa)

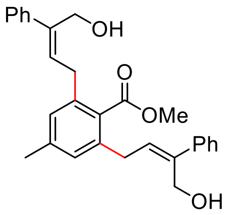

3fa

According to the general procedure, the product was yield 68%. ^{1}H NMR (400 MHz, $CDCl_3$): δ 7.51 – 7.47 (m, 4H), 7.39 – 7.32 (m, 4H), 7.30 – 7.26 (m, 2H), 7.02 (s, 2H), 5.94 (t, *J* = 7.3 Hz, 2H), 4.65 (s, 4H), 3.81 (s, 3H), 3.69 (d, *J* = 7.3 Hz, 4H), 2.36 (s, 3H), 2.15 – 1.89 (m, 1H). ^{13}C NMR (101 MHz, $CDCl_3$): δ 171.11, 140.61, 140.47, 139.50, 138.34, 130.20, 129.54, 128.79, 128.50, 127.33, 126.34, 59.61, 52.53, 33.07, 21.33. HRMS (ESI) calcd. for $C_{29}H_{30}NaO_4$ [M+Na$^+$]: 465.2036, found: 465.2030.

methyl 2,6-bis((Z)-4-hydroxy-3-phenylbut-2-en-1-yl)-3-methoxybenzoate (3ga)

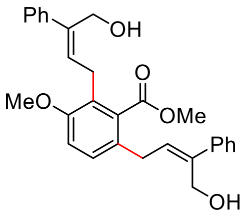

3ga

According to the general procedure, the product was yield 44%. ^{1}H NMR (400 MHz, $CDCl_3$): δ 7.51 – 7.44 (m, 4H), 7.38 – 7.34 (m, 2H), 7.34 – 7.30 (m, 2H), 7.30 – 7.28 (m, 1H), 7.27 – 7.24 (m, 1H), 7.18 (d, *J* = 8.4 Hz, 1H), 6.93 (d, *J* = 8.5 Hz, 1H), 5.93 (dt, *J* = 13.2, 7.4 Hz, 2H), 4.65 (d, *J* = 7.9 Hz, 4H), 3.87 (d, *J* = 2.8 Hz, 6H), 3.65 (t, *J* = 6.9 Hz, 4H). ^{13}C NMR (101 MHz, $CDCl_3$): δ 170.60, 155.89, 141.15, 140.65, 139.41, 138.88, 134.60, 129.71, 129.64, 129.48, 128.81, 128.50, 128.37, 127.31, 127.07, 126.33, 126.24, 126.00, 112.32, 59.87, 59.55, 55.94, 52.58, 32.33, 27.25. HRMS (ESI) calcd. for $C_{29}H_{30}NaO_5$ [M+Na$^+$]: 481.1985, found: 481.1977.

methyl 3,5-difluoro-2,6-bis((Z)-4-hydroxy-3-phenylbut-2-en-1-yl)benzoate (3ha)

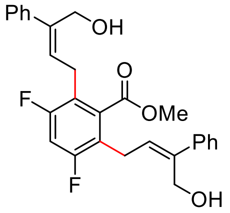

3ha

According to the general procedure, the product was yield 71%. ^{1}H NMR (400 MHz, $CDCl_3$): δ 7.49 – 7.43 (m, 4H), 7.38 – 7.32 (m, 4H), 7.30 – 7.27 (m, 2H), 6.95 (t, *J* = 9.5 Hz, 1H), 5.82 (t, *J* = 7.2 Hz, 2H), 4.66 (s, 4H), 3.88 (s, 3H), 3.67 (d, *J* = 7.3 Hz, 4H), 1.88 (s, 2H). ^{13}C NMR (101 MHz, $CDCl_3$): δ 168.59, δ 160.64 (d, *J* = 12.7 Hz), 158.18 (d, *J* = 12.9 Hz), 140.42, 139.85, 135.96 (t, *J* = 5.2 Hz) , 128.51, 127.77, 127.44, 126.37, 121.49 (d, *J* = 7.7 Hz), 121.35 (d, *J* = 7.8 Hz) , 105.36 (t, *J* = 26.7 Hz) , 59.52, 53.07, 25.58. HRMS (ESI) calcd. for $C_{28}H_{27}F_2O_4$ [M+H$^+$]: 465.1872, found: 465.1872.

methyl (Z)-5-(4-hydroxy-3-phenylbut-2-en-1-yl)benzo[d][1,3]dioxole-4-carboxylate (3ia)

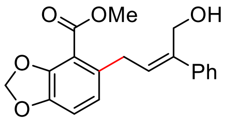

3ia

According to the general procedure, the product was yield 52% (16:1). ^{1}H NMR (400 MHz, $CDCl_3$): δ 7.52 – 7.46 (m, 2H), 7.38 – 7.30 (m, 2H), 7.29 – 7.24 (m, 1H), 6.88 (d, *J* = 7.9 Hz, 1H), 6.80 (d, *J* = 8.0 Hz, 1H), 6.06 (s, 2H), 5.85 (t, *J* = 7.2 Hz, 1H), 4.65 (d, *J* = 2.6 Hz, 2H), 3.89 (s, 3H), 3.85 (d, *J* = 7.2 Hz, 2H), 2.42 (s, 1H). ^{13}C NMR (101 MHz, $CDCl_3$): δ 166.31, 148.11, 146.85, 140.97, 139.25, 134.13, 130.24, 128.41, 127.16, 126.31, 123.32, 113.41, 110.97, 101.78, 59.70, 52.41, 33.06. HRMS (ESI) calcd. for $C_{19}H_{18}NaO_5$ [M+Na$^+$]: 349.1046, found: 349.1056.

methyl (Z)-2-(4-hydroxy-3-phenylbut-2-en-1-yl)-1-naphthoate (3ja)

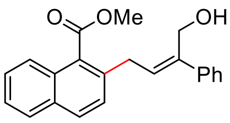

3ja

According to the general procedure, the product was yield 54% (>20:1). ^{1}H NMR (400 MHz, $CDCl_3$): δ 7.90 (d, J = 8.5 Hz, 1H), 7.89 – 7.80 (m, 2H), 7.56 (ddd, J = 8.4, 6.8, 1.6 Hz, 1H), 7.54 – 7.48 (m, 3H), 7.45 (d, J = 8.5 Hz, 1H), 7.37 – 7.31 (m, 2H), 7.30 – 7.27 (m, 1H), 6.03 (t, J = 7.4 Hz, 1H), 4.72 (s, 2H), 4.03 (s, 3H), 3.88 (d, J = 7.5 Hz, 2H), 2.19 (s, 1H). ^{13}C NMR (101 MHz, $CDCl_3$): δ 170.48, 140.65, 139.67, 135.93, 132.06, 130.42, 130.19, 130.04, 129.22, 128.49, 128.16, 127.52, 127.35, 127.26, 126.38, 125.96, 124.68, 59.66, 52.61, 33.41. HRMS (ESI) calcd. for $C_{22}H_{20}NaO_3$ [M+Na^{+}]: 355.1305, found: 355.1312.

(Z)-4-(1-methyl-1H-pyrrol-3-yl)-2-phenylbut-2-en-1-ol (3ka)

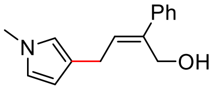

3ka

According to the general procedure, the product was yield 53% (8:1). ^{1}H NMR (400 MHz, $CDCl_3$): δ 7.59 – 7.47 (m, 2H), 7.44 – 7.33 (m, 2H), 7.29 (d, J = 7.5 Hz, 1H), 6.57 (t, J = 2.4 Hz, 1H), 6.47 (d, J = 2.0 Hz, 1H), 6.16 (t, J = 7.7 Hz, 1H), 6.05 (t, J = 2.2 Hz, 1H), 4.68 (s, 2H), 3.63 (s, 3H), 3.51 (d, J = 7.7 Hz, 2H). ^{13}C NMR (101 MHz, $CDCl_3$): δ 140.98, 138.49, 131.65, 128.46, 127.08, 126.32, 122.32, 121.96, 119.31, 108.16, 59.85, 36.10, 26.12. HRMS (ESI) calcd. for $C_{15}H_{18}NO$ [M+H^{+}]: 228.1383, found: 228.1384.

methyl (Z)-3-(4-hydroxy-3-phenylbut-2-en-1-yl)-1-methyl-1H-indole-2-carboxylate (3la)

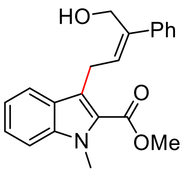

3la

According to the general procedure, the product was yield 62% (10:1). ^{1}H NMR (400 MHz, $CDCl_3$): δ 7.78 (dt, *J* = 8.1, 1.0 Hz, 1H), 7.49 – 7.44 (m, 2H), 7.41 (qd, *J* = 3.5, 3.0, 1.4 Hz, 2H), 7.34 – 7.28 (m, 2H), 7.26 – 7.22 (m, 1H), 7.22 – 7.18 (m, 1H), 6.00 (t, *J* = 7.3 Hz, 1H), 4.82 (s, 2H), 4.15 (d, *J* = 7.2 Hz, 2H), 4.03 (s, 3H), 3.99 (s, 3H). ^{13}C NMR (101 MHz, $CDCl_3$): δ 163.08, 141.05, 138.86, 138.23, 130.36, 128.95, 128.38, 127.04, 126.28, 125.66, 124.79, 123.47, 120.62, 120.21, 110.37, 59.89, 51.78, 32.41, 24.87. HRMS (ESI) calcd. for $C_{21}H_{21}NNaO_3$ [M+Na$^+$]: 358.1414, found: 358.1405.

methyl (Z)-2-(4-hydroxy-3-phenylbut-2-en-1-yl)-1-methyl-1H-indole-3-carboxylate (3ma)

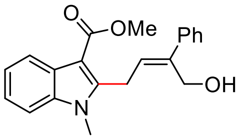

3ma

According to the general procedure, the product was yield 37% (>20:1). ^{1}H NMR (400 MHz, $CDCl_3$) δ 8.15 – 8.07 (m, 1H), 7.51 – 7.46 (m, 2H), 7.39 – 7.35 (m, 1H), 7.33 (td, *J* = 5.5, 4.9, 2.2 Hz, 2H), 7.31 – 7.28 (m, 2H), 7.26 (td, *J* = 5.3, 4.9, 2.4 Hz, 1H), 5.78 (t, *J* = 7.2 Hz, 1H), 4.76 (s, 2H), 4.32 (d, *J* = 7.3 Hz, 2H), 3.97 (s, 3H), 3.85 (s, 3H), 3.28 (s, 1H). ^{13}C NMR (101 MHz, $CDCl_3$) δ 166.77, 146.78, 141.08, 141.00, 136.71, 128.66, 128.41, 127.37, 126.32, 126.19, 124.96, 122.56, 122.06, 121.75, 109.42, 59.84, 51.12, 29.90, 25.25. HRMS (ESI) calcd. for $C_{21}H_{21}NNaO_3$ [M+Na$^+$]: 358.1414, found: 358.142.

methyl (Z)-2-(4-hydroxy-3-phenylbut-2-en-1-yl)cyclohex-1-ene-1-carboxylate (3na)

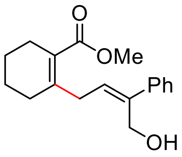

3na

According to the general procedure, the product was yield 76% (10:1). ^{1}H NMR (400 MHz, $CDCl_3$): δ 7.55 – 7.50 (m, 2H), 7.37 – 7.31 (m, 2H), 7.28 – 7.23 (m, 1H), 5.84 (t, *J* = 7.4 Hz, 1H), 4.61 (s, 2H), 3.73 (s, 3H), 3.43 (d, *J* = 7.4 Hz, 2H), 2.35 – 2.25 (m, 4H), 1.65 (p, *J* = 3.3 Hz, 4H). ^{13}C NMR (101 MHz, $CDCl_3$): δ 169.10, 148.58, 141.42, 139.60, 128.41, 128.38, 127.04, 126.23, 125.34, 59.85, 51.51, 34.84, 32.01, 26.50, 22.22, 22.20. HRMS (ESI) calcd. for $C_{18}H_{22}NaO_3$ [M+Na$^+$]: 309.1461, found: 309.1461.

methyl (Z)-5-(4-hydroxy-3-phenylbut-2-en-1-yl)-3,4-dihydro-2H-pyran-6-carboxylate (3oa)

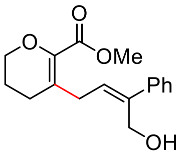

3oa

According to the general procedure, the product was yield 48% (>20:1). ^{1}H NMR (400 MHz, $CDCl_3$): δ 7.54 – 7.47 (m, 2H), 7.38 – 7.32 (m, 2H), 7.29 – 7.25 (m, 1H), 5.84 (t, *J* = 7.4 Hz, 1H), 4.62 (s, 2H), 4.12 – 4.00 (m, 2H), 3.82 (s, 3H), 3.47 (d, *J* = 7.4 Hz, 2H), 2.31 (t, *J* = 6.5 Hz, 2H), 1.97 – 1.89 (m, 2H). ^{13}C NMR (101 MHz, $CDCl_3$): δ 163.78, 141.24, 139.90, 139.64, 128.42, 128.31, 127.13, 126.25, 124.79, 65.99, 59.81, 52.14, 32.06, 27.11, 22.13. HRMS (ESI) calcd. for $C_{17}H_{20}NaO_4$ [M+Na$^+$]: 311.1254, found: 311.1251.

methyl (Z)-2-(4-hydroxy-3-phenylbut-2-en-1-yl)cyclopent-1-ene-1-carboxylate (3pa)

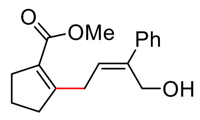

3pa

According to the general procedure, the product was yield 47% (>20:1). ^{1}H NMR (400 MHz, $CDCl_3$): δ 7.55 – 7.47 (m, 2H), 7.39 – 7.32 (m, 2H), 7.28 (d, J = 4.3 Hz, 1H), 5.86 (t, J = 7.8 Hz, 1H), 4.64 (s, 2H), 3.76 (s, 3H), 3.62 (dt, J = 7.8, 1.1 Hz, 2H), 2.64 (qd, J = 8.1, 7.6, 3.9 Hz, 4H), 1.92 – 1.84 (m, 2H). ^{13}C NMR (101 MHz, $CDCl_3$): δ 166.71, 157.71, 141.37, 140.33, 128.40, 127.68, 127.15, 126.82, 126.23, 59.81, 51.30, 38.40, 33.40, 29.70, 21.41. HRMS (ESI) calcd. for $C_{17}H_{21}O_3$ [M+H$^+$]: 273.1483, found: 273.1492.

methyl (S,Z)-2-ethoxy-6-(4-hydroxy-3-phenylbut-2-en-1-yl)-4-(2-((3-methyl-1-phenylbutyl)amino)-2-oxoethyl)benzoate (3qa)

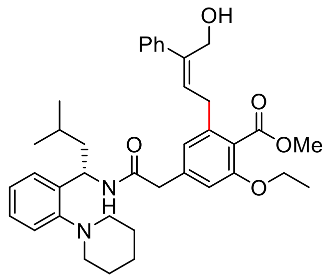

3qa

According to the general procedure, the product was yield 40% (10:1). ^{1}H NMR (400 MHz, $CDCl_3$): δ 7.53 – 7.43 (m, 2H), 7.36 – 7.30 (m, 2H), 7.29 – 7.23 (m, 2H), 7.23 – 7.17 (m, 2H), 7.10 (dd, J = 7.5, 1.5 Hz, 1H), 7.05 (ddd, J = 7.8, 6.1, 2.2 Hz, 1H), 6.75 (d, J = 16.6, 1.3 Hz, 2H), 5.88 (t, J = 7.4 Hz, 1H), 5.40 (td, J = 8.6, 6.5 Hz, 1H), 4.61 (d, J = 3.0 Hz, 2H), 4.06 – 3.90 (m, 2H), 3.83 (s, 3H), 3.63 (d, J = 7.4 Hz, 2H), 3.52 (s, 2H), 2.96 (d, J = 11.5 Hz,2H), 2.64 (d, J = 10.1 Hz,2H), 2.32 (s, 1H), 1.73 (dd, J = 7.5, 3.3 Hz, 3H), 1.66 – 1.50 (m, 5H), 1.43 (dt, J = 13.3, 6.6 Hz, 1H), 1.35 (t, J = 7.0 Hz, 3H), 0.93 (d, J = 6.5 Hz, 6H). ^{13}C NMR (101 MHz, $CDCl_3$): δ 169.27, 168.98, 156.73, 152.53, 140.66, 139.78, 139.59, 138.70, 138.40, 128.72, 128.42, 127.91, 127.72, 127.28, 126.33, 125.06, 122.81, 122.56, 122.44, 111.26, 64.46, 59.47, 52.41, 46.64,

44.18, 32.76, 26.75, 25.37, 22.77, 22.55, 14.65. HRMS (ESI) calcd. for $C_{38}H_{49}N_2O_5$ [M+H$^+$]: 613.3636, found: 613.3621.

methyl (Z)-2-(4-hydroxy-3-(p-tolyl)but-2-en-1-yl)-6-methylbenzoate (3ab)

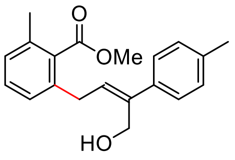

3ab

According to the general procedure, the product was yield 61% (>20:1). ^{1}H NMR (400 MHz, $CDCl_3$): δ 7.42 – 7.37 (m, 2H), 7.29 (t, *J* = 7.6 Hz, 1H), 7.18 – 7.10 (m, 4H), 5.91 (t, *J* = 7.4 Hz, 1H), 4.63 (s, 2H), 3.86 (s, 3H), 3.70 (d, *J* = 7.4 Hz, 2H), 2.35 (s, 6H). ^{13}C NMR (101 MHz, $CDCl_3$): δ 170.87, 139.32, 137.81, 137.72, 137.01, 135.54, 133.41, 129.84, 129.16, 128.56, 128.49, 127.15, 126.19, 59.52, 52.24, 33.06, 21.07, 19.84. HRMS (ESI) calcd. for $C_{20}H_{22}NaO_3$ [M+Na$^+$]: 333.1461, found: 333.1469.

methyl (Z)-2-(4-hydroxy-3-(2-methoxyphenyl)but-2-en-1-yl)-6-methylbenzoate (3ac)

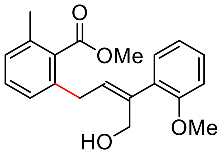

3ac

According to the general procedure, the product was yield 46% (>20:1). ^{1}H NMR (400 MHz, $CDCl_3$): δ 7.30 – 7.24 (m, 2H), 7.21 – 7.16 (m, 2H), 7.11 (d, *J* = 7.5 Hz, 1H), 6.97 – 6.89 (m, 2H), 5.75 (t, *J* = 7.3 Hz, 1H), 4.47 (d, *J* = 5.1 Hz, 2H), 3.92 (s, 3H), 3.90 (s, 3H), 3.69 (d, *J* = 7.2 Hz, 2H), 2.56 (t, *J* = 5.8 Hz, 2H), 2.36 (s, 3H). ^{13}C NMR (101 MHz, $CDCl_3$): δ 170.44, 156.30, 139.71, 137.75, 135.35, 133.59, 132.27, 131.68, 130.54, 129.69, 128.58, 128.25, 127.05, 121.19, 110.48, 60.82, 55.60, 52.08, 32.52, 19.80. HRMS (ESI) calcd. for $C_{20}H_{22}NaO_4$ [M+Na$^+$]: 349.141, found: 309.349.1403.

methyl (Z)-2-(4-hydroxy-3-(3-methoxyphenyl)but-2-en-1-yl)-6-methylbenzoate (3ad)

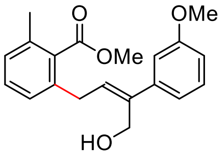

3ad

According to the general procedure, the product was yield 69% (8:1). ^{1}H NMR (400 MHz, $CDCl_3$): δ 7.31 – 7.23 (m, 2H), 7.14 (t, J = 7.7 Hz, 2H), 7.11 – 7.06 (m, 1H), 7.05 (t, J = 2.1 Hz, 1H), 6.83 (dd, J = 8.2, 2.5 Hz, 1H), 5.94 (t, J = 7.4 Hz, 1H), 4.62 (d, J = 4.0 Hz, 2H), 3.87 (s, 3H), 3.83 (s, 3H), 3.70 (d, J = 7.4 Hz, 2H), 2.35 (s, 3H), 2.18 (s, 1H). ^{13}C NMR (101 MHz, $CDCl_3$): δ 170.83, 159.72, 142.32, 139.43, 137.64, 135.58, 133.40, 129.86, 129.65, 129.40, 128.53, 127.14, 118.84, 112.67, 112.19, 59.61, 55.24, 52.23, 33.05, 19.85. HRMS (ESI) calcd. for $C_{20}H_{22}NaO_4$ [M+Na$^+$]: 349.141, found: 349.1409.

methyl (Z)-2-(3-(4-(benzyloxy)phenyl)-4-hydroxybut-2-en-1-yl)cyclohex-1-ene-1-carboxylate (3ne)

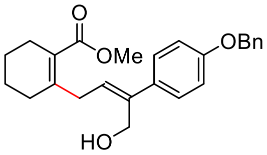

3ne

According to the general procedure, the product was yield 46% (>20:1). ^{1}H NMR (400 MHz, $CDCl_3$): δ 7.49 – 7.43 (m, 4H), 7.43 – 7.38 (m, 2H), 7.37 – 7.31 (m, 1H), 7.07 – 6.92 (m, 2H), 5.77 (t, J = 7.4 Hz, 1H), 5.09 (s, 2H), 4.58 (d, J = 4.8 Hz, 2H), 3.73 (s, 3H), 3.41 (d, J = 7.4 Hz, 2H), 2.63 (t, J = 6.3 Hz, 1H), 2.36 – 2.23 (m, 4H), 1.66 (s, 4H). ^{13}C NMR (101 MHz, $CDCl_3$): δ 169.13, 158.03, 148.68, 138.93, 137.05, 134.11, 128.57, 127.92, 127.44, 127.34, 126.91, 125.23, 114.76, 70.03, 59.82, 51.49, 34.78, 31.97, 26.51, 22.23, 22.21. HRMS (ESI) calcd. for $C_{25}H_{29}O_4$ [M+H$^+$]: 393.206, found: 393.2056.

methyl (Z)-2-(3-(4-fluorophenyl)-4-hydroxybut-2-en-1-yl)-6-methylbenzoate (3af)

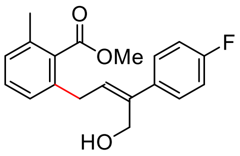

3af

According to the general procedure, the product was yield 76% (>20:1). ^{1}H NMR (400 MHz, $CDCl_3$): δ 7.50 – 7.42 (m, 2H), 7.29 (t, *J* = 7.7 Hz, 1H), 7.14 (dd, *J* = 7.6, 4.8 Hz, 2H), 7.05 – 6.97 (m, 2H), 5.88 (t, *J* = 7.4 Hz, 1H), 4.60 (s, 2H), 3.86 (s, 3H), 3.69 (d, *J* = 7.4 Hz, 2H), 2.35 (s, 3H). ^{13}C NMR (101 MHz, $CDCl_3$): δ 170.91, 162.19 (d, *J* = 246.0 Hz), 138.44, 137.60, 136.84 (d, *J* = 3.4 Hz), 135.66, 133.33, 129.92, 129.40, 128.61, 127.93 (d, *J* = 7.9 Hz), 127.18, 115.20 (d, *J* = 21.3 Hz), 59.53, 52.25, 33.14, 19.86. HRMS (ESI) calcd. for $C_{19}H_{19}FNaO_3$ [M+Na^{+}]: 337.121, found: 337.1212.

methyl (Z)-2-(3-(4-bromophenyl)-4-hydroxybut-2-en-1-yl)-6-methylbenzoate (3ag)

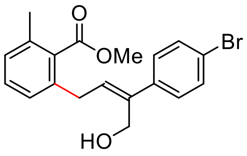

3ag

According to the general procedure, the product was yield 72% (10:1). ^{1}H NMR (400 MHz, $CDCl_3$): δ 7.49 – 7.42 (m, 2H), 7.41 – 7.33 (m, 2H), 7.29 (t, *J* = 7.7 Hz, 1H), 7.14 (d, *J* = 7.8 Hz, 2H), 5.93 (t, *J* = 7.4 Hz, 1H), 4.59 (s, 2H), 3.85 (s, 3H), 3.69 (d, *J* = 7.4 Hz, 2H), 2.35 (s, 3H). ^{13}C NMR (101 MHz, $CDCl_3$): δ 170.91, 139.73, 138.40, 137.45, 135.71, 133.31, 131.47, 130.01, 129.96, 128.68, 127.97, 127.21, 121.20, 59.32, 52.28, 33.21, 19.88. HRMS (ESI) calcd. for $C_{19}H_{19}BrNaO_3$ [M+Na^{+}]: 397.041, found: 397.041.

methyl (Z)-2-(4-hydroxy-3-(4-(trifluoromethyl)phenyl)but-2-en-1-yl)-6-methylbenzoate (3ah)

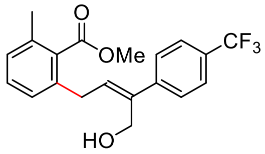

3ah

According to the general procedure, the product was yield 65% (10:1). ^{1}H NMR (400 MHz, $CDCl_3$): δ 7.66 – 7.54 (m, 4H), 7.31 (d, *J* = 7.6 Hz, 1H), 7.15 (dd, *J* = 7.7, 2.7 Hz, 2H), 6.01 (t, *J* = 7.4 Hz, 1H), 4.64 (s, 2H), 3.86 (s, 3H), 3.72 (d, *J* = 7.4 Hz, 2H), 2.43 (s, 1H), 2.35 (s, 3H). ^{13}C NMR (101 MHz, $CDCl_3$): δ 170.90, 144.48, 138.40, 137.27, 135.80, 133.30, 131.48, 130.01, 129.18 (d, *J* = 32.2 Hz), 128.76, 127.22, 126.57, 125.31 (q, *J* = 3.8 Hz), 59.30, 52.26, 33.26, 19.88. HRMS (ESI) calcd. for $C_{20}H_{18}F_3O_3$ [$M-H^+$]: 363.1214, found: 363.1205.

methyl (Z)-2-(3-(3-fluorophenyl)-4-hydroxybut-2-en-1-yl)-6-methylbenzoate (3ai)

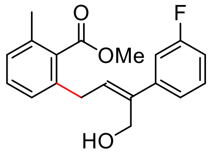

3ai

According to the general procedure, the product was yield 70% (13:1). ^{1}H NMR (400 MHz, $CDCl_3$): δ 7.33 – 7.26 (m, 3H), 7.22 (ddt, *J* = 10.6, 2.2, 1.1 Hz, 1H), 7.16 – 7.11 (m, 2H), 6.96 (ddt, *J* = 8.4, 7.1, 2.6 Hz, 1H), 5.96 (t, *J* = 7.4 Hz, 1H), 4.61 (s, 2H), 3.86 (s, 3H), 3.70 (d, *J* = 7.4 Hz, 2H), 2.35 (s, 3H). ^{13}C NMR (101 MHz, $CDCl_3$): δ 170.90, 162.96 (d, *J* = 245.1 Hz), 143.19 (d, *J* = 7.6 Hz), 138.42 (d, *J* = 1.8 Hz), 137.41, 135.71, 133.32, 130.52, 129.96, 129.80 (d, *J* = 8.4 Hz), 128.68, 127.20, 121.90, 114.01 (d, *J* = 21.2 Hz), 113.26 (d, *J* = 22.0 Hz), 59.38, 52.27, 33.16, 19.88. HRMS (ESI) calcd. for $C_{19}H_{19}FNaO_3$ [$M+Na^+$]: 337.121, found: 337.1205.

methyl (Z)-2-(3-(3,4-dichlorophenyl)-4-hydroxybut-2-en-1-yl)-6-methylbenzoate (3aj)

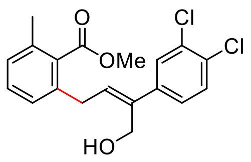

3aj

According to the general procedure, the product was yield 68% (10:1). ^{1}H NMR (400 MHz, $CDCl_3$): δ 7.60 (d, J = 2.1 Hz, 1H), 7.39 (d, J = 8.4 Hz, 1H), 7.36 – 7.31 (m, 1H), 7.29 (d, J = 7.7 Hz, 1H), 7.14 (d, J = 7.7 Hz, 2H), 5.94 (t, J = 7.4 Hz, 1H), 4.62 – 4.53 (m, 2H), 3.86 (s, 3H), 3.69 (d, J = 7.4 Hz, 2H), 2.42 (s, 1H), 2.35 (s, 3H). ^{13}C NMR (101 MHz, $CDCl_3$): δ 170.93, 141.00, 137.40, 137.21, 135.82, 133.26, 132.44, 131.07, 130.99, 130.23, 130.04, 128.80, 128.24, 127.25, 125.66, 59.19, 52.30, 33.25, 19.91. HRMS (ESI) calcd. for $C_{19}H_{17}Cl_2O_3$ [M+H$^+$]: 363.056, found: 363.0555.

methyl (Z)-2-(4-hydroxy-3-(3-methoxyphenyl)-2-methylbut-2-en-1-yl)-6-methylbenzoate (3ak)

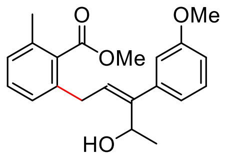

3ak

According to the general procedure, the product was yield 32% (>20:1). ^{1}H NMR (400 MHz, $CDCl_3$): δ 7.28 (d, J = 7.7 Hz, 1H), 7.22 (t, J = 7.9 Hz, 1H), 7.14 (d, J = 7.7 Hz, 1H), 7.11 (d, J = 7.5 Hz, 1H), 7.02 (dt, J = 7.6, 1.2 Hz, 1H), 7.00 (dd, J = 2.6, 1.6 Hz, 1H), 6.82 (ddd, J = 8.3, 2.6, 1.0 Hz, 1H), 5.63 (dd, J = 8.7, 6.0 Hz, 1H), 5.11 (q, J = 6.6, 5.9 Hz, 1H), 3.92 (s, 3H), 3.89 – 3.84 (m, 1H), 3.82 (s, 3H), 3.54 (dd, J = 15.8, 6.0 Hz, 1H), 2.38 (s, 1H), 2.34 (s, 3H), 1.32 (d, J = 6.6 Hz, 3H). ^{13}C NMR (101 MHz, $CDCl_3$): δ 170.78, 159.15, 144.14, 142.16, 137.91, 135.53, 133.32, 129.81, 129.05, 128.84,

128.40, 126.98, 120.85, 114.15, 112.35, 65.75, 55.20, 52.16, 32.35, 21.87, 19.86. HRMS (ESI) calcd. for $C_{21}H_{24}NaO_4$ [M+Na$^+$]: 363.1567, found: 363.1564.

methyl (Z)-2-(4-hydroxy-3-(thiophen-3-yl)but-2-en-1-yl)-6-methylbenzoate (3al)

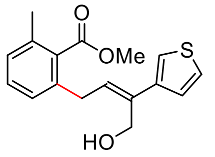

3al

According to the general procedure, the product was yield 59% (15:1). ^{1}H NMR (400 MHz, $CDCl_3$): δ 7.39 (dd, *J* = 2.9, 1.4 Hz, 1H), 7.31 – 7.23 (m, 3H), 7.13 (t, *J* = 6.8 Hz, 2H), 6.02 (t, *J* = 7.5 Hz, 1H), 4.58 (s, 2H), 3.83 (s, 3H), 3.69 (d, *J* = 7.4 Hz, 2H), 2.34 (s, 3H). ^{13}C NMR (101 MHz, $CDCl_3$): δ 170.96, 141.77, 137.58, 135.55, 134.47, 133.40, 129.85, 128.55, 127.94, 127.20, 125.62, 125.52, 120.55, 59.62, 52.23, 32.85, 19.81. HRMS (ESI) calcd. for $C_{17}H_{18}NaO_3S$ [M+Na$^+$]: 325.0869, found: 325.0870.

methyl (Z)-2-(2-(5-hydroxy-6,7-dihydrobenzofuran-4(5H)-ylidene)ethyl)-6-methylbenzoate (3am)

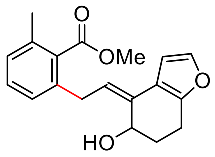

3am

According to the general procedure, the product was yield 32% (>20:1). ^{1}H NMR (400 MHz, $CDCl_3$): δ 7.36 (d, *J* = 2.0 Hz, 1H), 7.26 (t, *J* = 7.6 Hz, 1H), 7.11 (dd, *J* = 7.7, 4.3 Hz, 2H), 6.64 (d, *J* = 2.0 Hz, 1H), 5.56 (t, *J* = 7.1 Hz, 1H), 4.40 (dd, *J* = 5.8, 2.4 Hz, 1H), 3.79 (s, 3H), 3.77 – 3.64 (m, 2H), 2.96 (ddd, *J* = 16.2, 9.6, 5.9 Hz, 1H), 2.76 (ddd, *J* = 17.0, 6.3, 3.8 Hz, 1H), 2.34 (s, 3H), 2.17 (dtd, *J* = 13.3, 5.7, 3.8 Hz, 1H), 1.97 (dddd, *J* = 13.3, 9.2, 6.3, 2.6 Hz, 1H), 1.73 (s, 1H). ^{13}C NMR (101 MHz, $CDCl_3$): δ 170.44, 153.02, 141.46, 137.69, 135.32, 133.70, 133.15, 129.64, 128.26, 126.64, 123.15,

114.91, 109.33, 71.96, 52.03, 32.70, 29.83, 19.65, 19.55. HRMS (ESI) calcd. for $C_{19}H_{20}NaO_4$ [M+Na$^+$]: 335.1254, found: 335.1261.

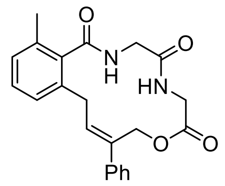

4a

According to the general procedure A, the product was yield 24%. ^{1}H NMR (500 MHz, DMSO-d_6): δ 8.80 (t, J = 6.4 Hz, 1H), 8.71 (t, J = 5.7 Hz, 1H), 7.35 (d, J = 7.6 Hz, 2H), 7.32 – 7.18 (m, 5H), 7.11 (d, J = 7.6 Hz, 1H), 5.92 (t, J = 5.3 Hz, 1H), 4.83 (s, 2H), 3.84 (s, 2H), 2.22 (s, 3H). ^{13}C NMR (126 MHz, DMSO): δ 170.50, 170.38, 169.50, 141.04, 138.86, 137.93, 137.24, 133.90, 132.28, 129.10, 128.81, 128.13, 127.46, 127.19, 126.06, 62.83, 43.18, 42.68, 32.20, 19.40. HRMS (EI) calcd. for $C_{22}H_{22}N_2O_4$ [M]$^+$: 378.1571, found: 378.1574.

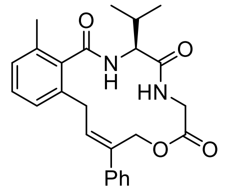

4b

According to the general procedure A, the product was yield 42%. ^{1}H NMR (500 MHz, DMSO-d_6): δ 8.91 (t, J = 5.8 Hz, 1H), 8.56 (d, J = 9.1 Hz, 1H), 7.34 (d, J = 7.8 Hz, 2H), 7.32 – 7.20 (m, 4H), 7.16 (d, J = 7.6 Hz, 1H), 7.11 (d, J = 7.6 Hz, 1H), 5.91 (dd, J = 6.6, 4.0 Hz, 1H), 4.88 (d, J = 11.9 Hz, 1H), 4.77 (d, J = 11.9 Hz, 1H), 4.22 (t, J = 9.5 Hz, 1H), 3.87 – 3.73 (m, 2H), 3.61 (dd, J = 18.0, 6.7 Hz, 1H), 3.50 (dd, J = 18.0, 4.0 Hz, 1H), 2.25 (s, 3H), 1.97 – 1.87 (m, 1H), 0.95 (d, J = 6.5 Hz, 3H), 0.85 (d, J = 6.6 Hz, 3H). ^{13}C NMR (126 MHz, DMSO): δ 172.68, 170.49, 168.76, 140.94, 139.29, 137.66, 137.15, 133.98, 132.60, 128.90, 128.81, 128.06, 127.47, 127.06, 125.99, 62.75, 58.95, 43.12, 32.11, 29.38, 19.81, 19.48. HRMS (EI) calcd. for $C_{25}H_{28}N_2O_4$ [M]$^+$: 420.2047, found: 420.2044.

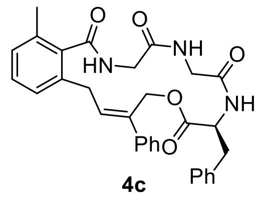

4c

According to the general procedure B, the product was yield 25%. ^{1}H NMR (500 MHz, DMSO-d_6): δ 8.89 (t, J = 6.1 Hz, 1H), 8.54 – 8.45 (m, 1H), 7.84 (t, J = 5.1 Hz, 1H), 7.39 – 7.32 (m, 4H), 7.27 (ddt, J = 12.9, 7.4, 1.5 Hz, 4H), 7.24 – 7.17 (m, 3H), 7.10 (dd, J = 12.3, 7.6 Hz, 2H), 6.21 (t, J = 7.1 Hz, 1H), 4.96 – 4.87 (m, 2H), 4.31 (dt, J = 8.9, 6.4 Hz, 1H), 3.79 (dd, J = 16.0, 5.4 Hz, 1H), 3.62 – 3.50 (m, 3H), 3.04 (dd, J = 13.8, 5.9 Hz, 1H), 2.95 (dd, J = 13.8, 9.0 Hz, 1H), 2.24 (s, 3H). ^{13}C NMR (126 MHz, DMSO) δ 171.26, 170.20, 169.80, 169.26, 140.98, 138.00, 137.74, 137.28, 135.09, 134.35, 134.13, 129.63, 129.06, 128.96, 128.76, 128.08, 127.68, 127.00, 126.55, 126.13, 62.58, 55.39, 43.26, 42.74, 36.56, 31.72, 19.43. HRMS (EI) calcd. for $C_{31}H_{31}N_3O_5$ $[M]^+$: 525.2254, found: 525.2258.

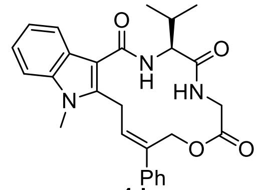

4d

According to the general procedure A, the product was yield 28%. ^{1}H NMR (600 MHz, DMSO-d_6): δ 8.96 (t, J = 5.6 Hz, 1H), 8.80 (d, J = 9.7 Hz, 1H), 7.67 (dt, J = 8.0, 1.0 Hz, 1H), 7.50 (dd, J = 8.4, 1.0 Hz, 1H), 7.32 – 7.25 (m, 5H), 7.23 – 7.19 (m, 1H), 7.14 (ddd, J = 7.9, 7.0, 1.0 Hz, 1H), 5.96 (dd, J = 8.0, 3.9 Hz, 1H), 5.06 (d, J = 11.6 Hz, 1H), 4.81 (d, J = 11.7 Hz, 1H), 4.34 (t, J = 9.9 Hz, 1H), 3.94 – 3.86 (m, 2H), 3.82 (dt, J = 17.3, 5.4 Hz, 2H), 3.74 (s, 3H), 2.03 – 1.98 (m, 1H), 0.97 (d, J = 6.7 Hz, 3H), 0.89 (d, J = 6.7 Hz, 3H). ^{13}C NMR (151 MHz, DMSO): δ 171.96, 170.36, 161.89, 140.78, 137.37, 136.33, 133.02, 132.36, 128.93, 127.56, 126.39, 125.77, 123.63, 120.02, 119.95, 113.27, 110.67, 62.06, 58.94, 43.14, 31.16, 30.05, 23.68, 19.63, 19.34. HRMS (EI) calcd. for $C_{27}H_{29}N_3O_4$ $[M]^+$: 459.2160, found: 459.2153.

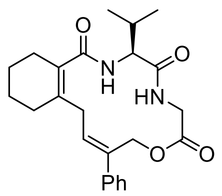

4e

According to the general procedure A, the product was yield 28%. ^{1}H NMR (500 MHz, DMSO-d_6): δ 8.70 (t, J = 5.8 Hz, 1H), 7.98 (d, J = 9.3 Hz, 1H), 7.39 – 7.29 (m, 4H), 7.27 – 7.22 (m, 1H), 5.98 (t, J = 5.5 Hz, 1H), 4.82 – 4.74 (m, 2H), 4.00 (t, J = 9.8 Hz, 1H), 3.88 (dd, J = 16.4, 5.5 Hz, 1H), 3.73 (dd, J = 16.4, 6.0 Hz, 1H), 3.17 (dd, J = 17.4, 6.1 Hz, 1H), 2.87 (dd, J = 17.5, 5.0 Hz, 1H), 2.19 (dd, J = 17.1, 6.0 Hz, 2H), 2.08 (s, 2H), 1.94 – 1.88 (m, 1H), 1.62 (dd, J = 16.4, 5.9 Hz, 4H), 0.85 (d, J = 6.6 Hz, 3H), 0.81 (d, J = 6.6 Hz, 3H). ^{13}C NMR (126 MHz, DMSO): δ 172.37, 170.80, 170.26, 141.15, 135.25, 133.33, 132.62, 132.57, 128.79, 127.38, 125.83, 62.51, 58.66, 42.82, 32.91, 29.25, 28.69, 27.21, 22.66, 22.21, 19.67, 19.42. HRMS (EI) calcd. for $C_{24}H_{30}N_2O_4$ $[M]^+$: 410.2200, found: 410.2200.

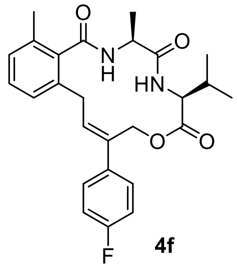

4f

According to the general procedure A, the product was yield 30%. ^{1}H NMR (600 MHz, DMSO-d_6): δ 8.69 (d, J = 8.3 Hz, 1H), 8.41 (d, J = 6.9 Hz, 1H), 7.35 (td, J = 8.2, 6.4 Hz, 1H), 7.27 (t, J = 7.6 Hz, 1H), 7.21 – 7.16 (m, 3H), 7.13 (d, J = 7.7 Hz, 1H), 7.07 (ddd, J = 10.0, 8.0, 2.6 Hz, 1H), 6.01 (dd, J = 6.4, 4.4 Hz, 1H), 4.83 (s, 2H), 3.96 (t, J = 7.0 Hz, 1H), 3.61 (dd, J = 30.6, 6.2 Hz, 1H), 3.57 – 3.47 (m, 2H), 2.24 (s, 3H), 2.04 (h, J = 6.8 Hz, 1H), 1.21 (d, J = 7.0 Hz, 3H), 0.96 (dd, J = 10.8, 6.8 Hz, 6H). ^{13}C NMR (151 MHz, DMSO): δ 173.34, 171.58, 168.85, 162.75 (d, J = 242.8 Hz), 143.47 (d, J = 7.7 Hz), 138.84, 138.44, 137.36, 134.06, 131.43, 130.73 (d, J = 8.5 Hz), 129.03, 128.19, 127.25, 121.98, 114.12 (d, J = 20.9 Hz), 112.66 (d, J = 22.2 Hz), 62.44, 60.34, 47.49, 32.25, 29.37, 19.51, 19.35, 19.27, 17.19. HRMS (EI) calcd. for $C_{26}H_{29}FN_2O_4$ $[M]^+$:

452.2113, found: 452.2106.

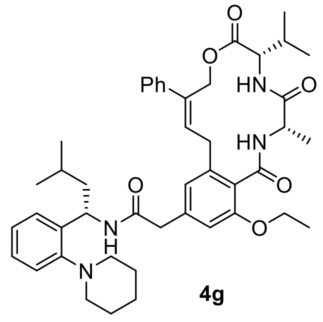

4g

According to the general procedure A, the product was yield 21%. ^{1}H NMR (600 MHz, DMSO-d_6): δ 8.45 (d, J = 8.6 Hz, 1H), 8.31 (d, J = 7.5 Hz, 1H), 7.97 (dd, J = 11.8, 8.5 Hz, 1H), 7.44 (dt, J = 8.1, 1.4 Hz, 2H), 7.30 (td, J = 7.8, 2.2 Hz, 3H), 7.24 – 7.20 (m, 1H), 7.13 (tt, J = 7.6, 2.0 Hz, 1H), 7.07 (dd, J = 8.0, 1.4 Hz, 1H), 7.02 – 6.96 (m, 1H), 6.79 (d, J = 7.1 Hz, 1H), 6.75 (d, J = 1.5 Hz, 1H), 5.88 (q, J = 7.7 Hz, 1H), 5.36 (q, J = 9.2 Hz, 1H), 4.58 – 4.50 (m, 1H), 4.41 (d, J = 6.2 Hz, 2H), 4.24 (ddd, J = 26.6, 8.5, 6.2 Hz, 1H), 3.97 (dt, J = 14.1, 7.1 Hz, 2H), 3.64 (s, 2H), 3.56 (td, J = 15.0, 7.6 Hz, 1H), 3.49 (dt, J = 15.2, 7.2 Hz, 1H), 3.40 (d, J = 3.1 Hz, 2H), 3.08 (s, 2H), 2.06 – 1.99 (m, 1H), 1.67 (d, J = 9.8 Hz, 2H), 1.62 – 1.52 (m, 3H), 1.49 (ddd, J = 14.3, 9.9, 4.8 Hz, 3H), 1.32 – 1.29 (m, 1H), 1.25 (ddt, J = 19.8, 7.1, 3.9 Hz, 6H), 0.88 (dt, J = 14.3, 6.4 Hz, 12H). ^{13}C NMR (151 MHz, DMSO): δ 172.88, 172.33, 169.43, 166.86, 155.56, 151.92, 141.94, 141.03, 139.69, 139.60, 139.08, 129.52, 129.43, 128.48, 127.60, 127.06, 126.60, 126.46, 124.43, 122.23, 120.91, 111.34, 64.26, 58.26, 57.69, 57.48, 52.23, 52.20, 48.95, 48.69, 47.06, 46.29, 43.15, 31.83, 31.74, 30.92, 30.60, 26.79, 25.33, 24.30, 23.69, 22.22, 19.39, 19.33, 18.86, 18.59, 18.47, 18.44, 15.06, 15.00. HRMS (ESI) calcd. for $C_{45}H_{59}N_4O_6$ [M+H$^+$]: 751.4429, found: 751.4429.

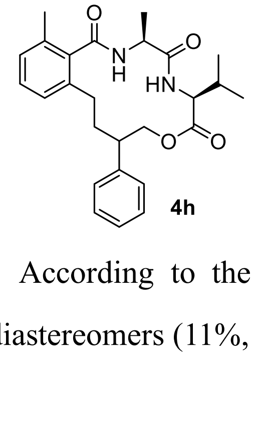

4h

According to the general procedure A, the product was yield as a mixture of diastereomers (11%, 1.2:1 dr). ^{1}H NMR (500 MHz, DMSO-d_6): δ 8.75 – 8.51 (m, 1H),

8.51 – 8.39 (m, 1H), 7.38 – 7.32 (m, 1H), 7.33 – 7.22 (m, 5H), 7.13 (dt, *J* = 10.9, 7.6 Hz, 1H), 7.04 (t, *J* = 8.1 Hz, 1H), 6.88 (d, *J* = 7.5 Hz, 1H), 5.01 – 4.78 (m, 1H), 4.34 – 3.94 (m, 3H), 2.96 – 2.67 (m, 2H), 2.42 (td, *J* = 13.0, 3.8 Hz, 1H), 2.24 (d, *J* = 11.8 Hz, 3H), 2.21 – 2.15 (m, 1H), 2.07 (dq, *J* = 50.9, 6.7 Hz, 1H), 1.75 – 1.51 (m, 1H), 1.26 (dd, *J* = 9.6, 6.9 Hz, 3H), 0.99 – 0.89 (m, 6H). ^{13}C NMR (126 MHz, DMSO): δ 172.79, 172.67, 171.17, 171.12, 169.46, 168.71, 143.03, 142.05, 139.03, 138.93, 138.00, 137.89, 134.32, 134.28, 129.09, 128.84, 128.79, 128.20, 128.07, 127.96, 127.82, 127.36, 127.19, 127.03, 126.95, 68.01, 67.41, 60.04, 58.87, 47.59, 47.40, 45.31, 44.09, 34.28, 32.40, 32.28, 29.71, 29.26, 19.59, 19.52, 19.39, 19.35, 19.28, 18.84, 17.06. HRMS (EI) calcd. for $C_{26}H_{32}N_2O_4$ $[M]^+$: 436.2361, found: 436.2357.

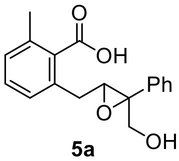

5a

According to the general procedure, the product was yield 57%. ^{1}H NMR (400 MHz, $CDCl_3$): δ 7.54 – 7.49 (m, 2H), 7.44 – 7.39 (m, 2H), 7.37 – 7.28 (m, 2H), 7.21 – 7.14 (m, 1H), 6.92 (d, *J* = 7.5 Hz, 1H), 4.82 (dd, *J* = 12.7, 2.5 Hz, 1H), 4.31 (d, *J* = 11.7 Hz, 1H), 3.87 (d, *J* = 11.6 Hz, 1H), 3.28 (dd, *J* = 16.5, 12.7 Hz, 1H), 2.66 (s, 3H), 2.32 (dd, *J* = 16.5, 2.5 Hz, 1H). ^{13}C NMR (101 MHz, $CDCl_3$): δ 164.62, 143.03, 140.61, 140.11, 133.01, 130.88, 128.68, 127.81, 125.64, 125.21, 122.78, 83.16, 75.93, 68.32, 28.28, 22.13. HRMS (ESI) calcd. for $C_{18}H_{19}O_4$ $[M+H^+]$: 299.1278, found: 299.1274.

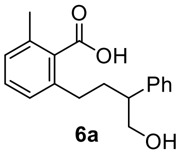

6a

According to the general procedure A, the product was yield 65%. ^{1}H NMR (400 MHz, $CDCl_3$): δ 7.35 (dd, *J* = 8.2, 6.9 Hz, 2H), 7.30 – 7.20 (m, 4H), 7.08 (d, *J* = 7.6 Hz, 1H), 6.99 (d, *J* = 7.6 Hz, 1H), 5.50 – 5.34 (m, 1H), 3.84 – 3.68 (m, 2H), 2.86 (dq, *J* = 8.8, 6.3 Hz, 1H), 2.65 (tt, *J* = 11.4, 7.4 Hz, 2H), 2.42 (s, 3H), 2.13 (ddt, *J* = 12.0, 9.9, 6.1 Hz, 1H), 2.02 – 1.88 (m, 1H). ^{13}C NMR (101 MHz, $CDCl_3$): δ 173.81, 141.95, 139.47,

135.27, 132.76, 129.67, 128.71, 128.07, 128.05, 127.07, 126.85, 67.12, 48.21, 34.01, 31.98, 20.05. HRMS (ESI) calcd. for $C_{18}H_{19}O_3$ [M-H$^-$]: 283.134, found: 283.134.

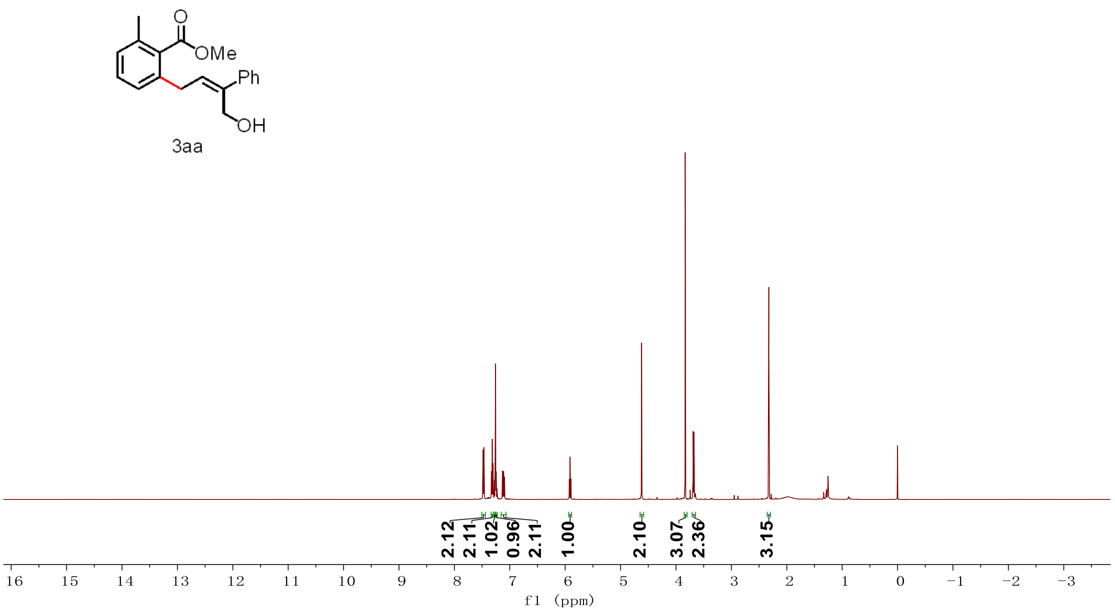

Supplementary Figure 10 ^{1}H NMR (400 MHz, $CDCl_3$) spectrum of compound **3aa**

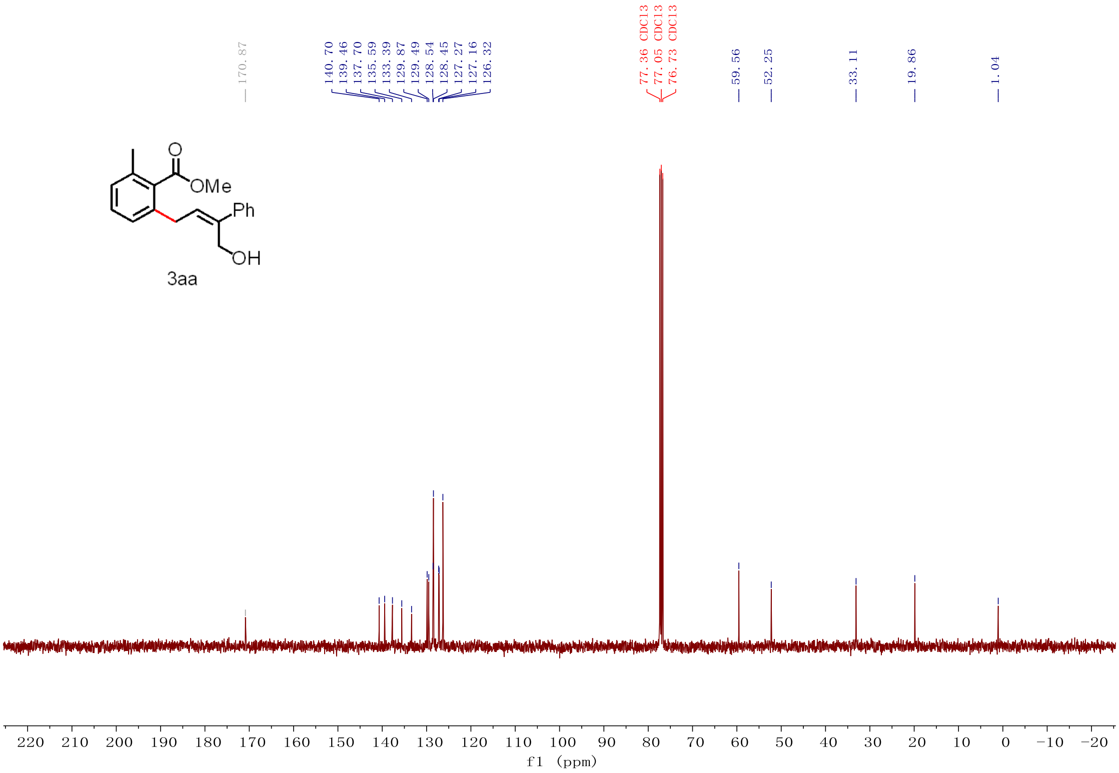

Supplementary Figure 11 ^{13}C NMR (101 MHz, $CDCl_3$) spectrum of compound **3aa**

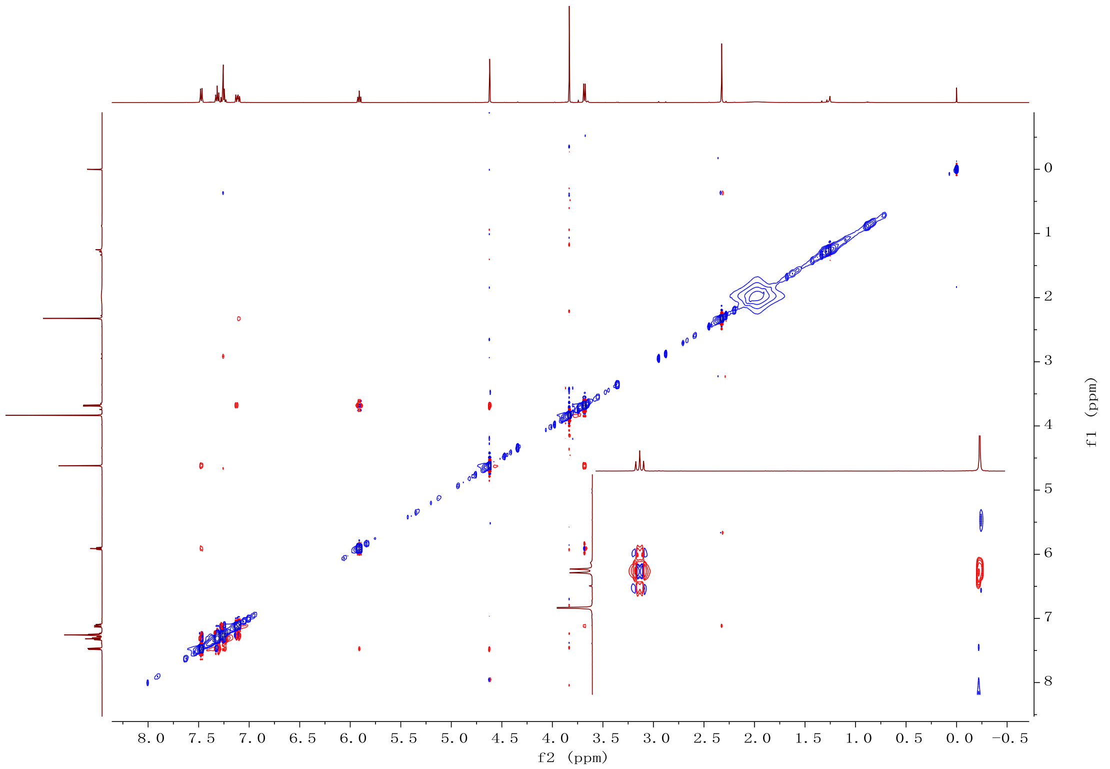

Supplementary Figure 12 NOE of compound **3aa**

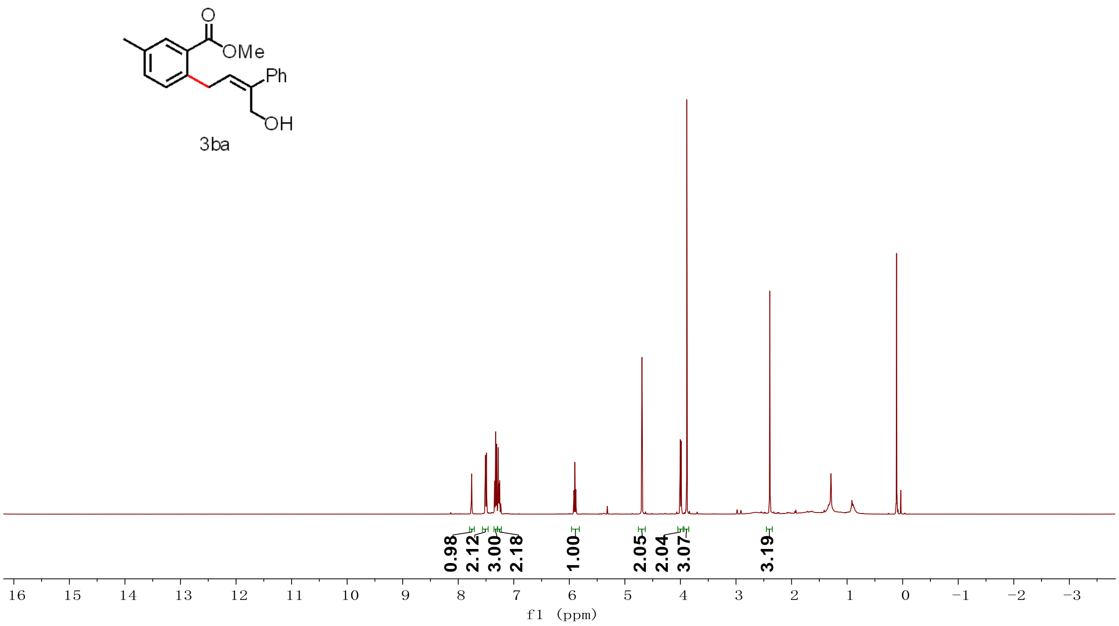

Supplementary Figure 13 ^{1}H NMR (400 MHz, $CDCl_3$) spectrum of compound **3ba**

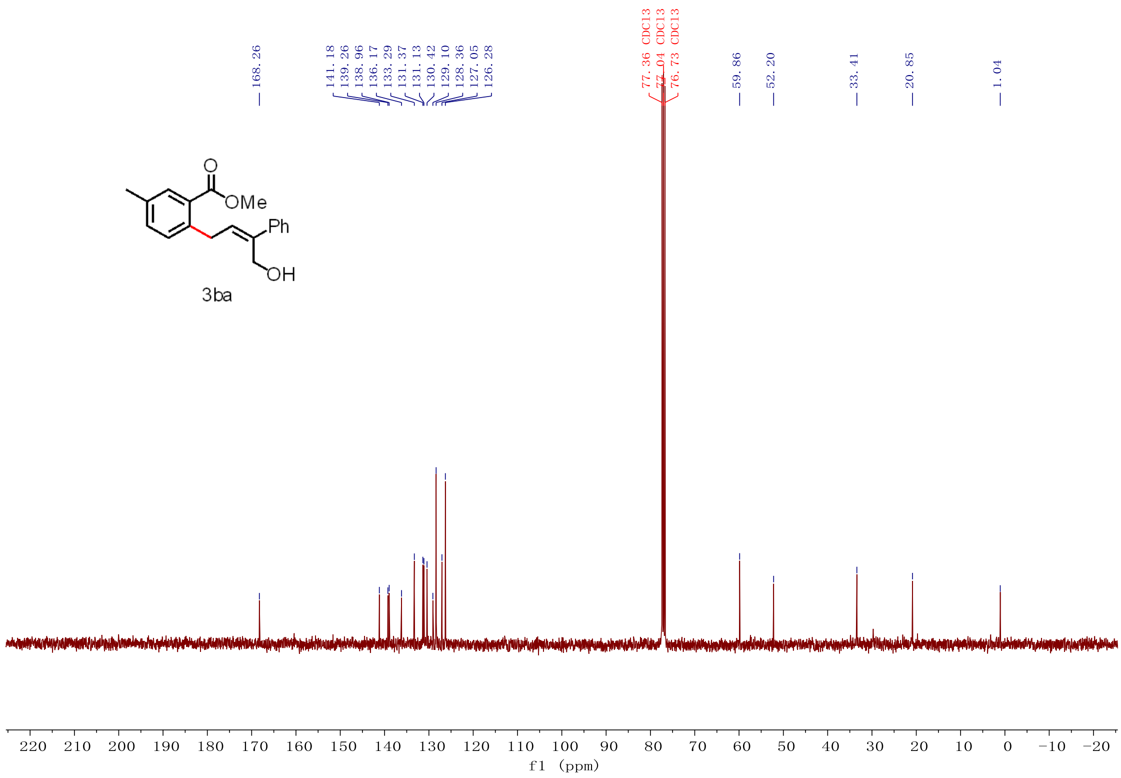

Supplementary Figure 14 ^{13}C NMR (101 MHz, $CDCl_3$) spectrum of compound **3ba**

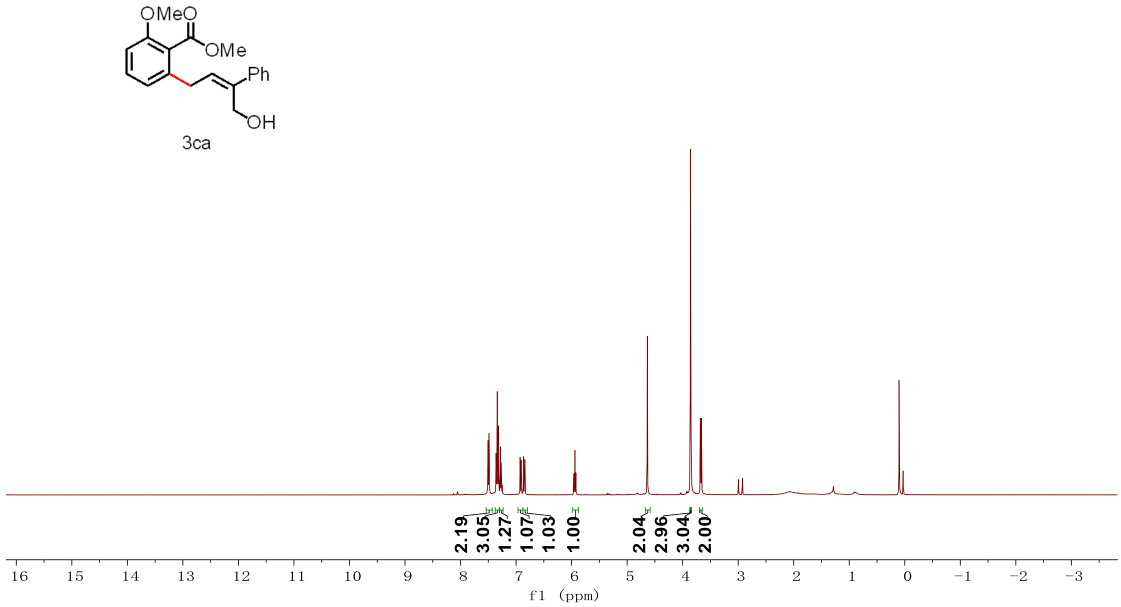

Supplementary Figure 15 ^{1}H NMR (400 MHz, $CDCl_3$) spectrum of compound **3ca**

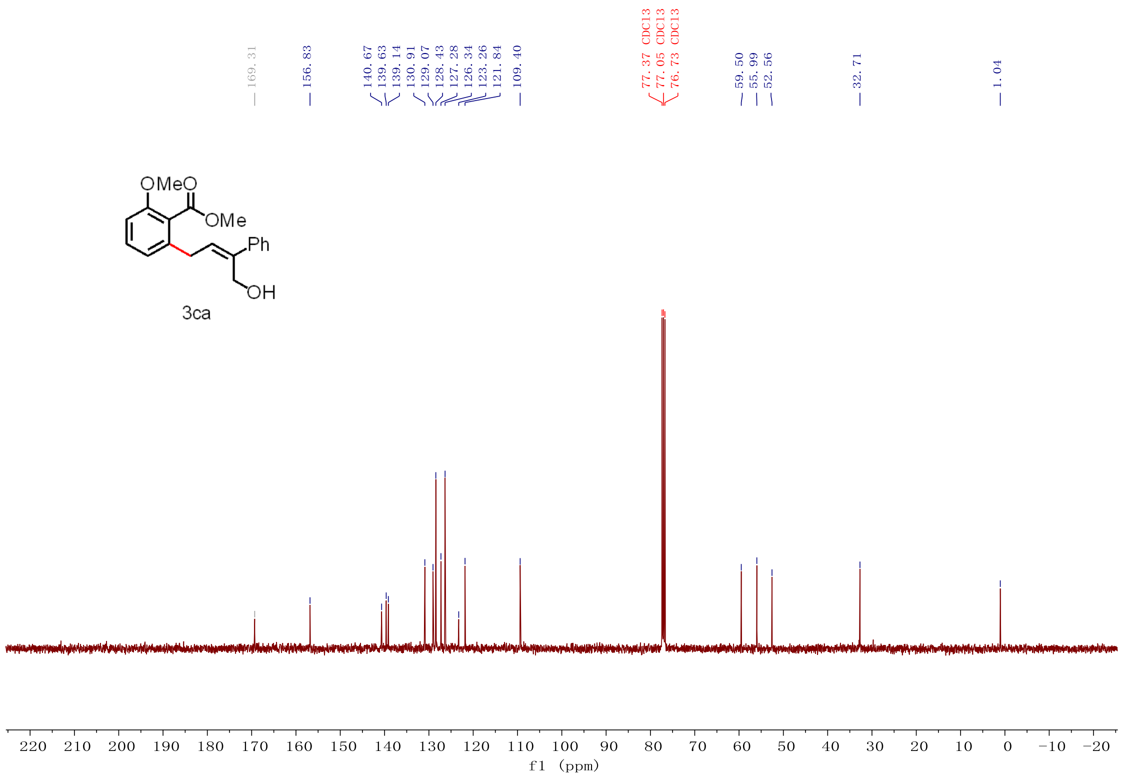

Supplementary Figure 16 ^{13}C NMR (101 MHz, $CDCl_3$) spectrum of compound **3ca**

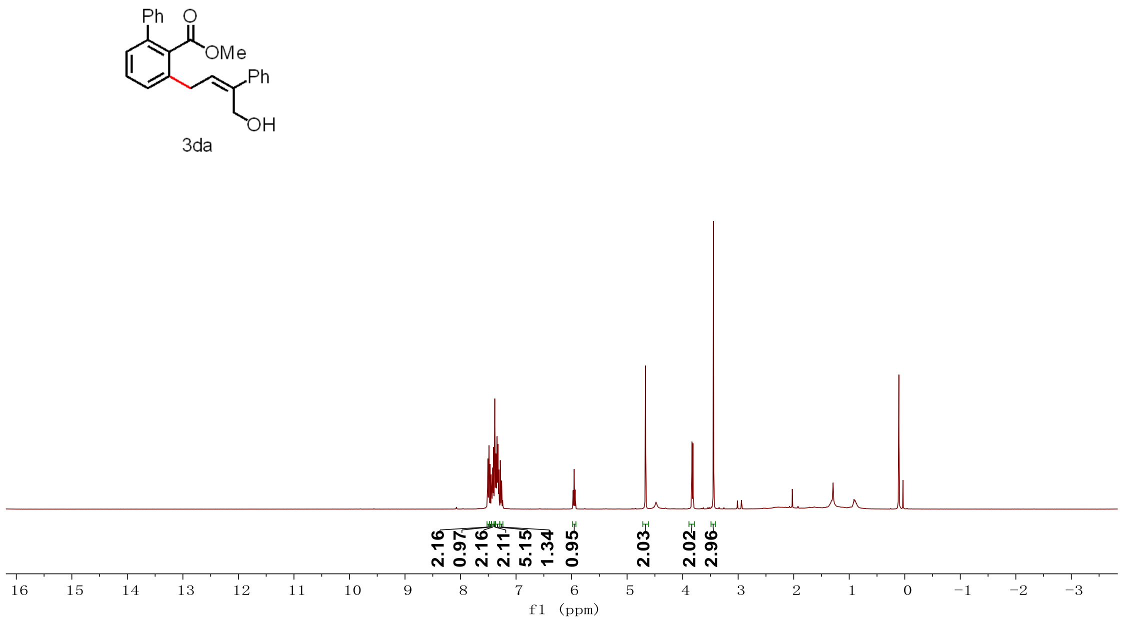

Supplementary Figure 17 ^{1}H NMR (400 MHz, $CDCl_3$) spectrum of compound **3da**

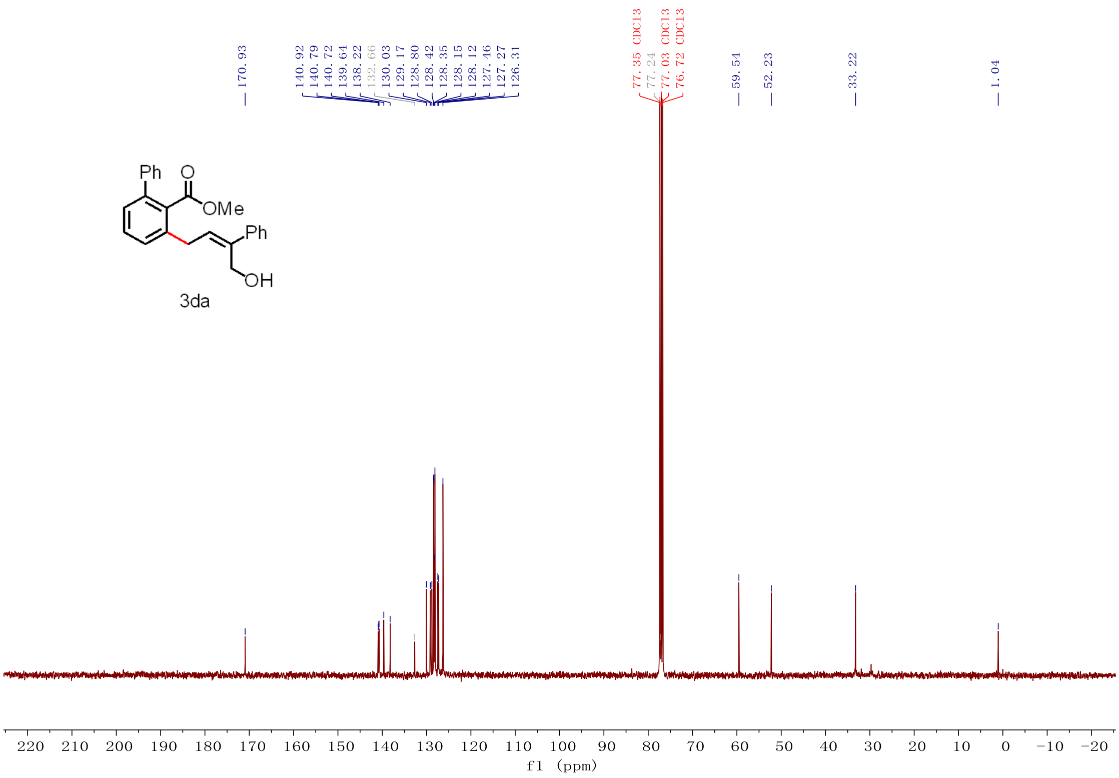

Supplementary Figure 18 ^{13}C NMR (101 MHz, $CDCl_3$) spectrum of compound **3da**

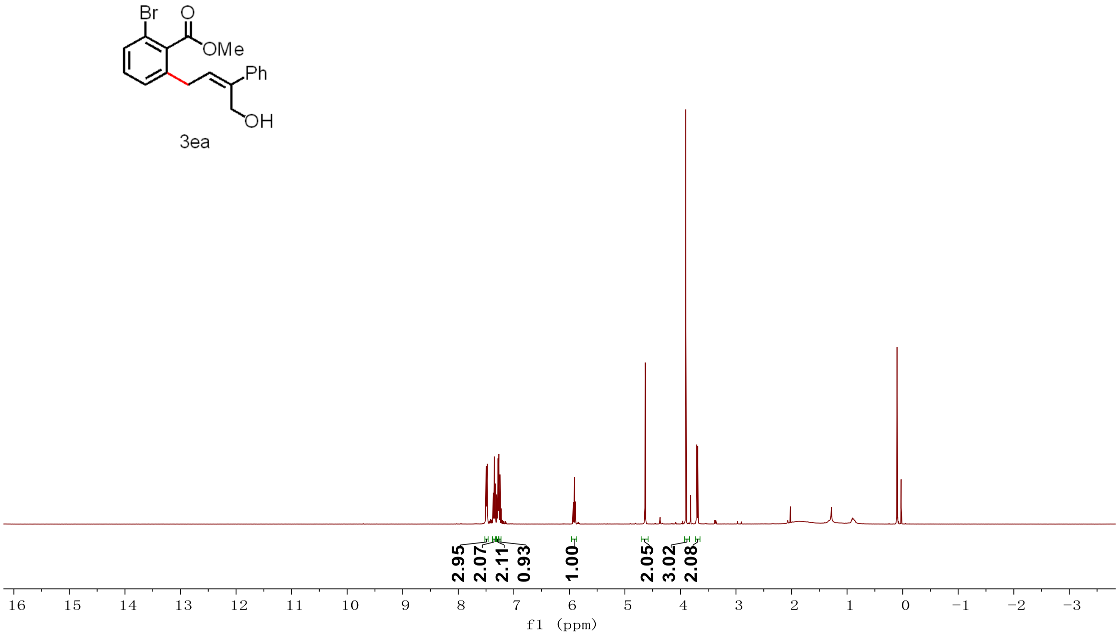

Supplementary Figure 19 ^{1}H NMR (400 MHz, $CDCl_3$) spectrum of compound **3ea**

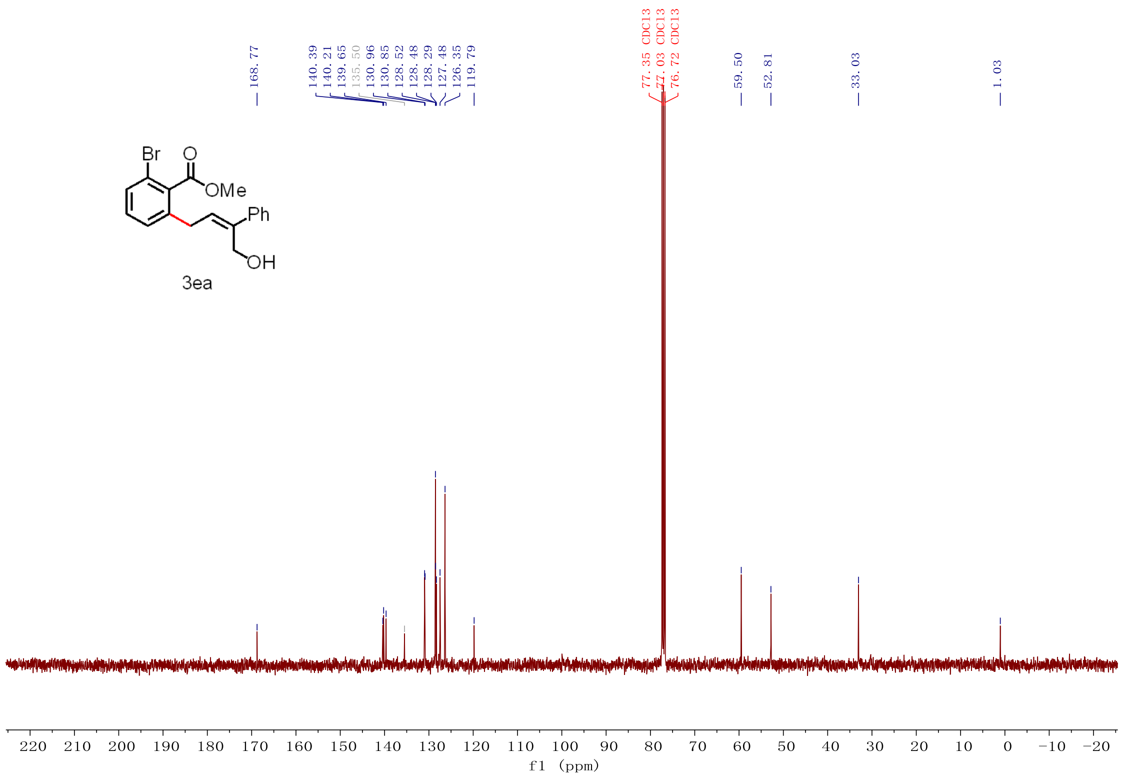

Supplementary Figure 20 ^{13}C NMR (101 MHz, $CDCl_3$) spectrum of compound **3ea**

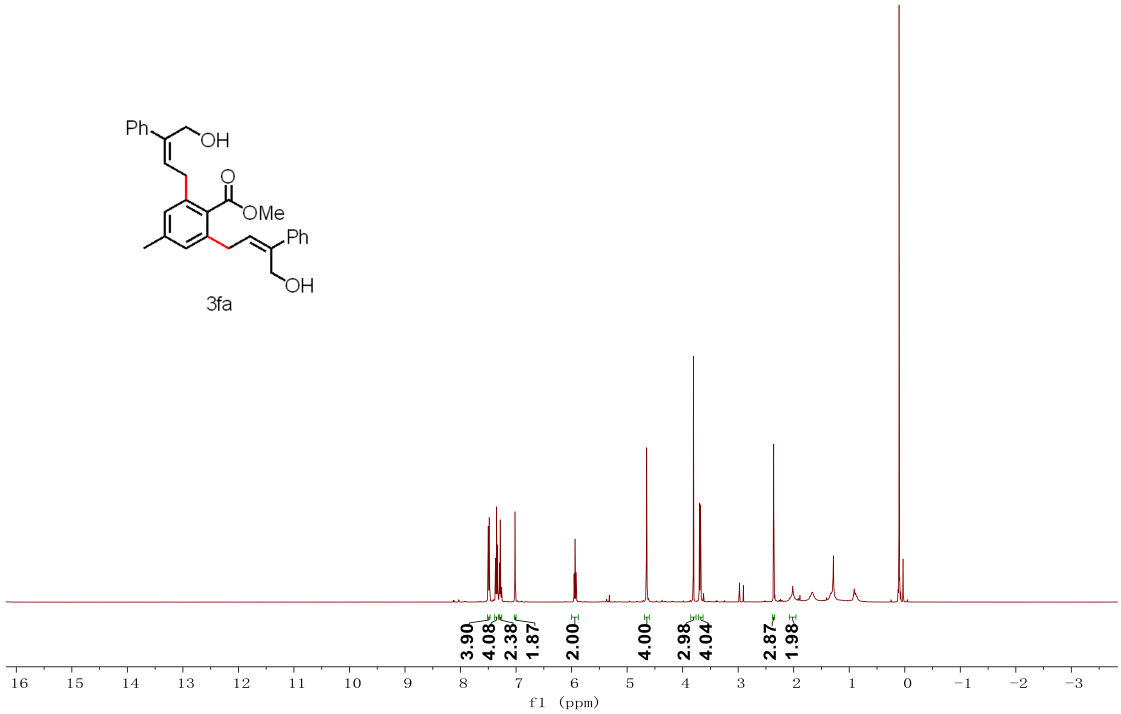

Supplementary Figure 21 ^{1}H NMR (400 MHz, $CDCl_3$) spectrum of compound **3fa**

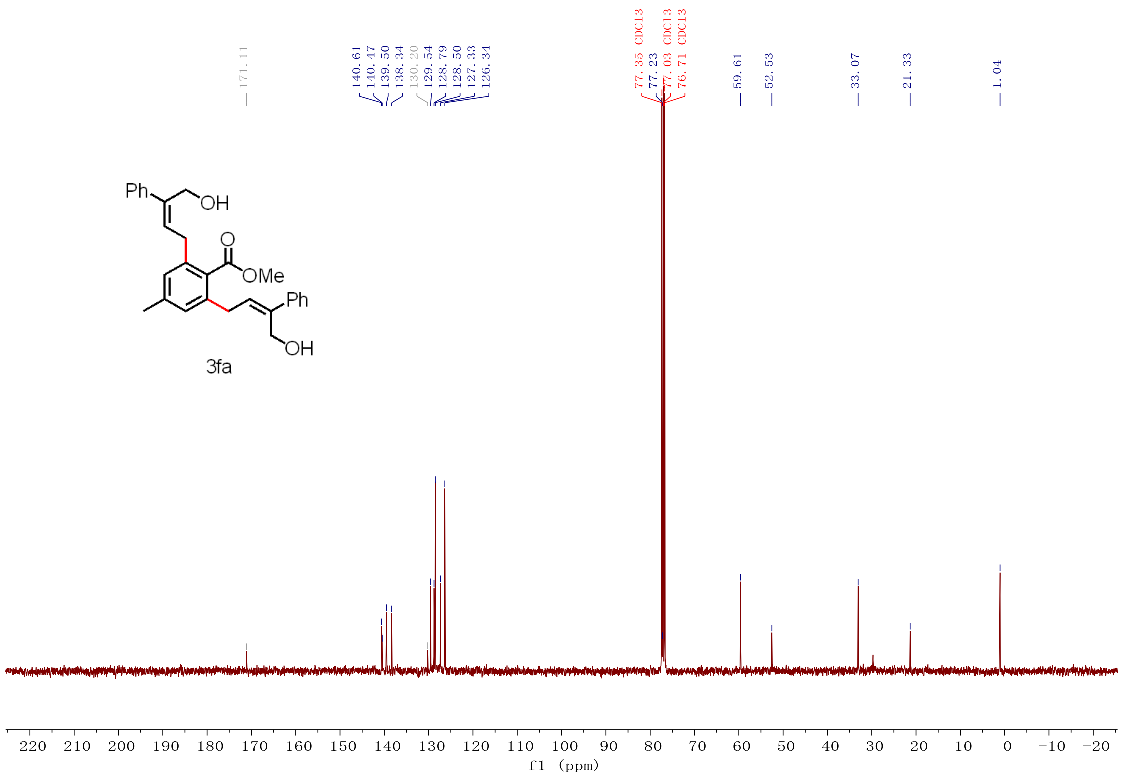

Supplementary Figure 22 ^{13}C NMR (101 MHz, $CDCl_3$) spectrum of compound **3fa**

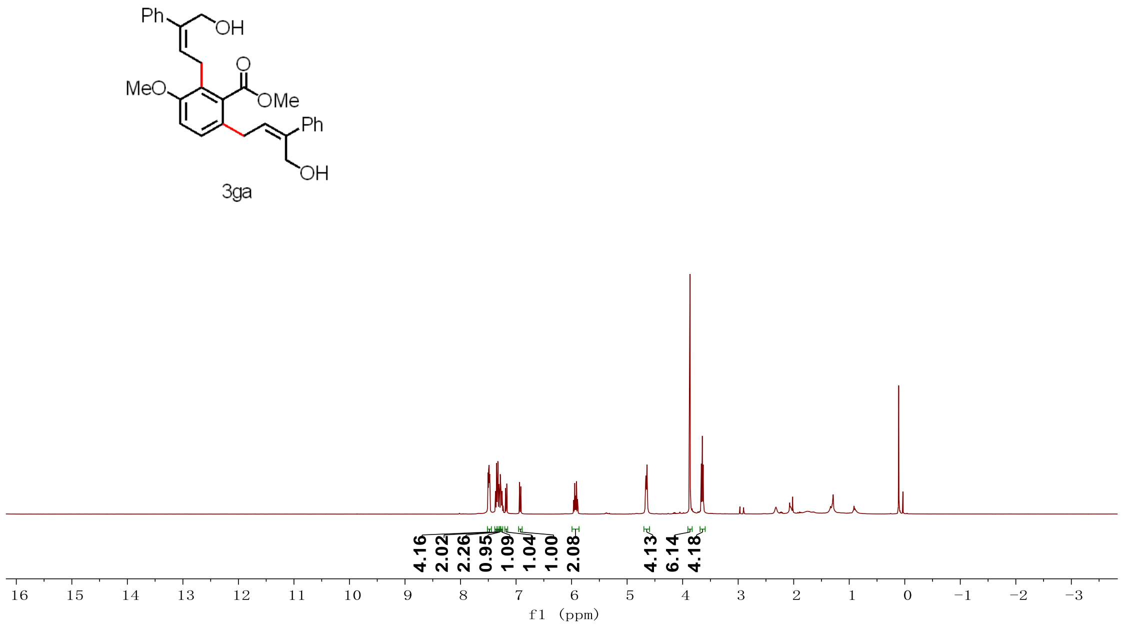

Supplementary Figure 23 ^{1}H NMR (400 MHz, $CDCl_3$) spectrum of compound **3ga**

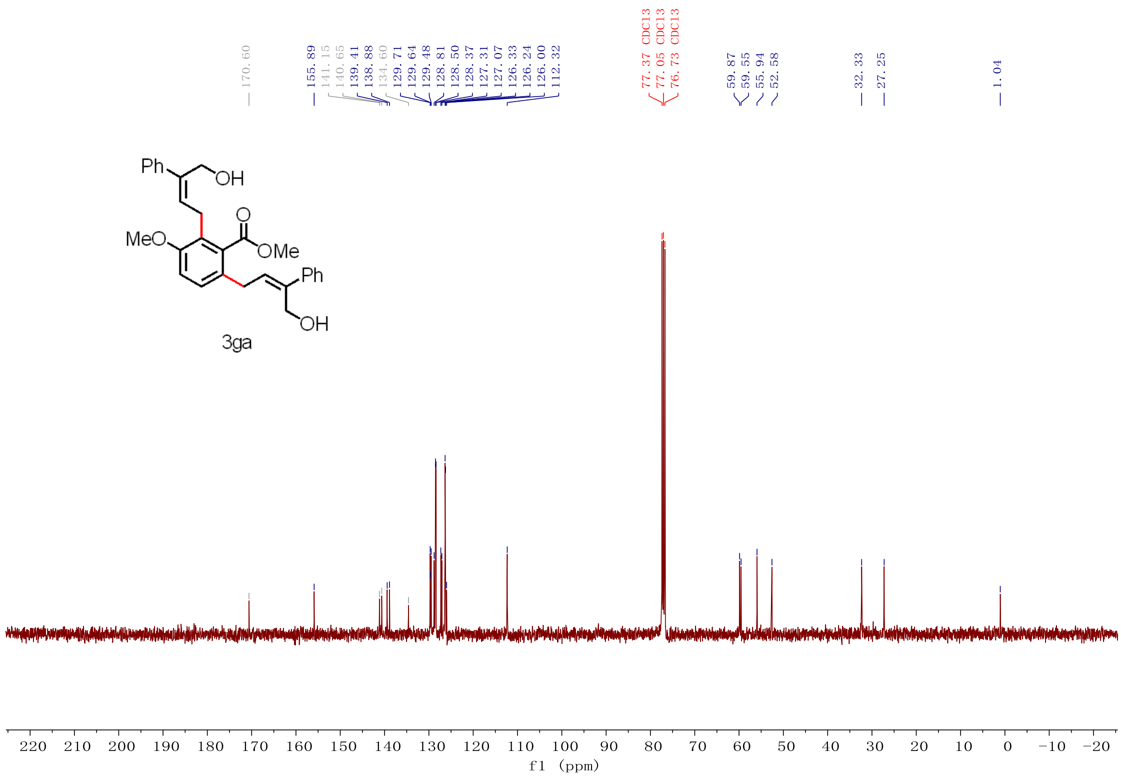

Supplementary Figure 24 ^{13}C NMR (101 MHz, $CDCl_3$) spectrum of compound **3ga**

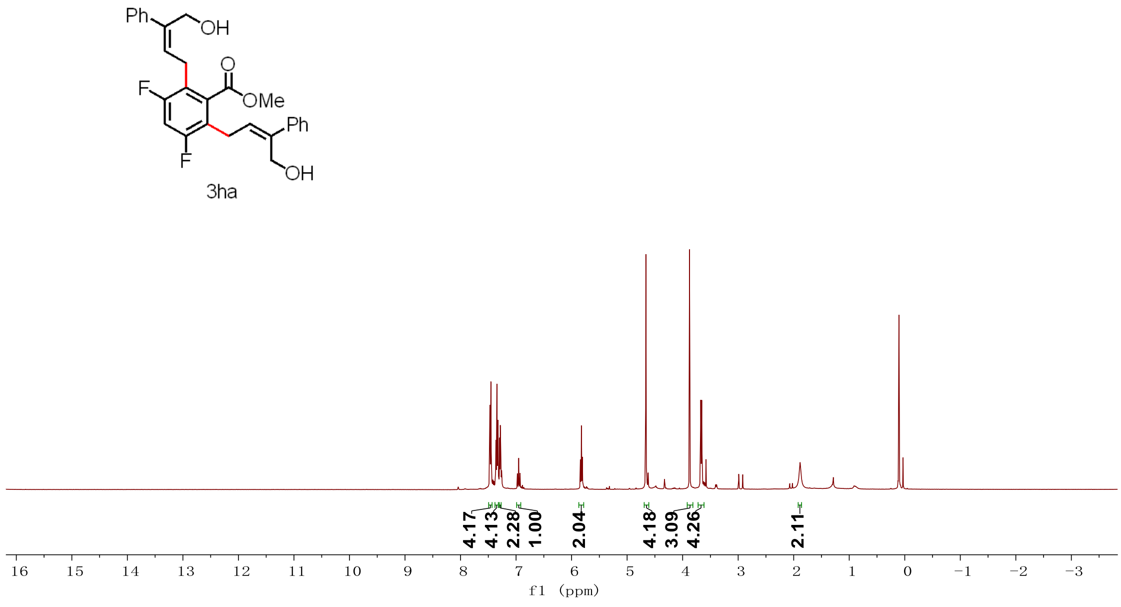

Supplementary Figure 25 ^{1}H NMR (400 MHz, $CDCl_3$) spectrum of compound **3ha**

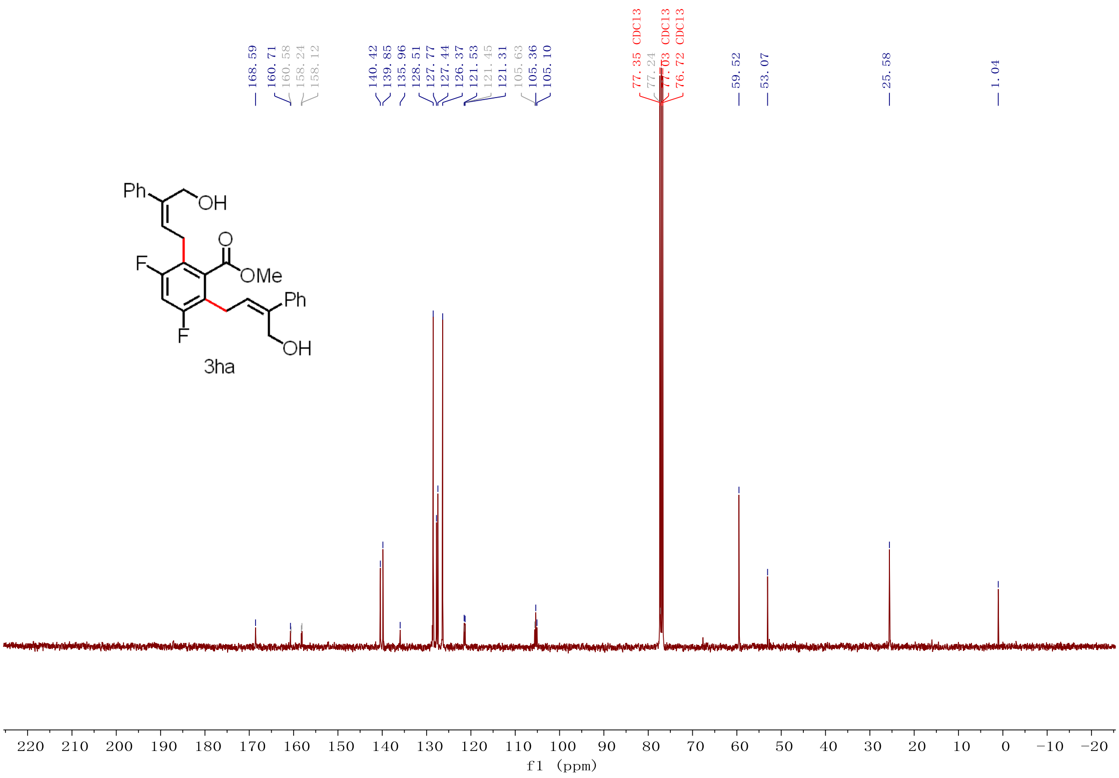

Supplementary Figure 26 ^{13}C NMR (101 MHz, $CDCl_3$) spectrum of compound **3ha**

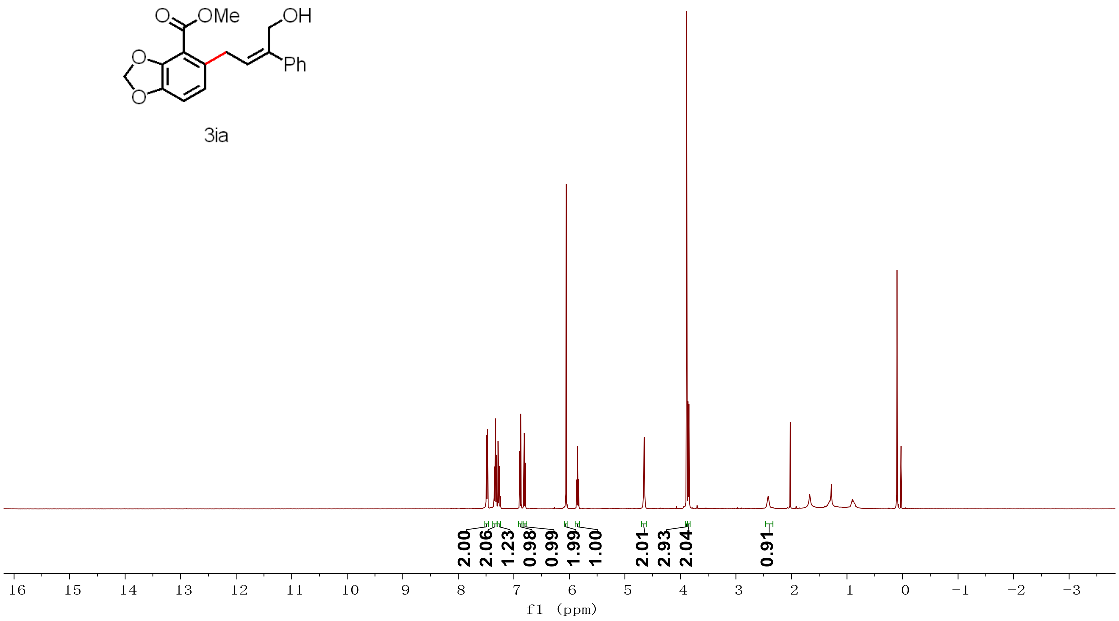

Supplementary Figure 27 ^{1}H NMR (400 MHz, $CDCl_3$) spectrum of compound **3ia**

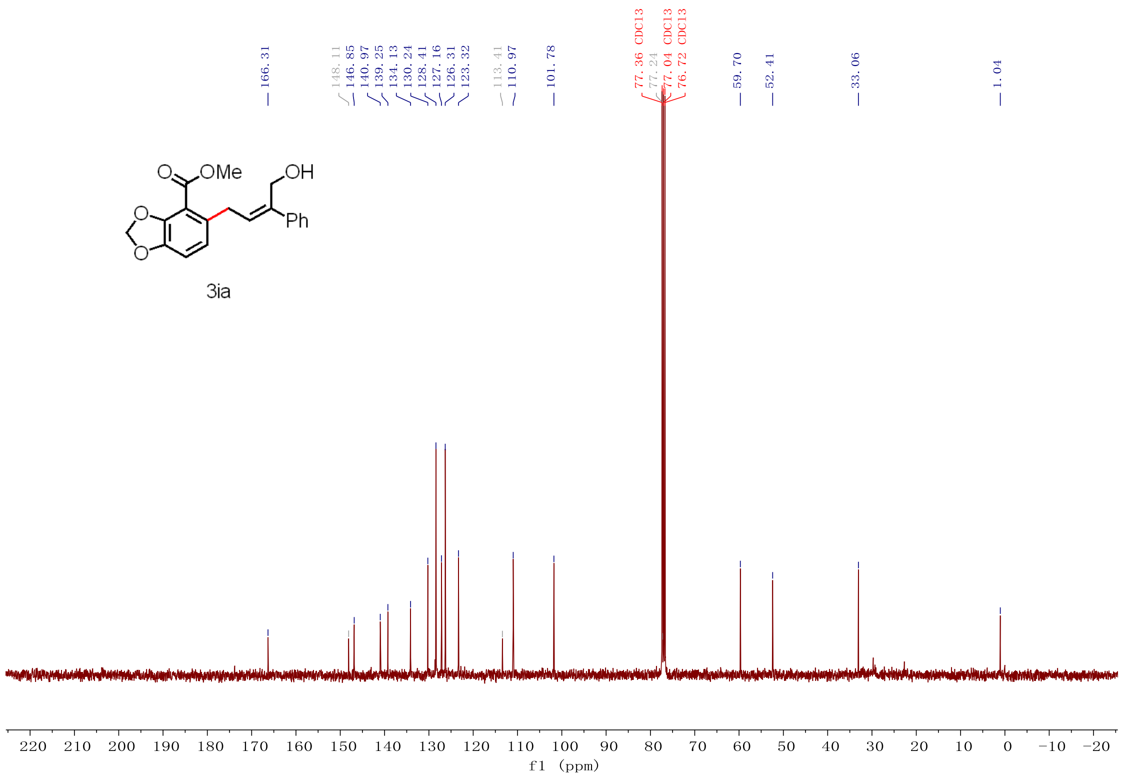

Supplementary Figure 28 ^{13}C NMR (101 MHz, $CDCl_3$) spectrum of compound **3ia**

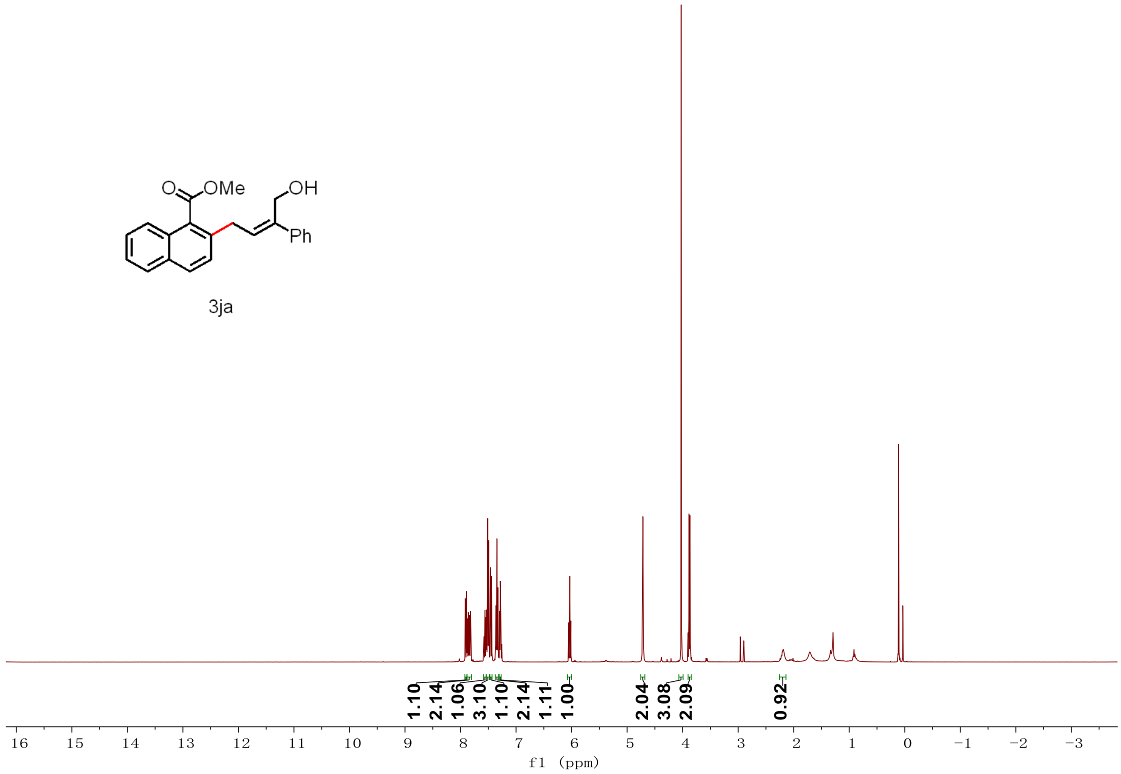

Supplementary Figure 29 ^{1}H NMR (400 MHz, $CDCl_3$) spectrum of compound **3ja**

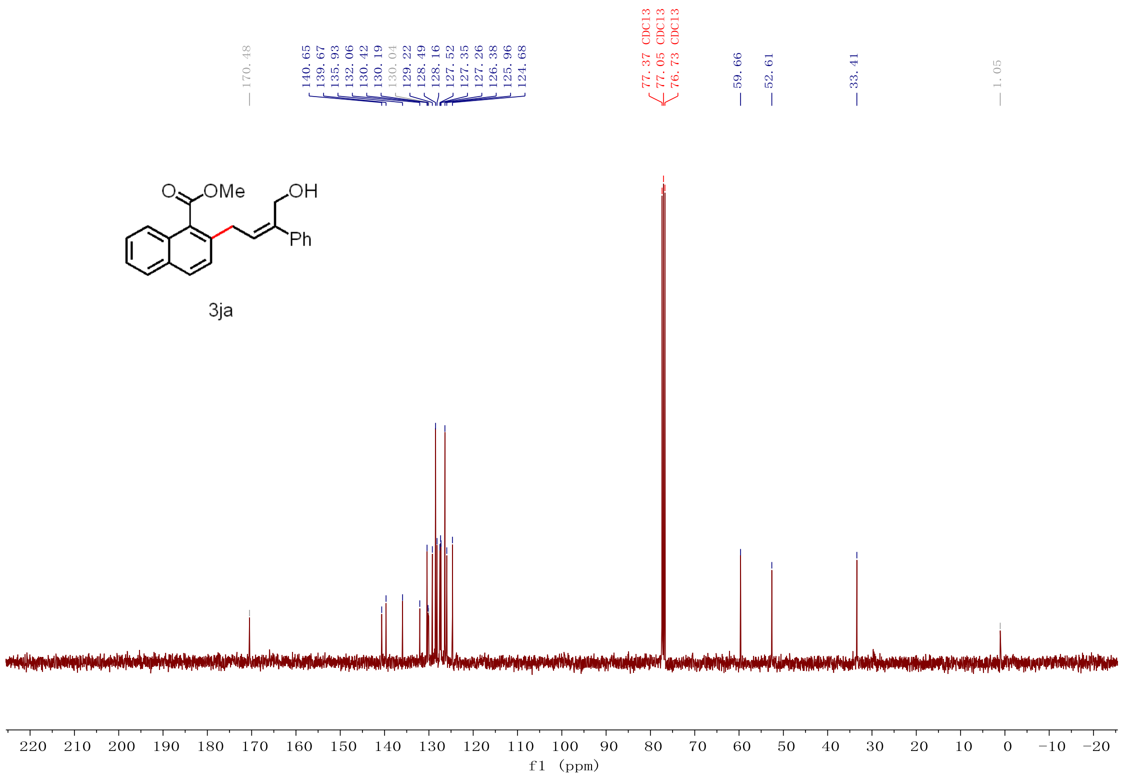

Supplementary Figure 30 ^{13}C NMR (101 MHz, $CDCl_3$) spectrum of compound **3ja**

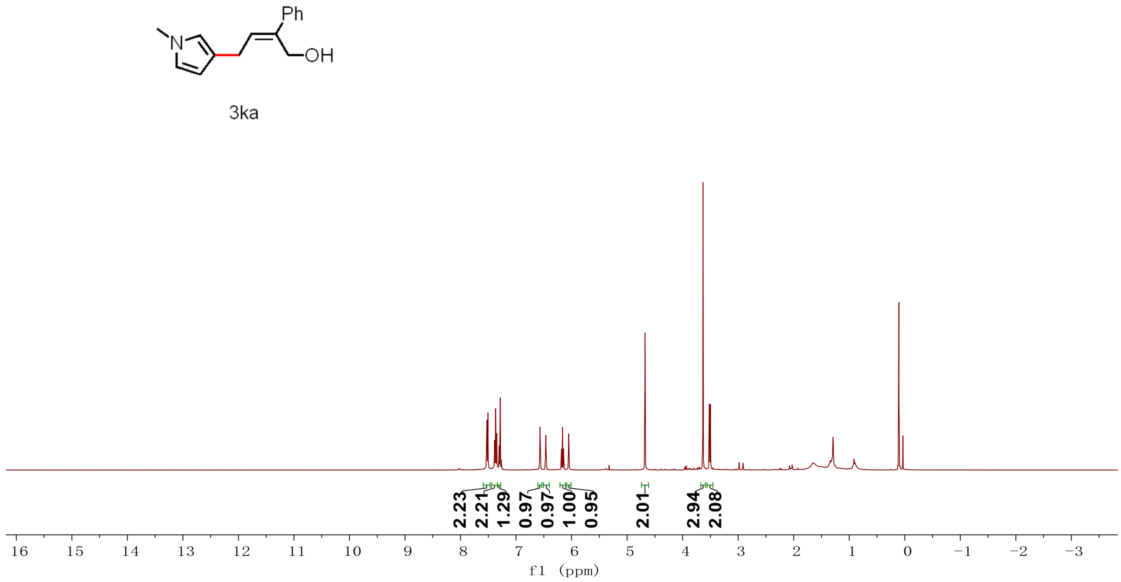

Supplementary Figure 31 ^{1}H NMR (400 MHz, $CDCl_3$) spectrum of compound **3ka**

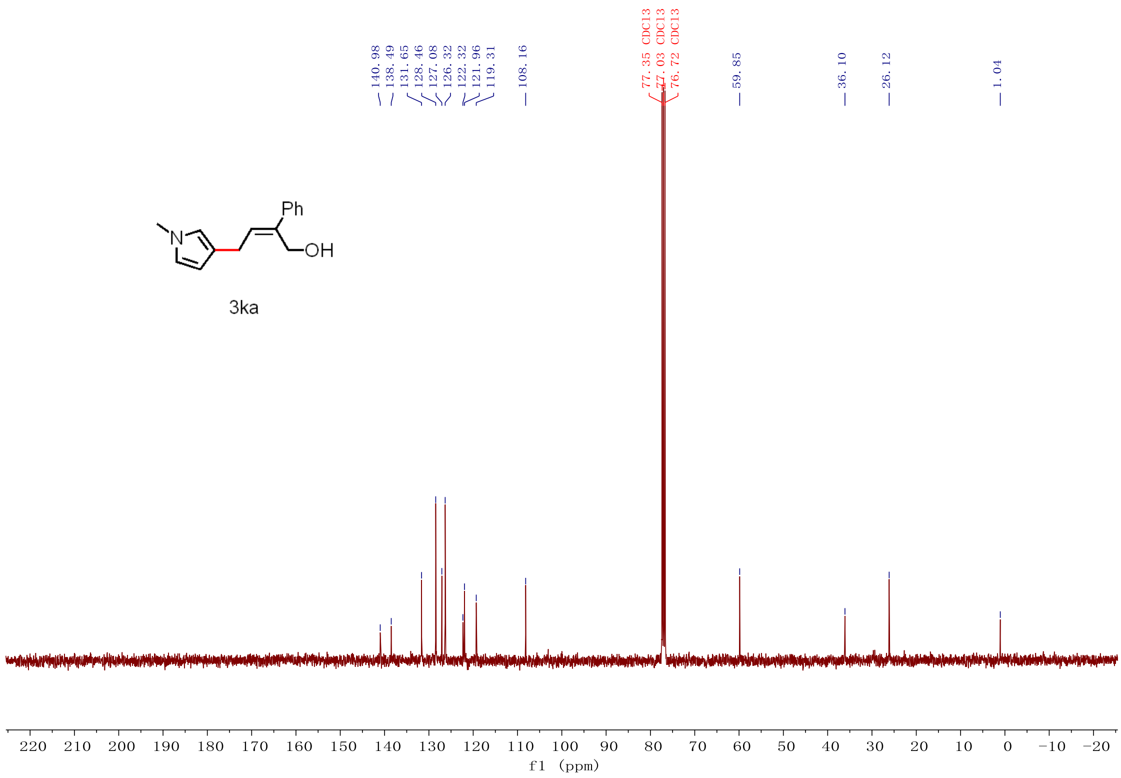

3ka

Supplementary Figure 32 ^{13}C NMR (101 MHz, $CDCl_3$) spectrum of compound **3ka**

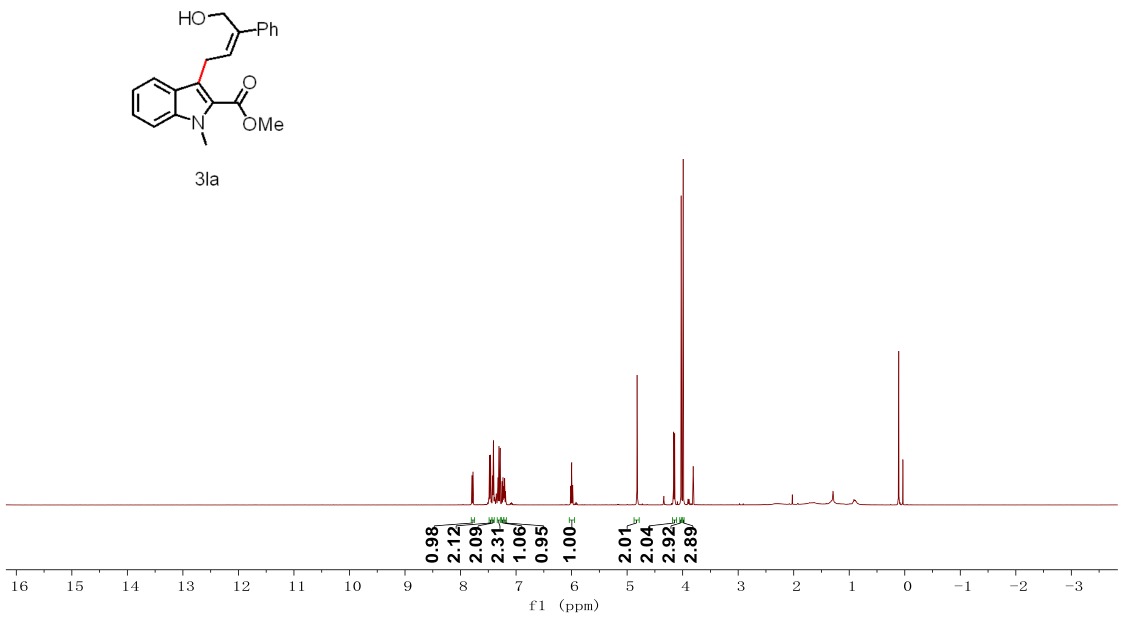

3la

Supplementary Figure 33 ^{1}H NMR (400 MHz, $CDCl_3$) spectrum of compound **3la**

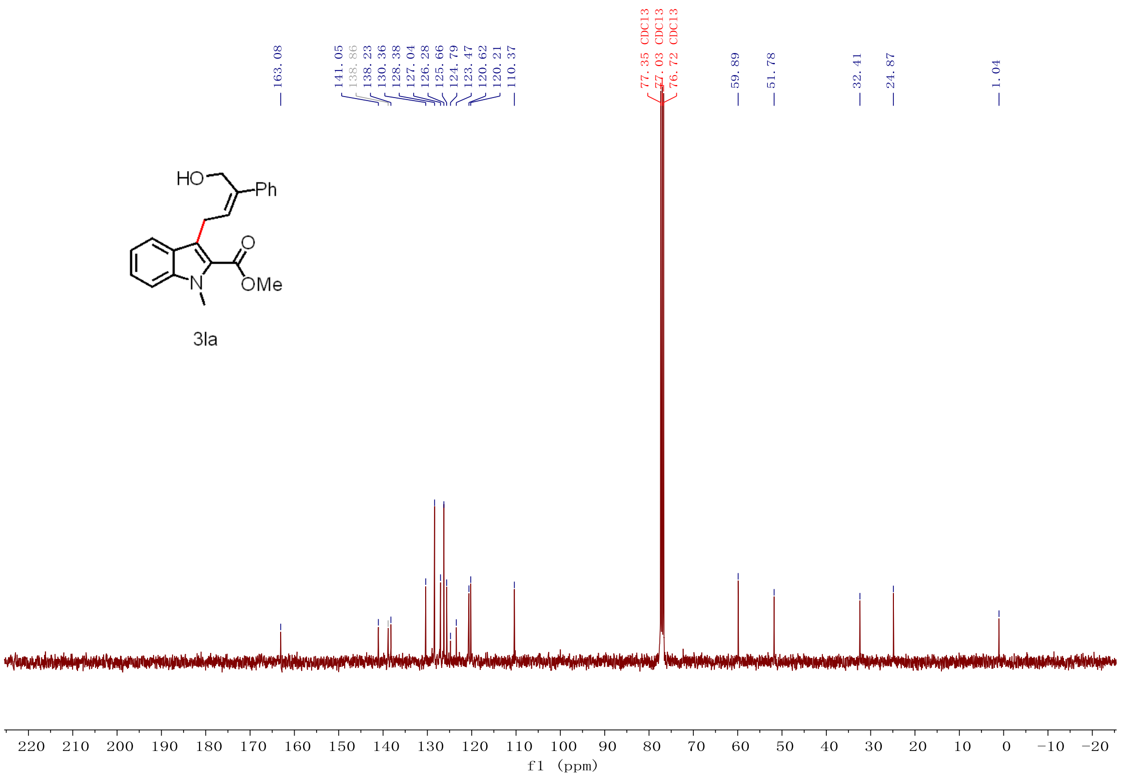

Supplementary Figure 34 ^{13}C NMR (101 MHz, $CDCl_3$) spectrum of compound **3la**

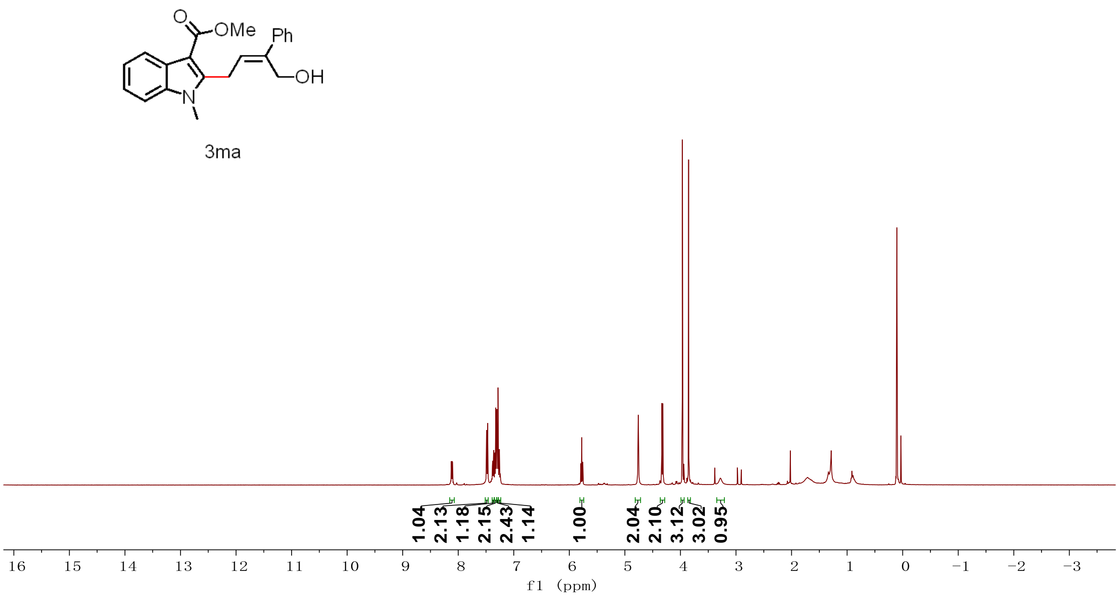

Supplementary Figure 35 ^{1}H NMR (400 MHz, $CDCl_3$) spectrum of compound **3ma**

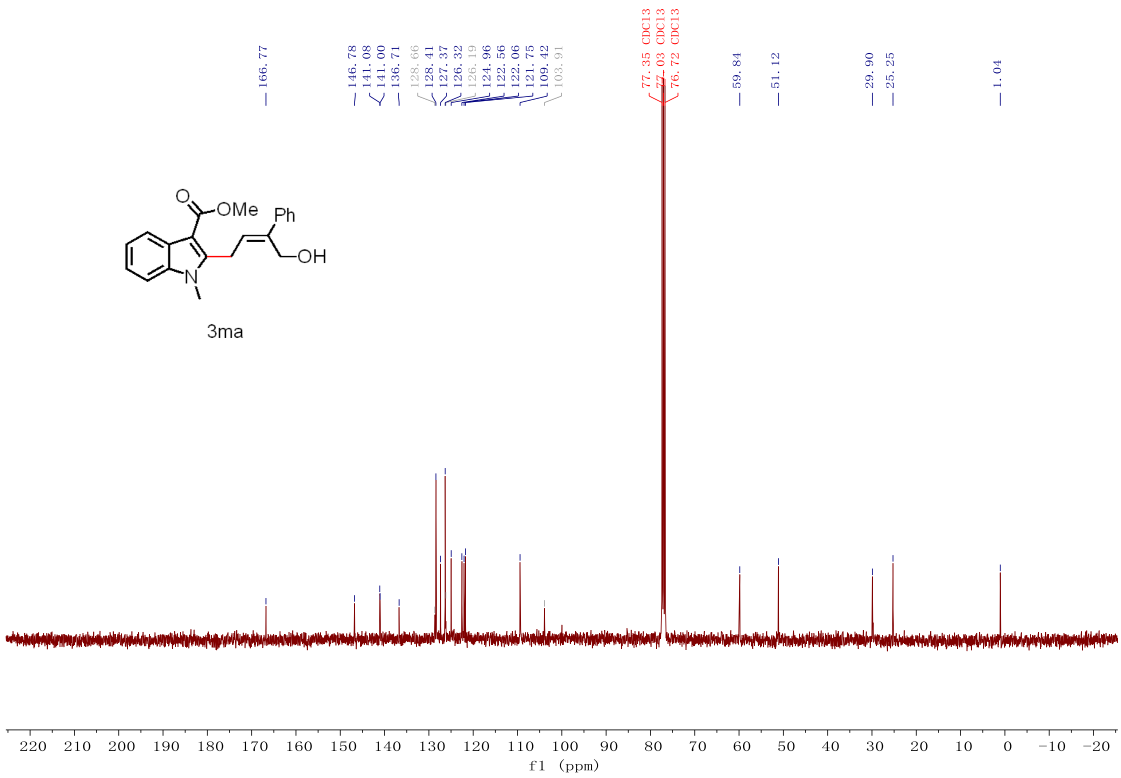

Supplementary Figure 36 ^{13}C NMR (101 MHz, $CDCl_3$) spectrum of compound **3ma**

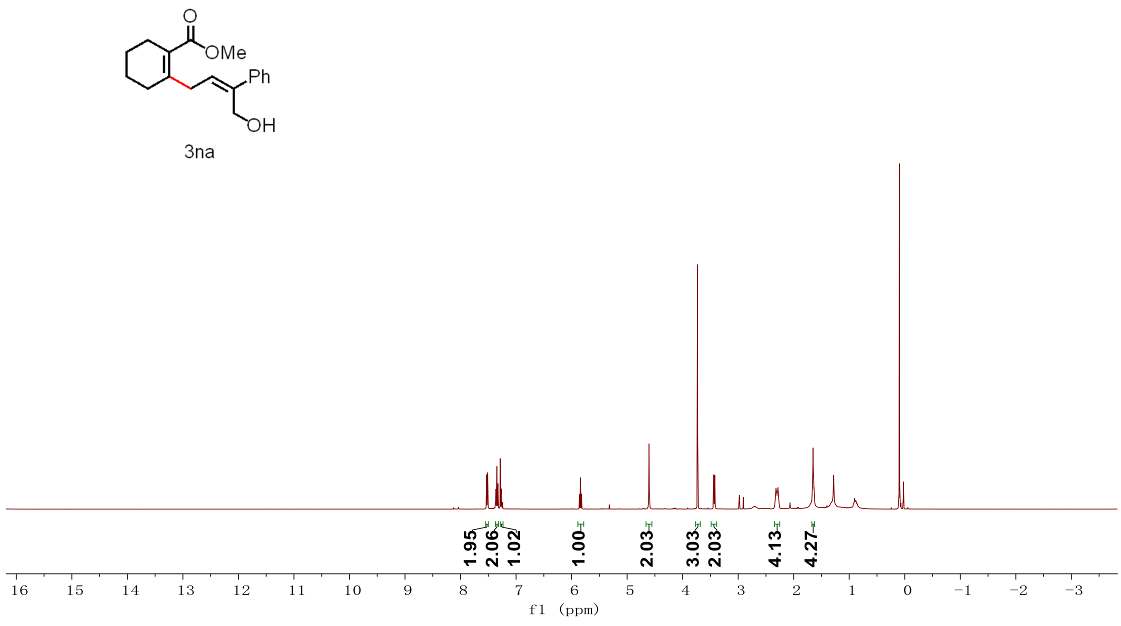

Supplementary Figure 37 ^{1}H NMR (400 MHz, $CDCl_3$) spectrum of compound **3na**

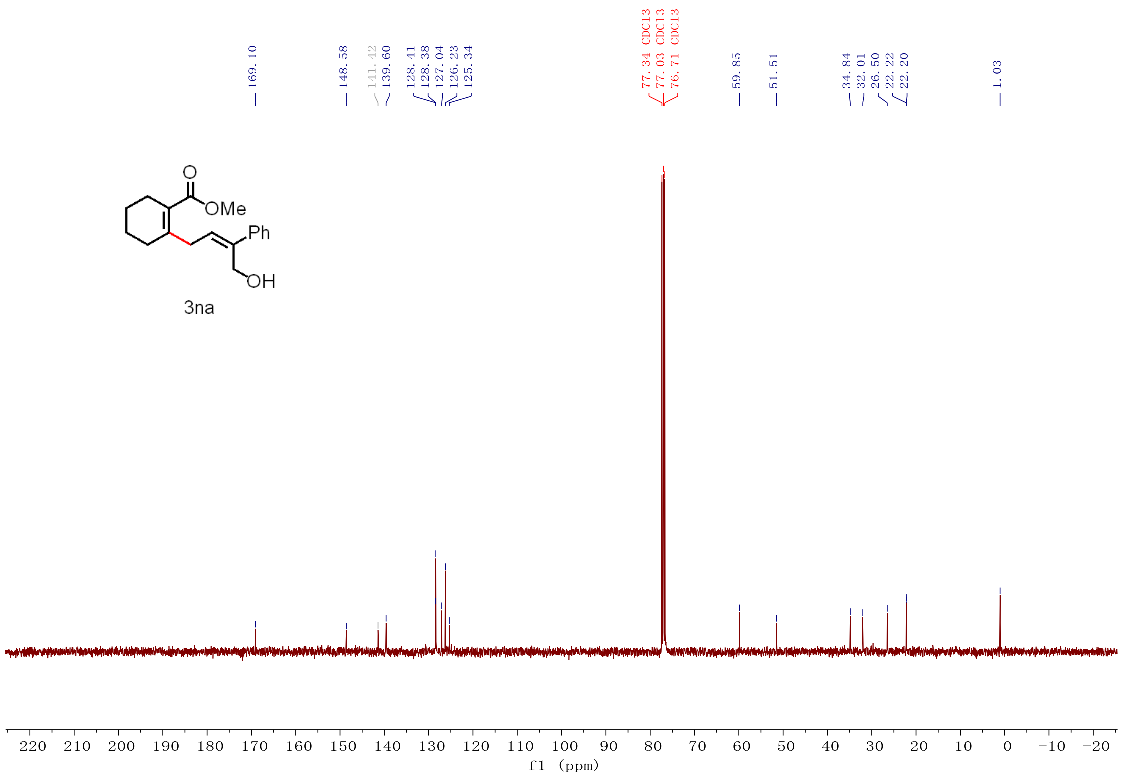

Supplementary Figure 38 ^{13}C NMR (101 MHz, $CDCl_3$) spectrum of compound **3na**

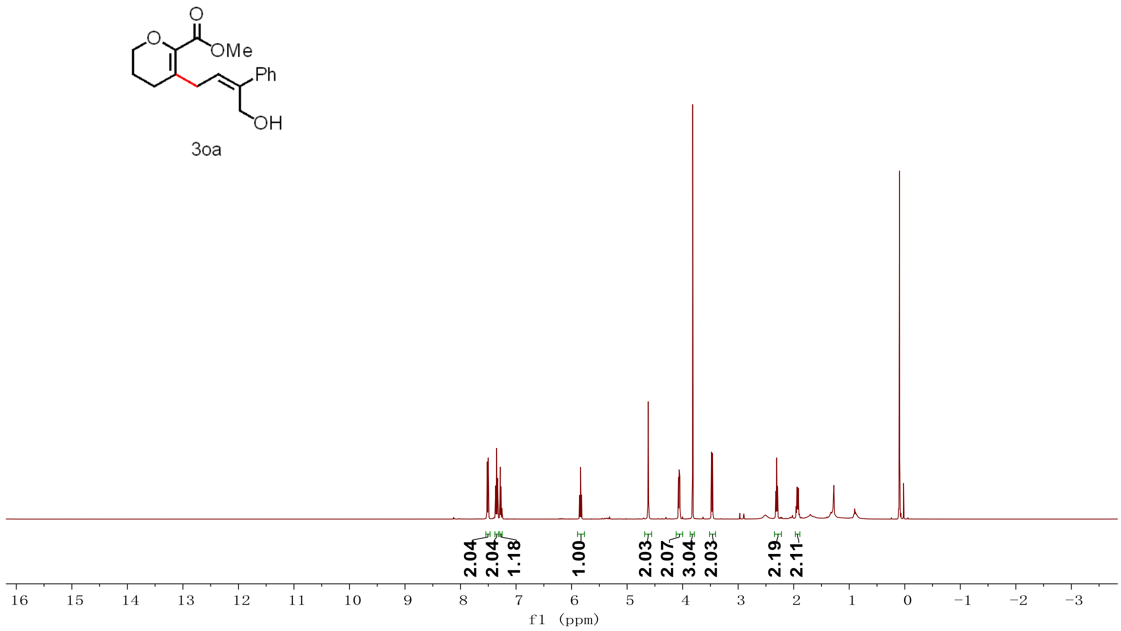

Supplementary Figure 39 ^{1}H NMR (400 MHz, $CDCl_3$) spectrum of compound **3oa**

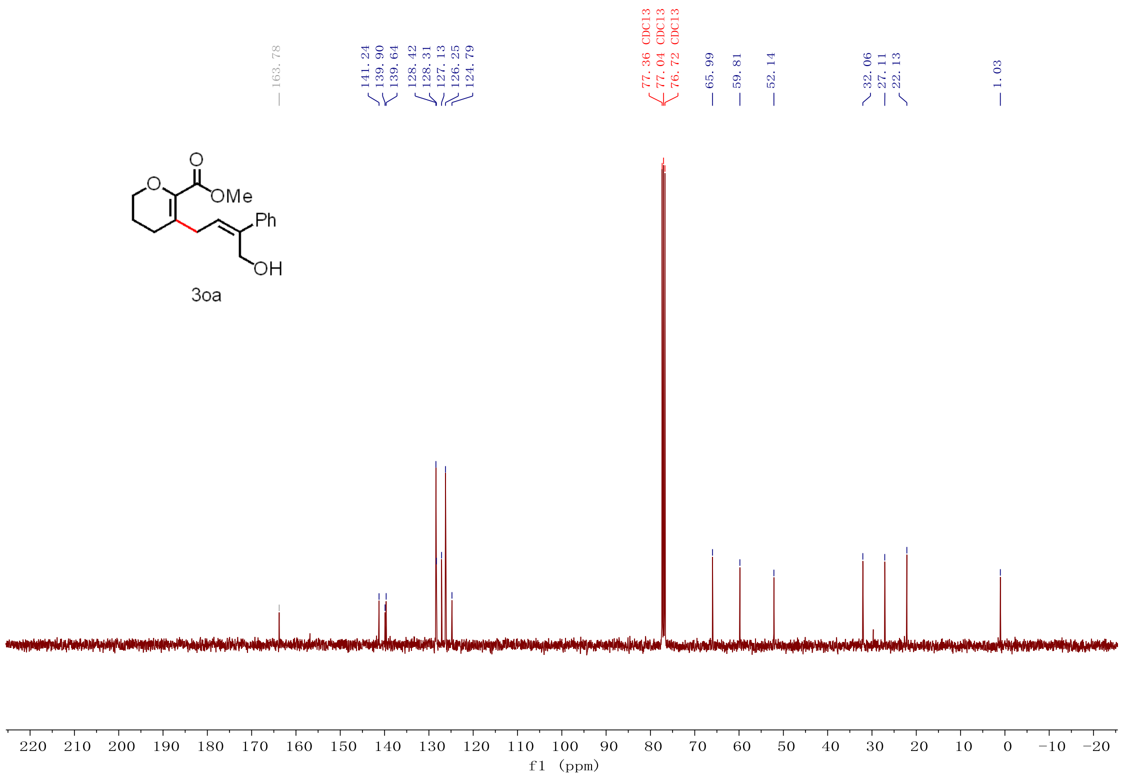

Supplementary Figure 40 ^{13}C NMR (101 MHz, $CDCl_3$) spectrum of compound **3oa**

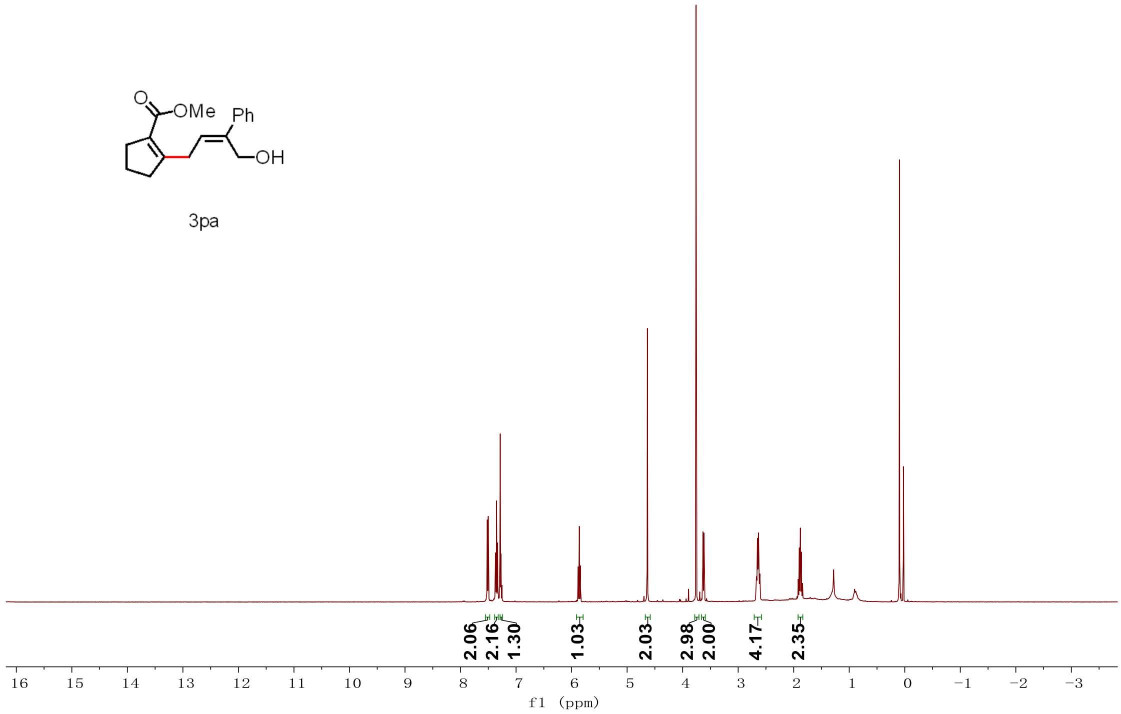

Supplementary Figure 41 ^{1}H NMR (400 MHz, $CDCl_3$) spectrum of compound **3pa**

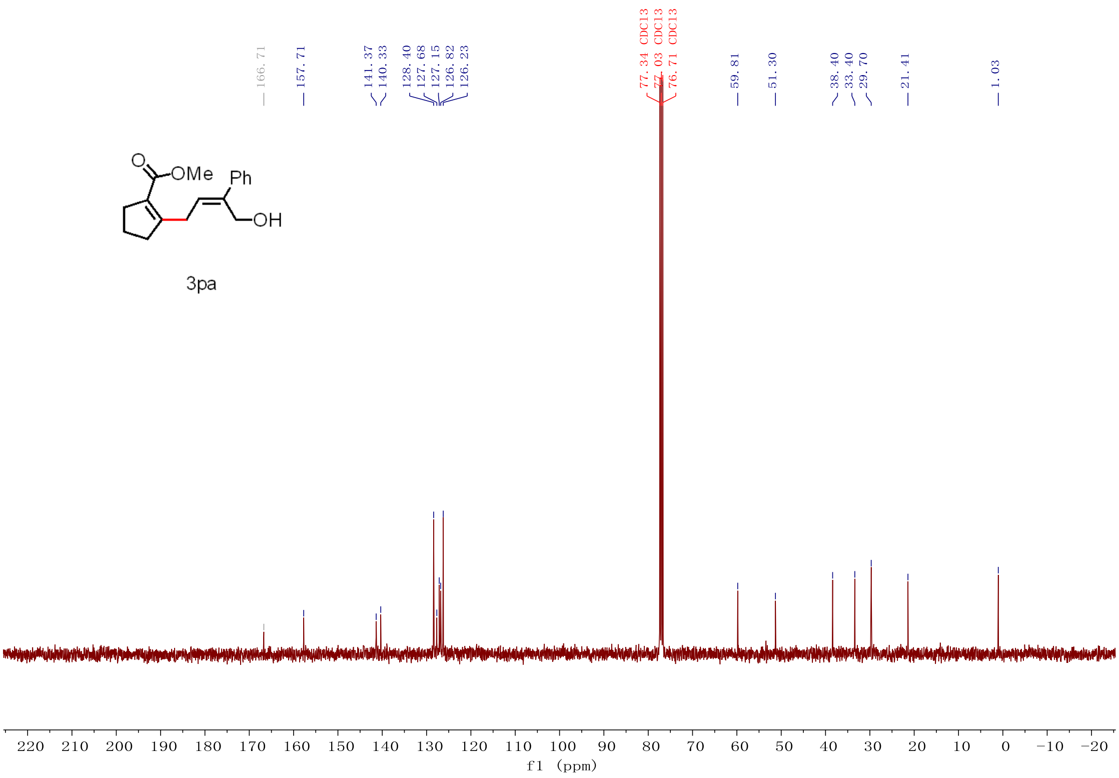

Supplementary Figure 42 ^{13}C NMR (101 MHz, $CDCl_3$) spectrum of compound **3pa**

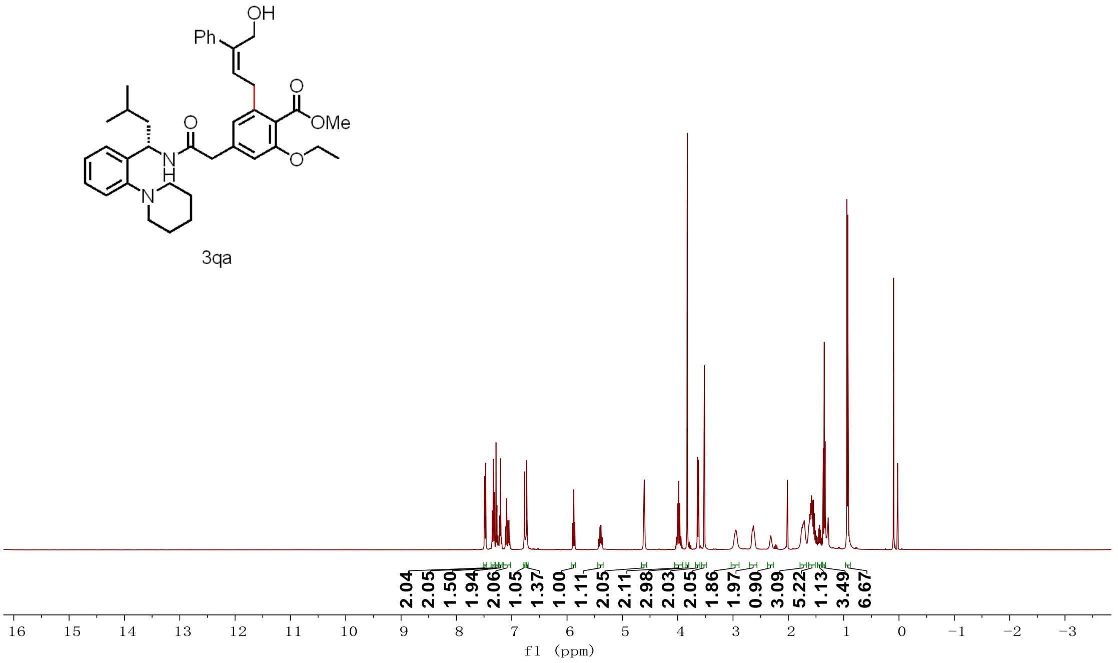

Supplementary Figure 43 ^{1}H NMR (400 MHz, $CDCl_3$) spectrum of compound **3qa**

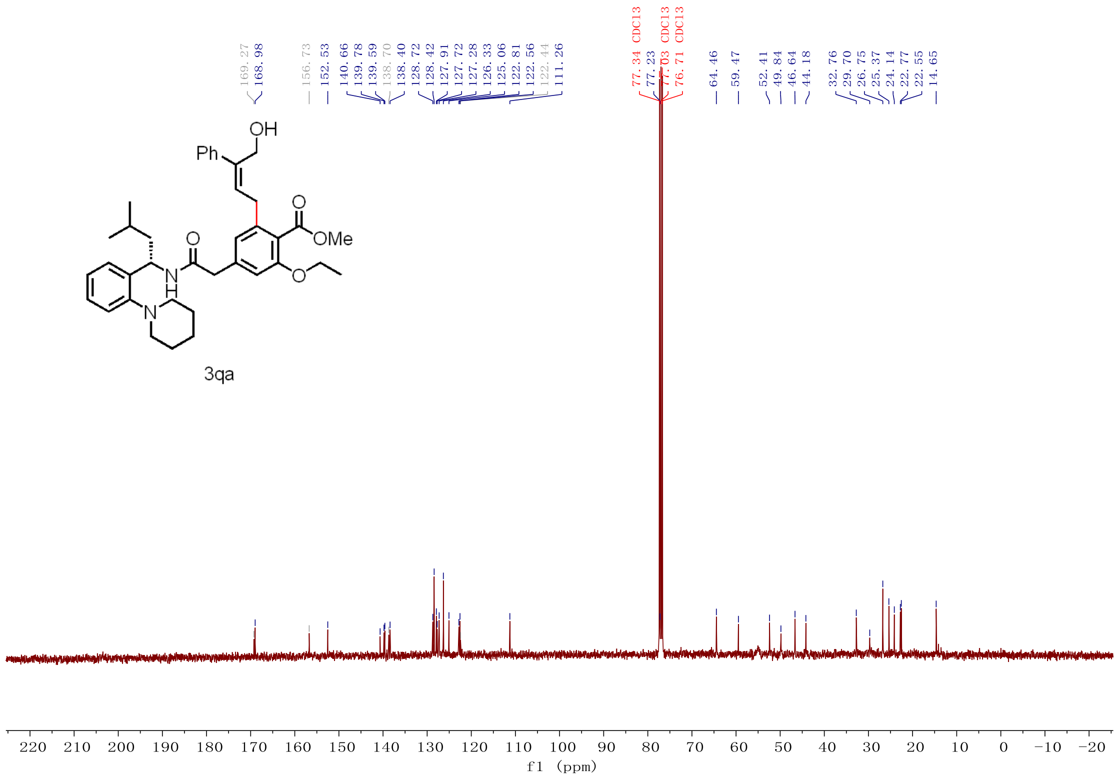

Supplementary Figure 44 ^{13}C NMR (101 MHz, $CDCl_3$) spectrum of compound **3qa**

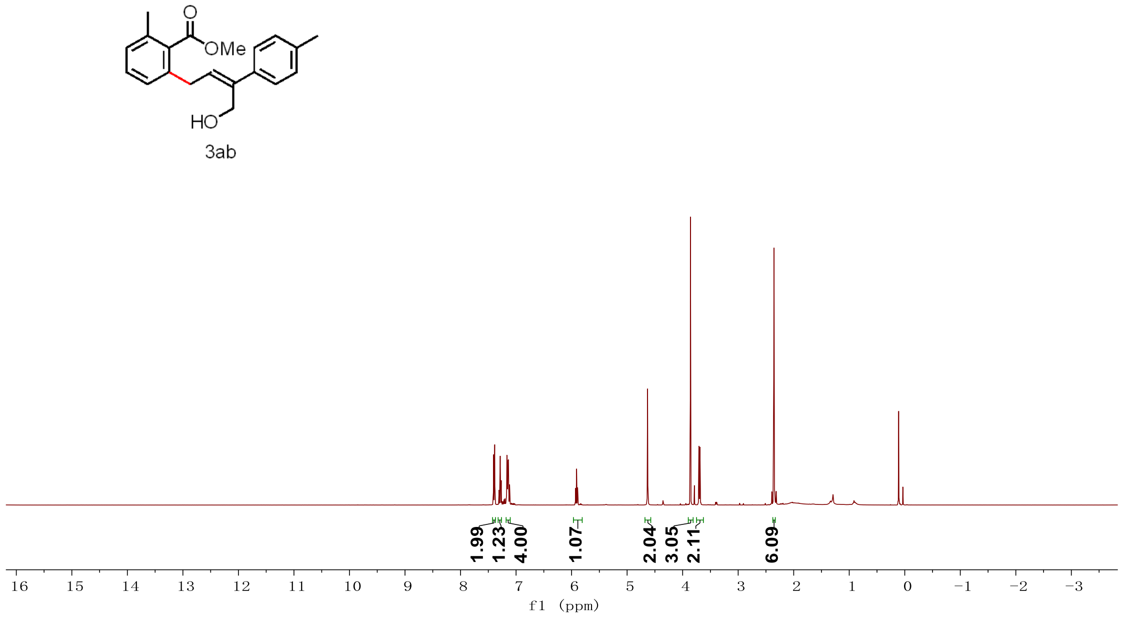

Supplementary Figure 45 ^{1}H NMR (400 MHz, $CDCl_3$) spectrum of compound **3ab**

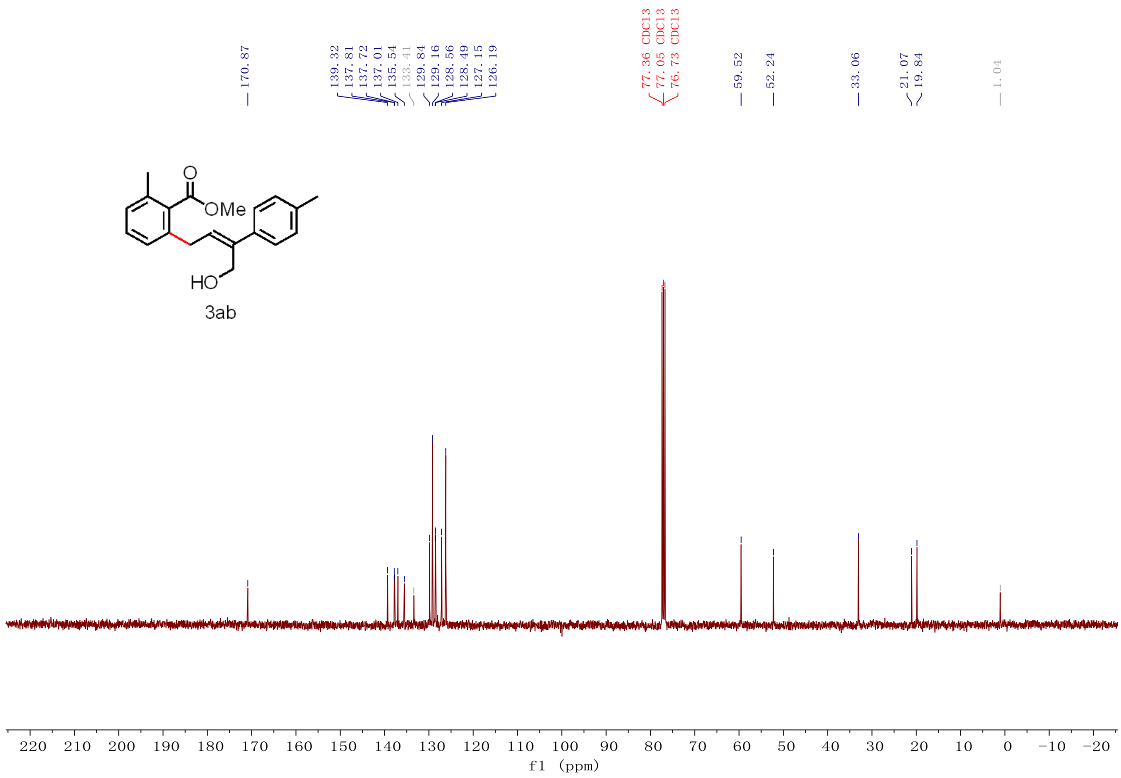

Supplementary Figure 46 ^{13}C NMR (101 MHz, $CDCl_3$) spectrum of compound **3ab**

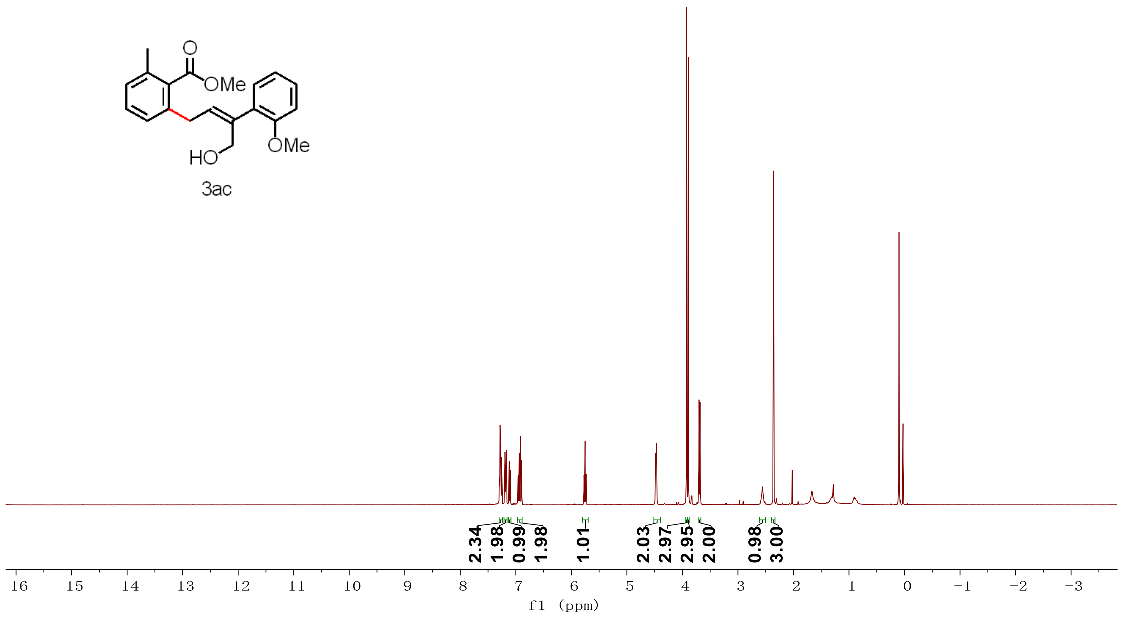

Supplementary Figure 47 ^{1}H NMR (400 MHz, $CDCl_3$) spectrum of compound **3ac**

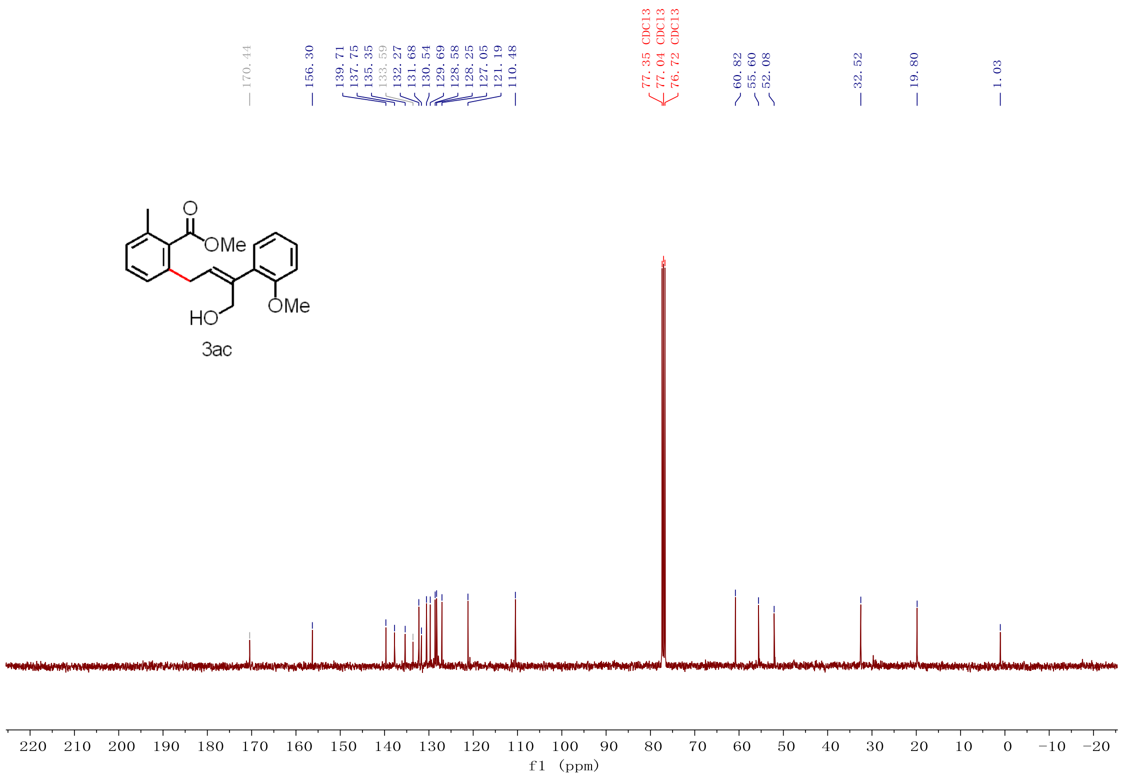

Supplementary Figure 48 ^{13}C NMR (101 MHz, $CDCl_3$) spectrum of compound **3ac**

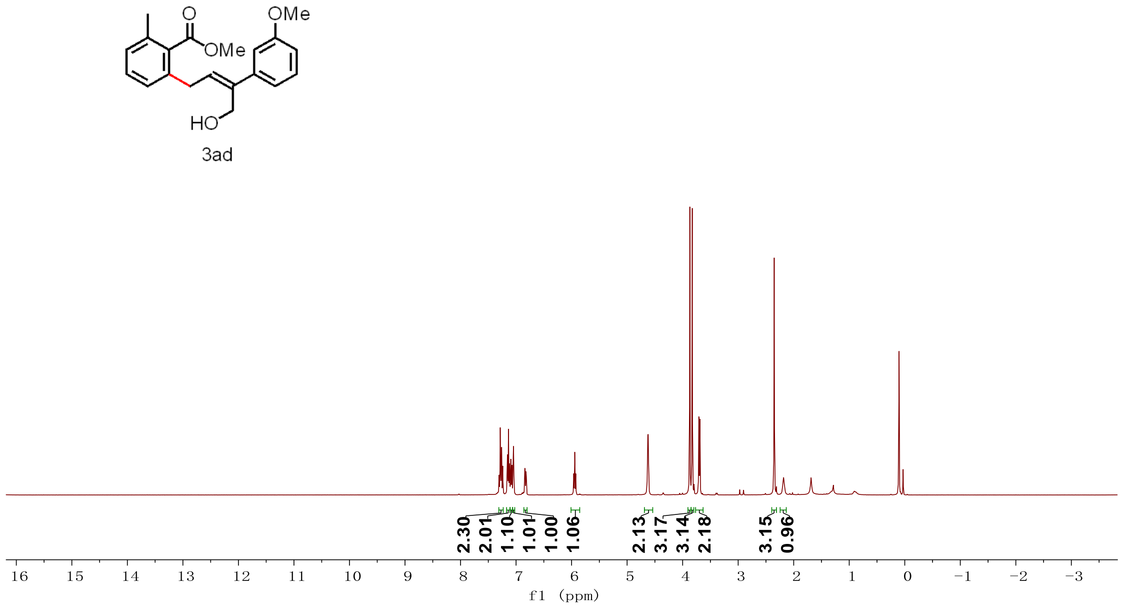

Supplementary Figure 49 ^{1}H NMR (400 MHz, $CDCl_3$) spectrum of compound **3ad**

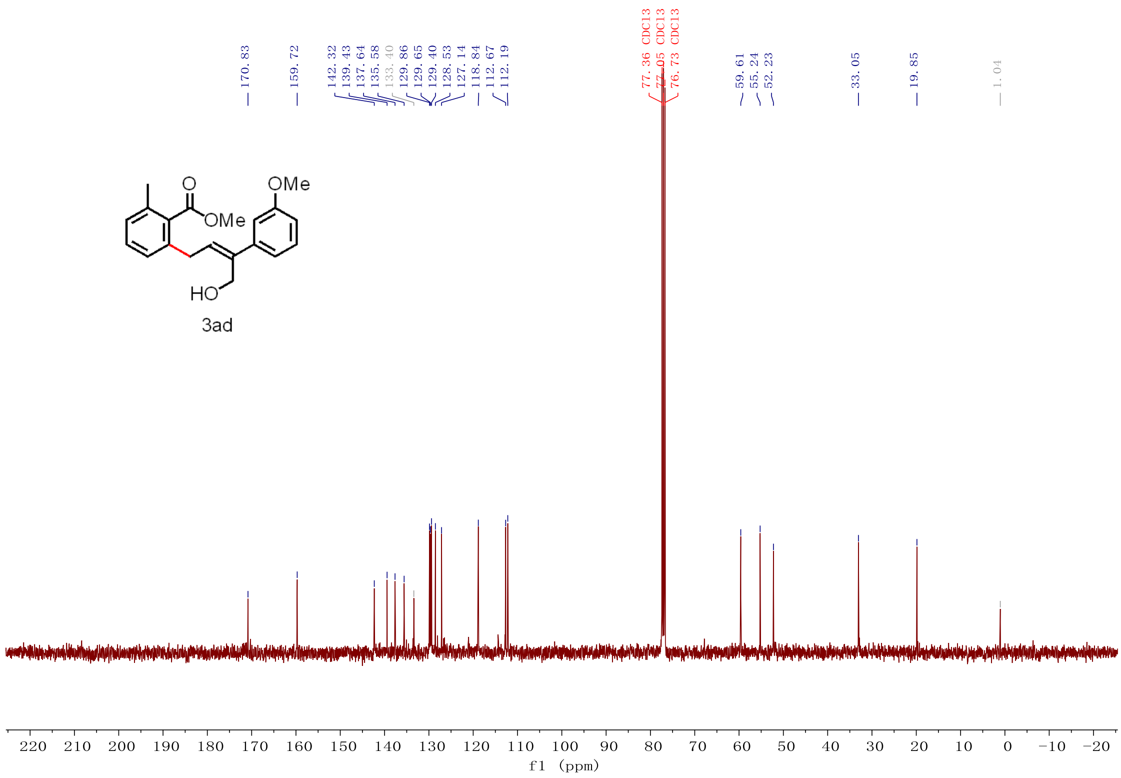

Supplementary Figure 50 ^{13}C NMR (101 MHz, $CDCl_3$) spectrum of compound **3ad**

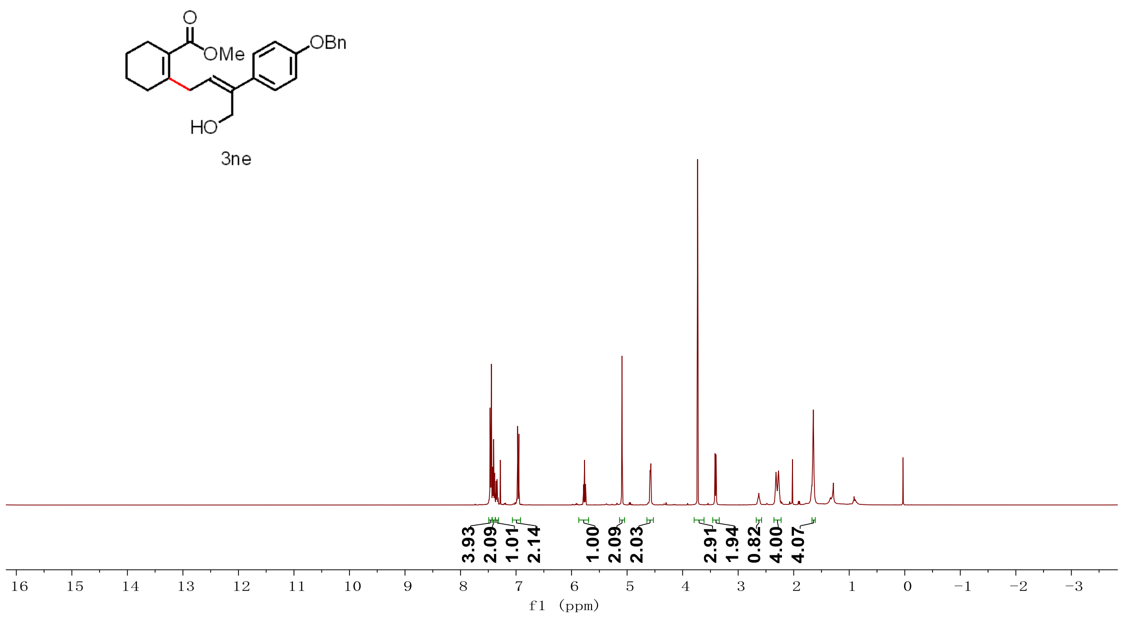

Supplementary Figure 51 ^{1}H NMR (400 MHz, $CDCl_3$) spectrum of compound **3ne**

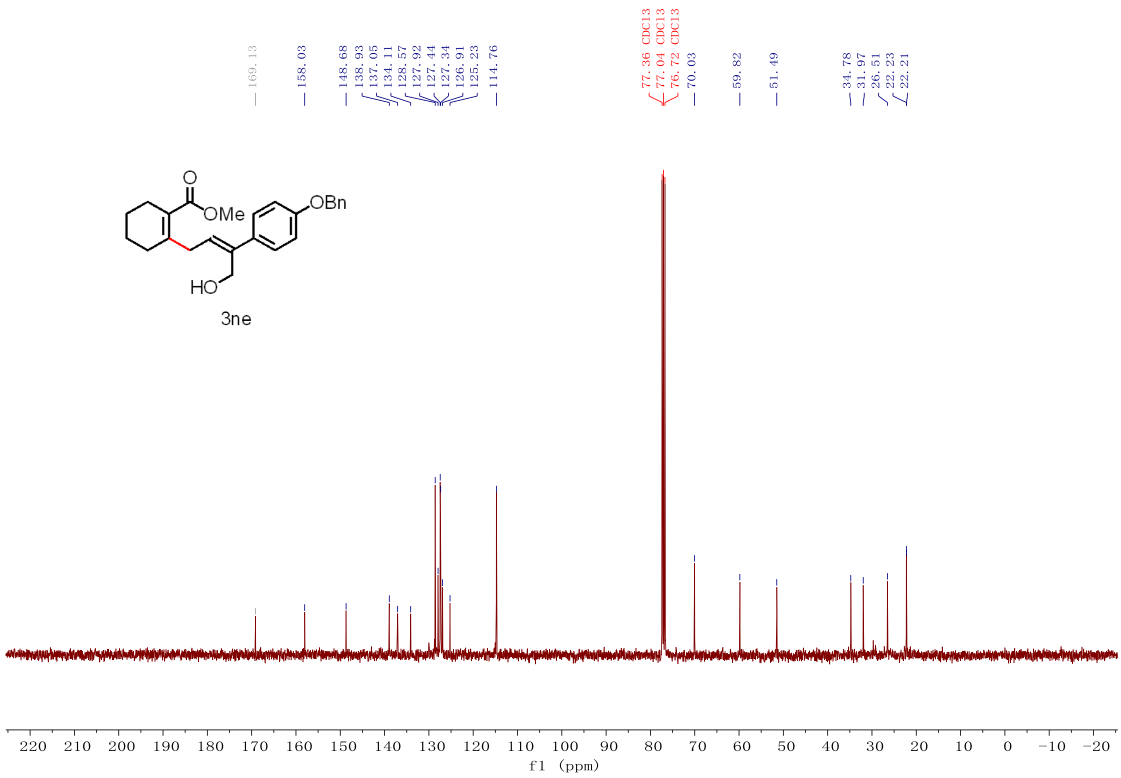

Supplementary Figure 52 ^{13}C NMR (101 MHz, $CDCl_3$) spectrum of compound **3ne**

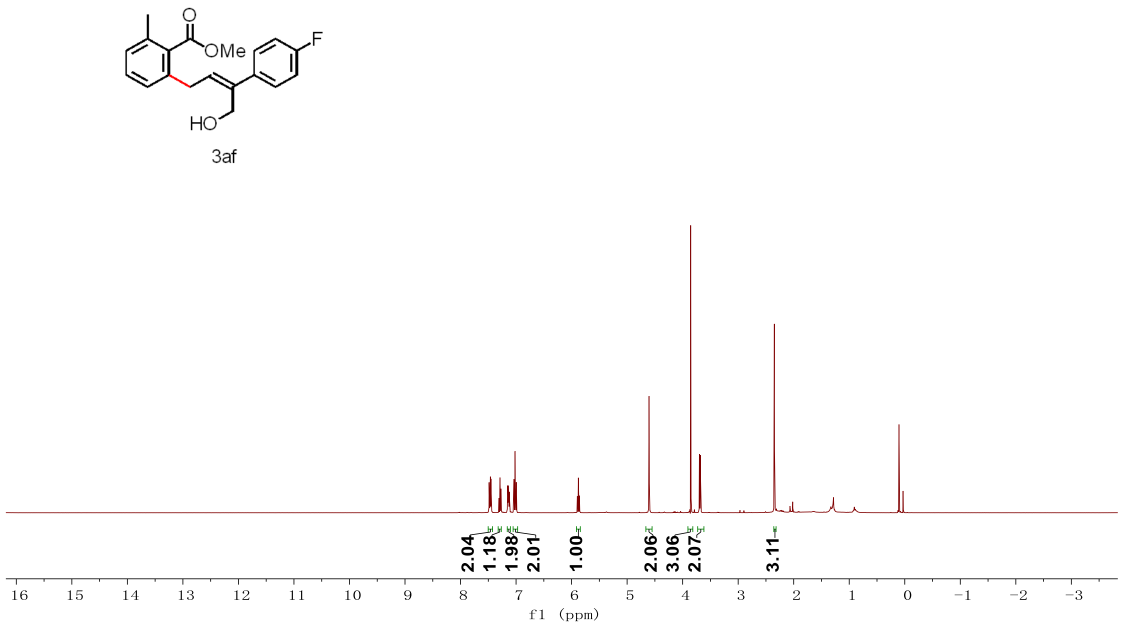

Supplementary Figure 53 ^{1}H NMR (400 MHz, $CDCl_3$) spectrum of compound **3af**

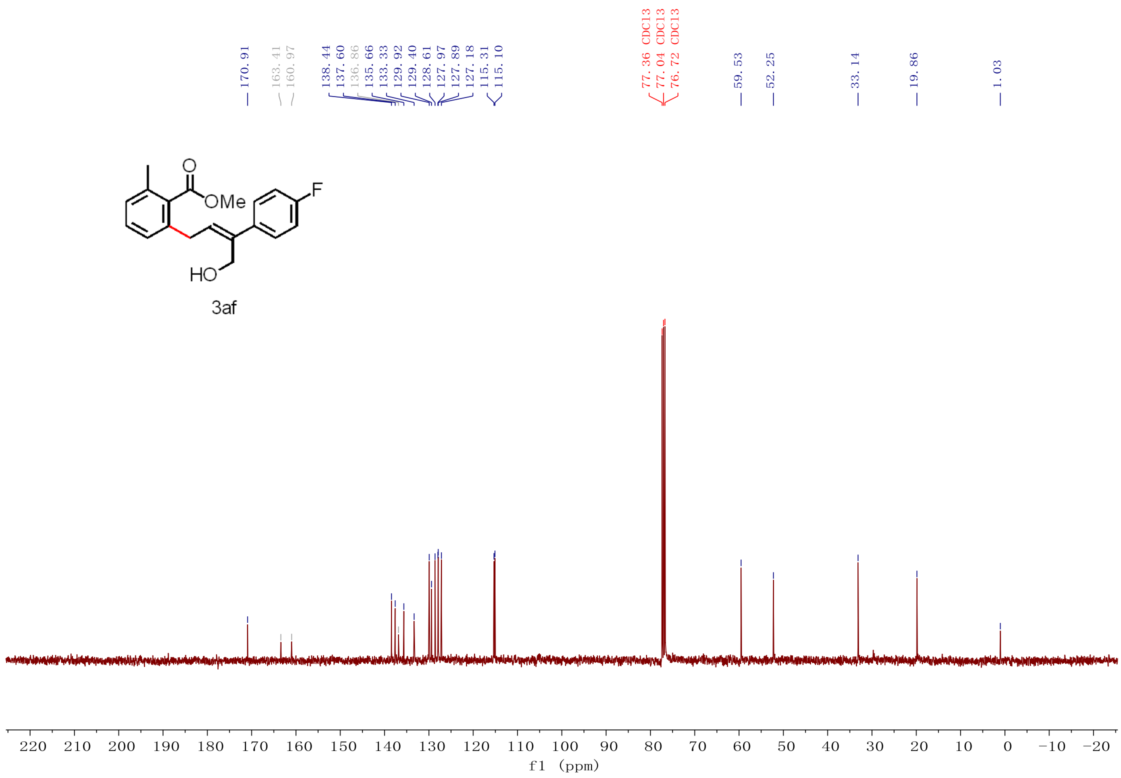

Supplementary Figure 54 ^{13}C NMR (101 MHz, $CDCl_3$) spectrum of compound **3af**

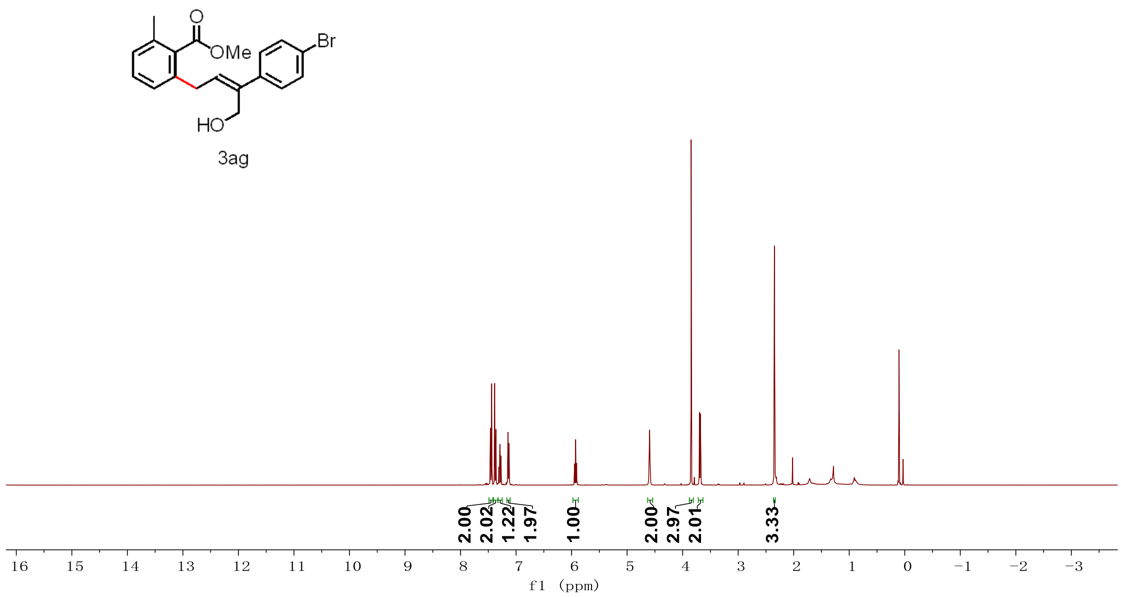

Supplementary Figure 55 ^{1}H NMR (400 MHz, $CDCl_3$) spectrum of compound **3ag**

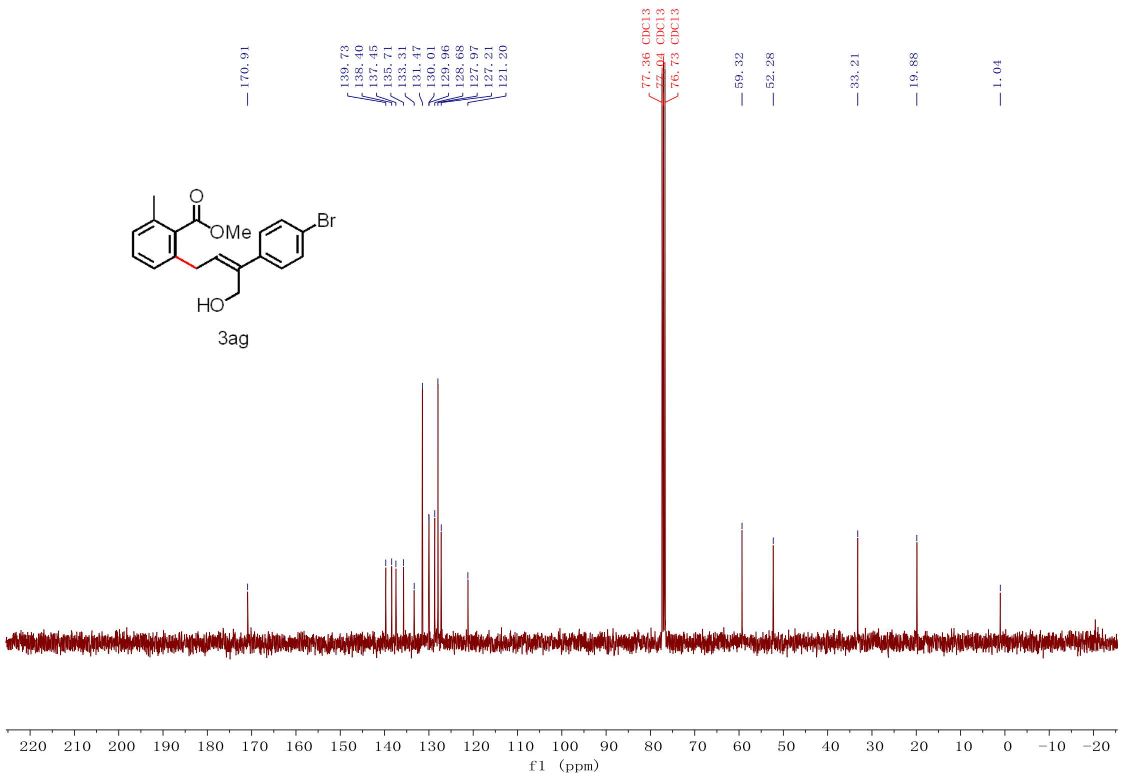

Supplementary Figure 56 ^{13}C NMR (101 MHz, $CDCl_3$) spectrum of compound **3ag**

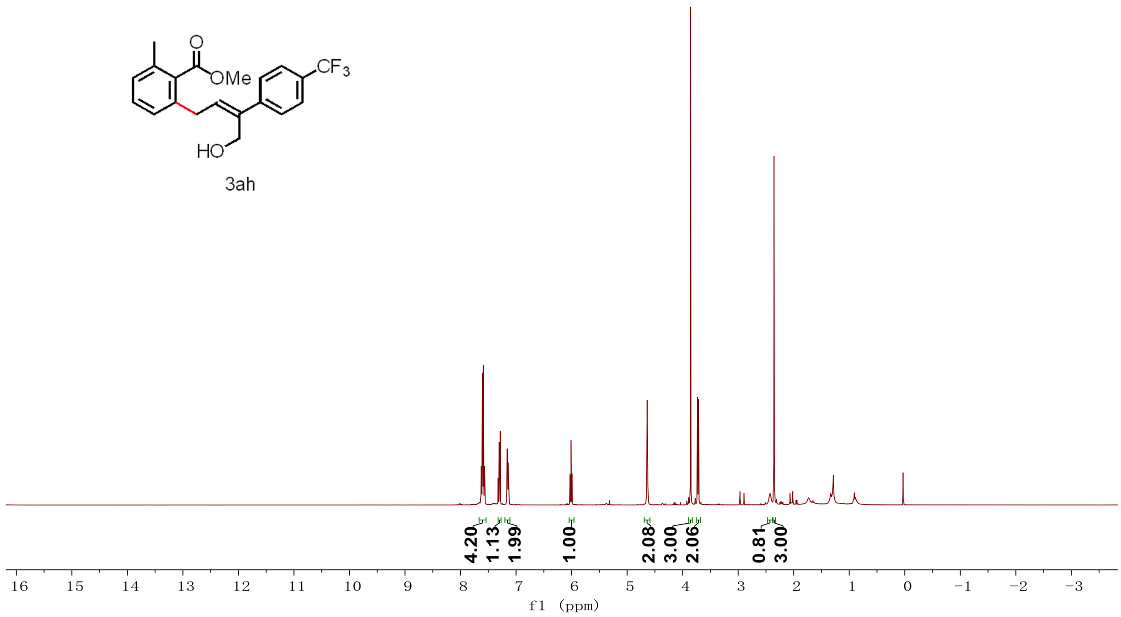

Supplementary Figure 57 ^{1}H NMR (400 MHz, $CDCl_3$) spectrum of compound **3ah**

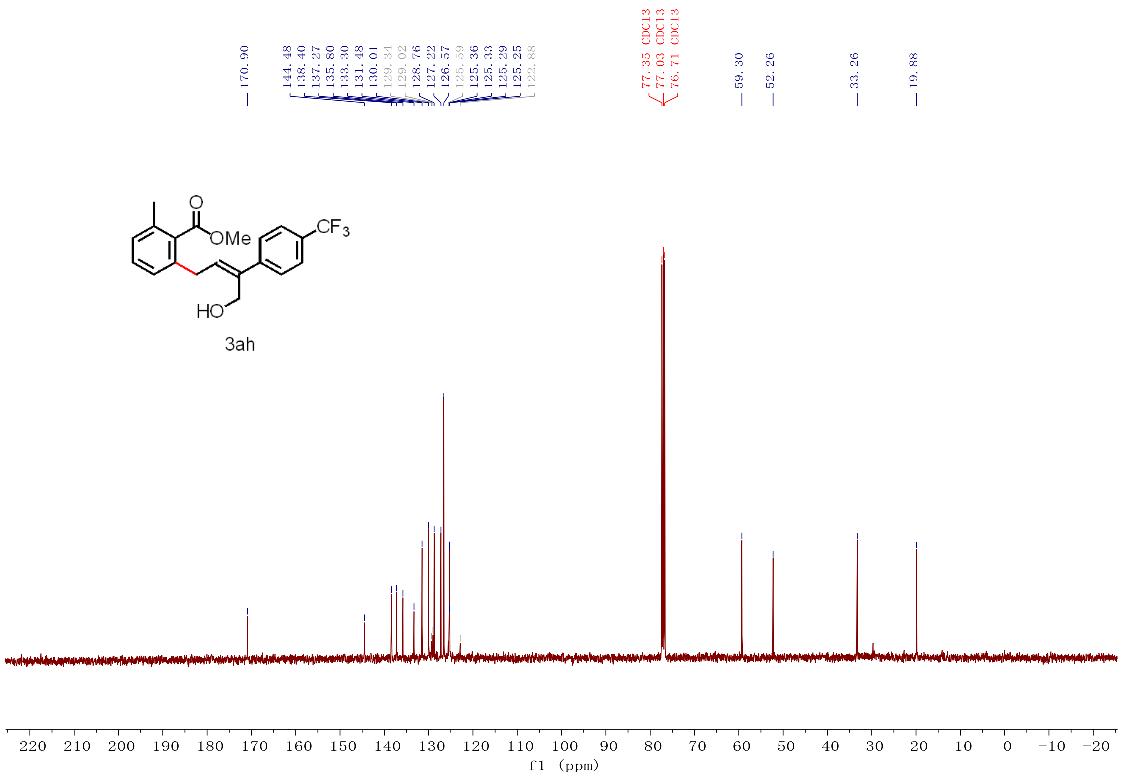

Supplementary Figure 58 ^{13}C NMR (101 MHz, $CDCl_3$) spectrum of compound **3ah**

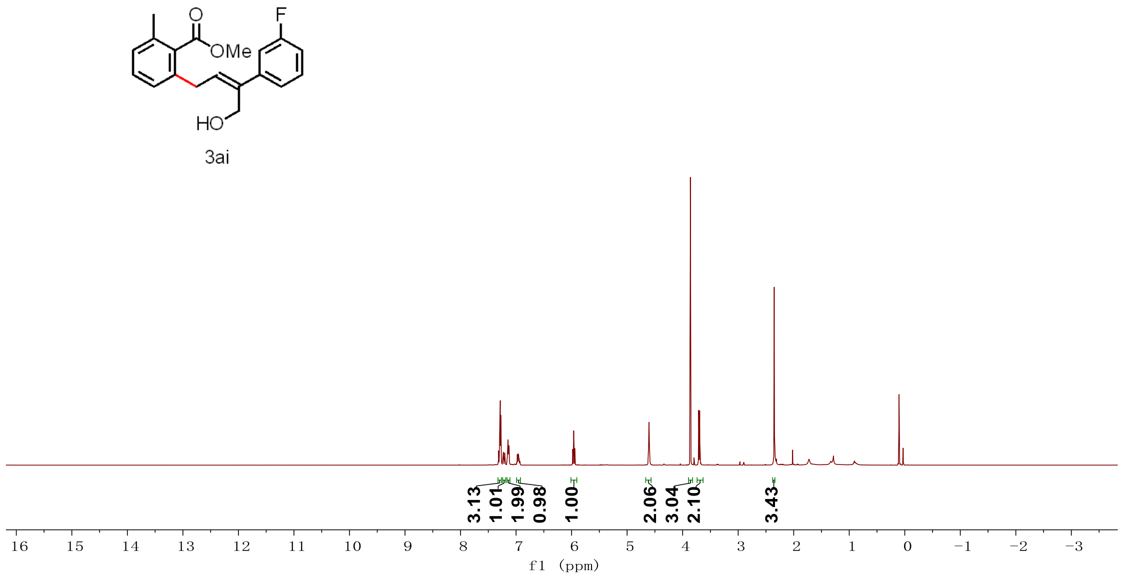

Supplementary Figure 59 ^{1}H NMR (400 MHz, $CDCl_3$) spectrum of compound **3ai**

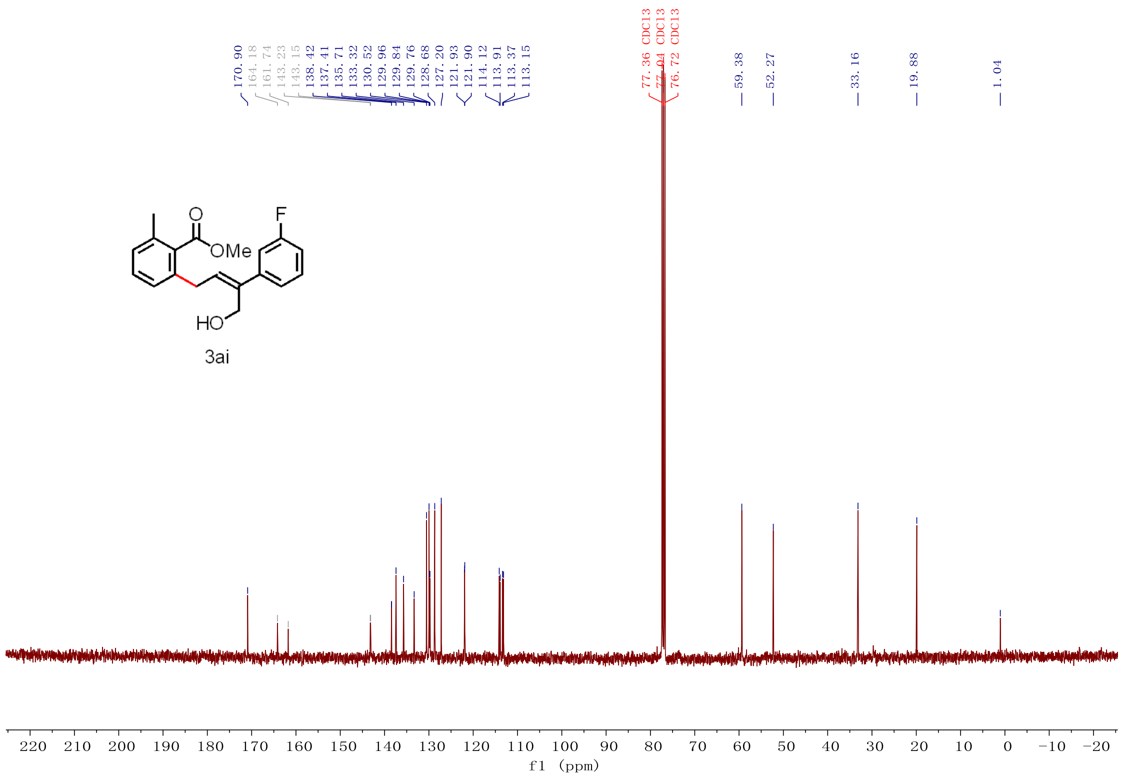

Supplementary Figure 60 ^{13}C NMR (101 MHz, $CDCl_3$) spectrum of compound **3ai**

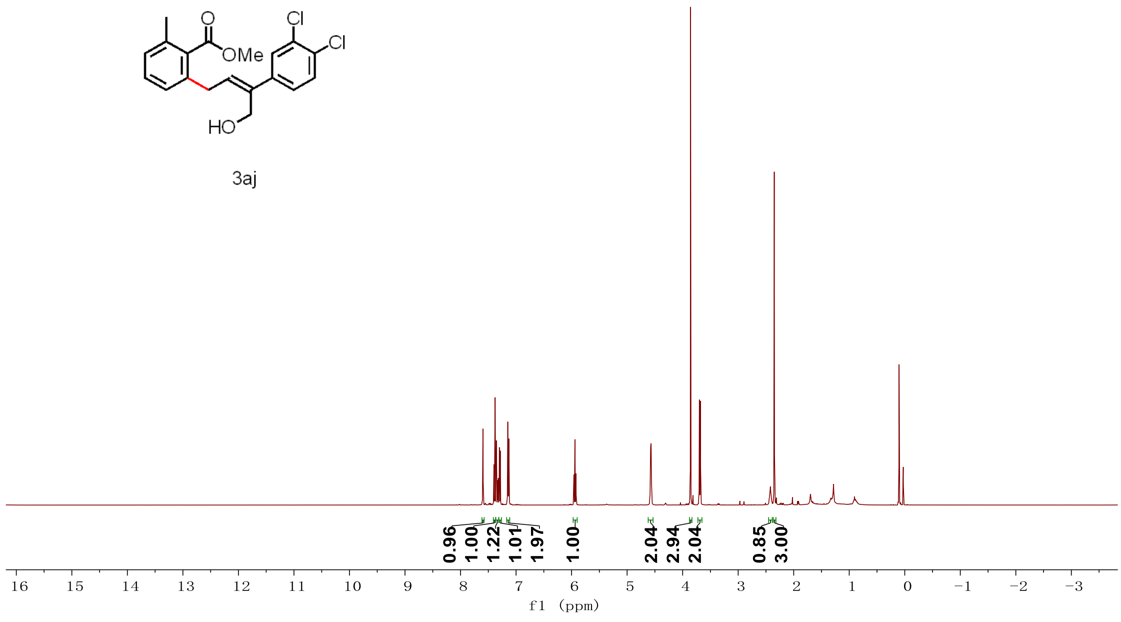

Supplementary Figure 61 ^{1}H NMR (400 MHz, $CDCl_3$) spectrum of compound **3aj**

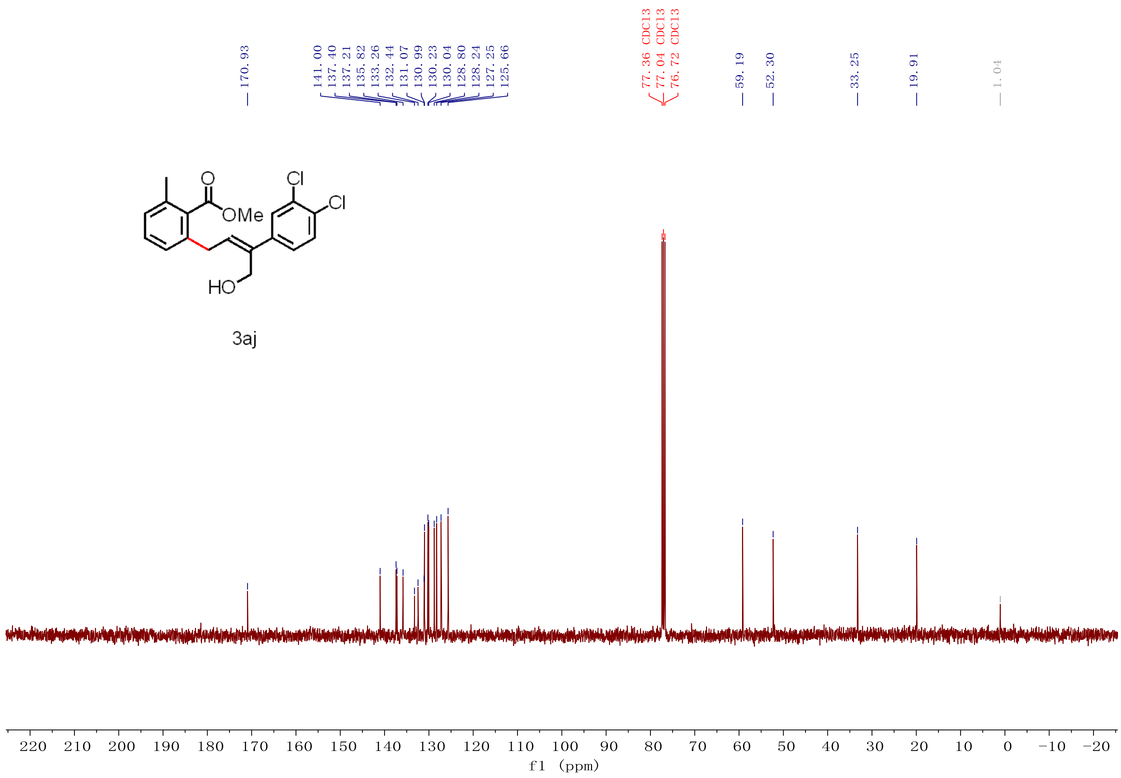

Supplementary Figure 62 ^{13}C NMR (101 MHz, $CDCl_3$) spectrum of compound **3aj**

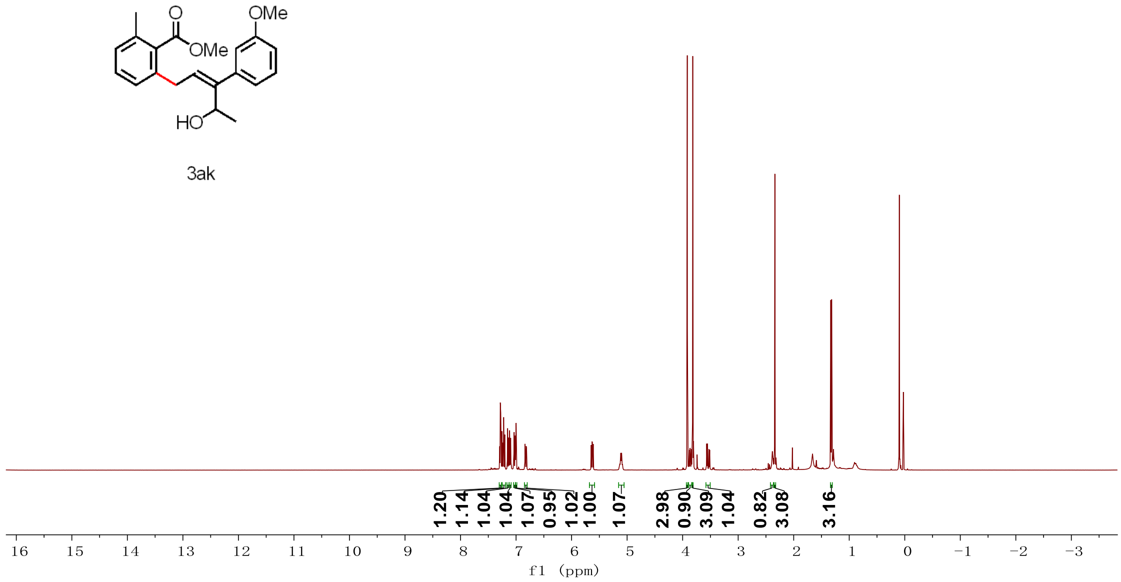

Supplementary Figure 63 ^{1}H NMR (400 MHz, $CDCl_3$) spectrum of compound **3ak**

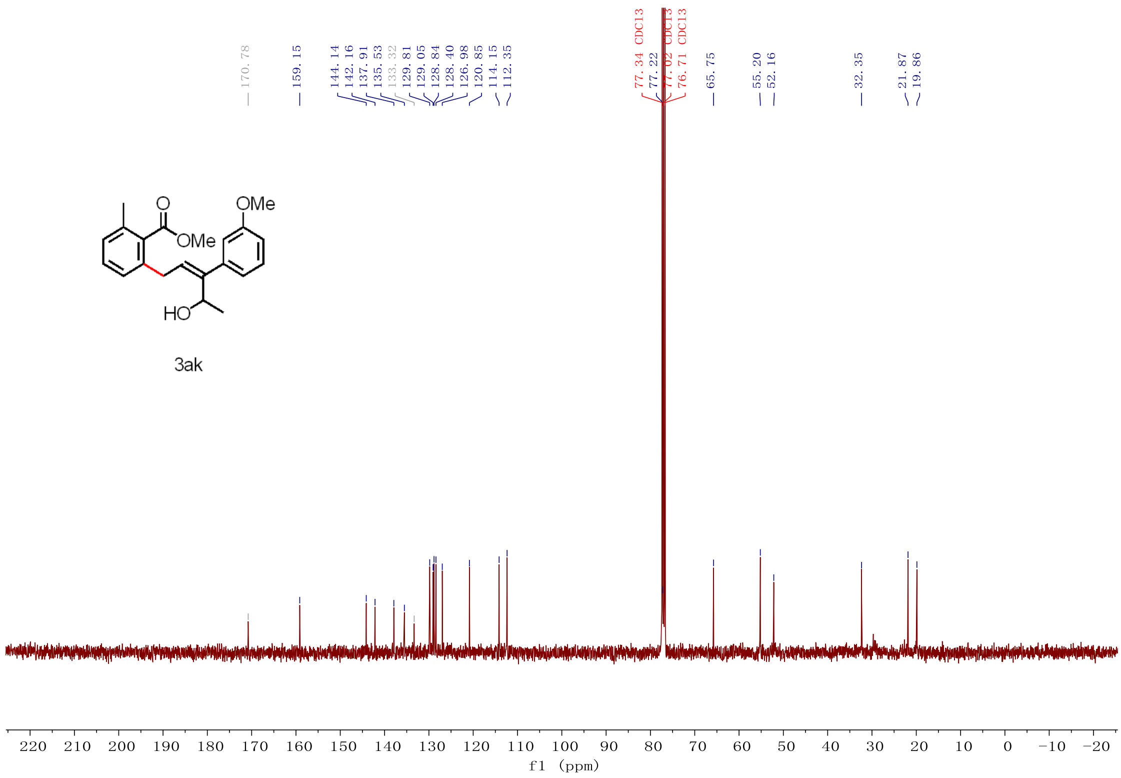

Supplementary Figure 64 ^{13}C NMR (101 MHz, $CDCl_3$) spectrum of compound **3ak**

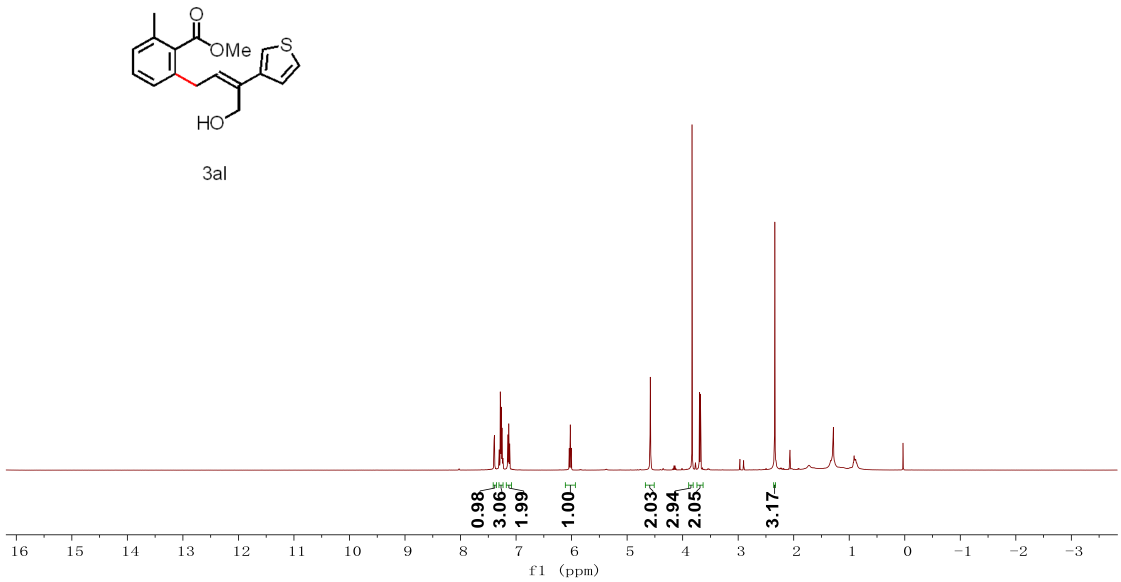

Supplementary Figure 65 ^{1}H NMR (400 MHz, $CDCl_3$) spectrum of compound **3al**

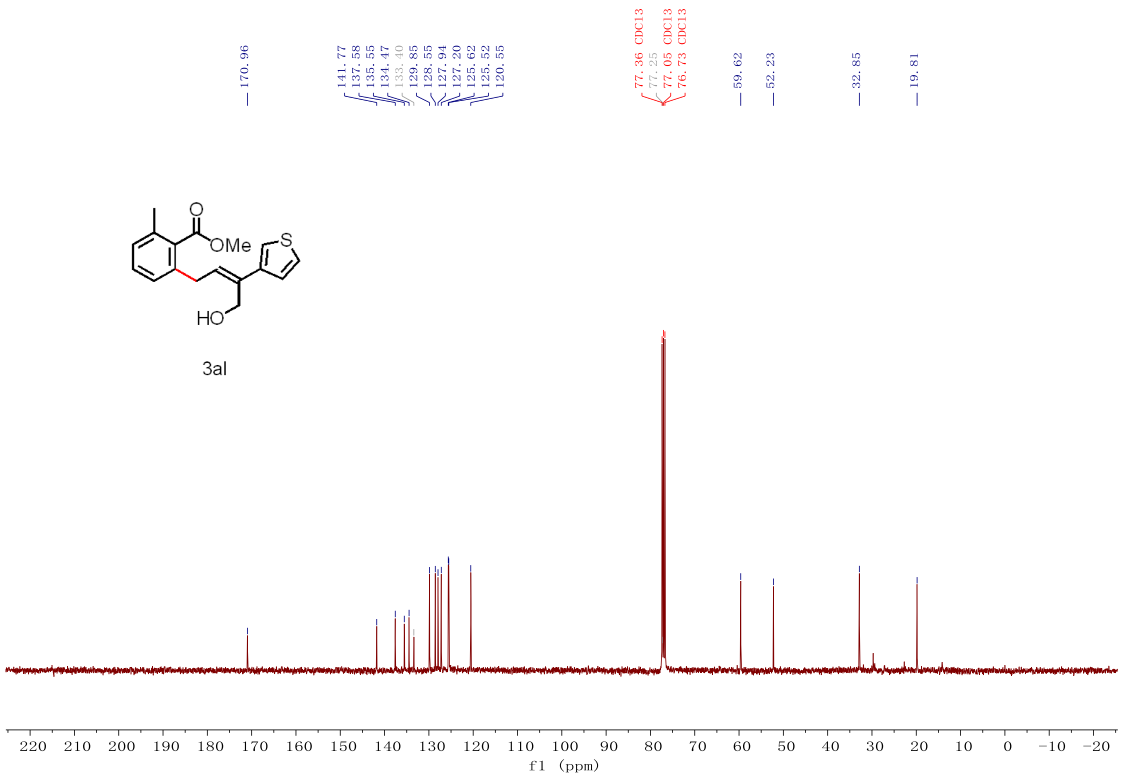

Supplementary Figure 66 ^{13}C NMR (101 MHz, $CDCl_3$) spectrum of compound **3al**

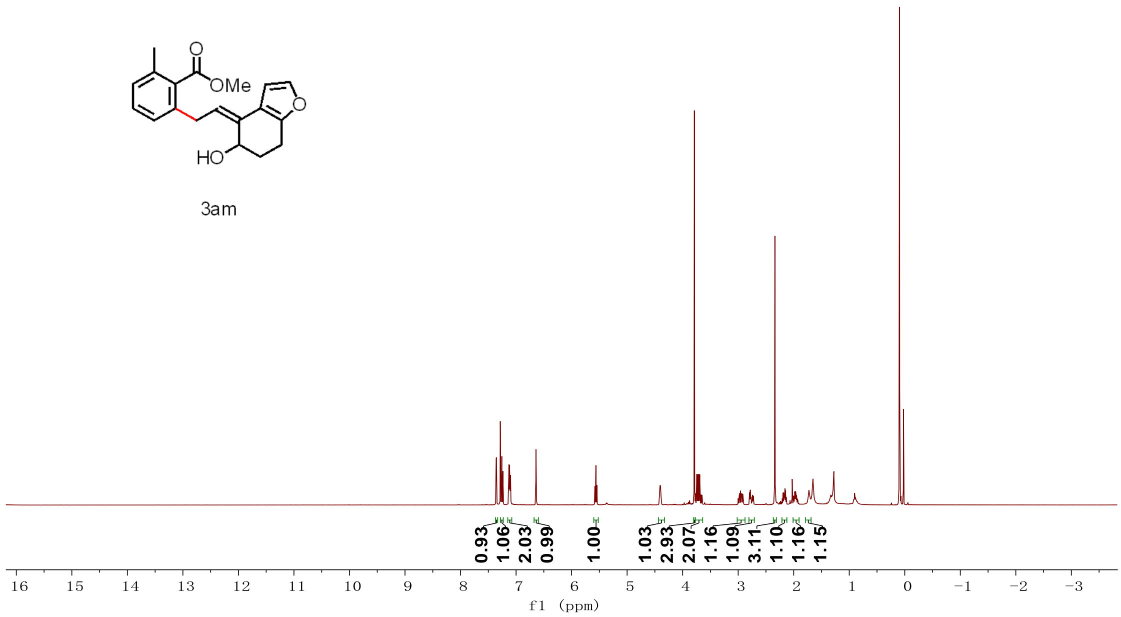

Supplementary Figure 67 ^{1}H NMR (400 MHz, $CDCl_3$) spectrum of compound **3am**

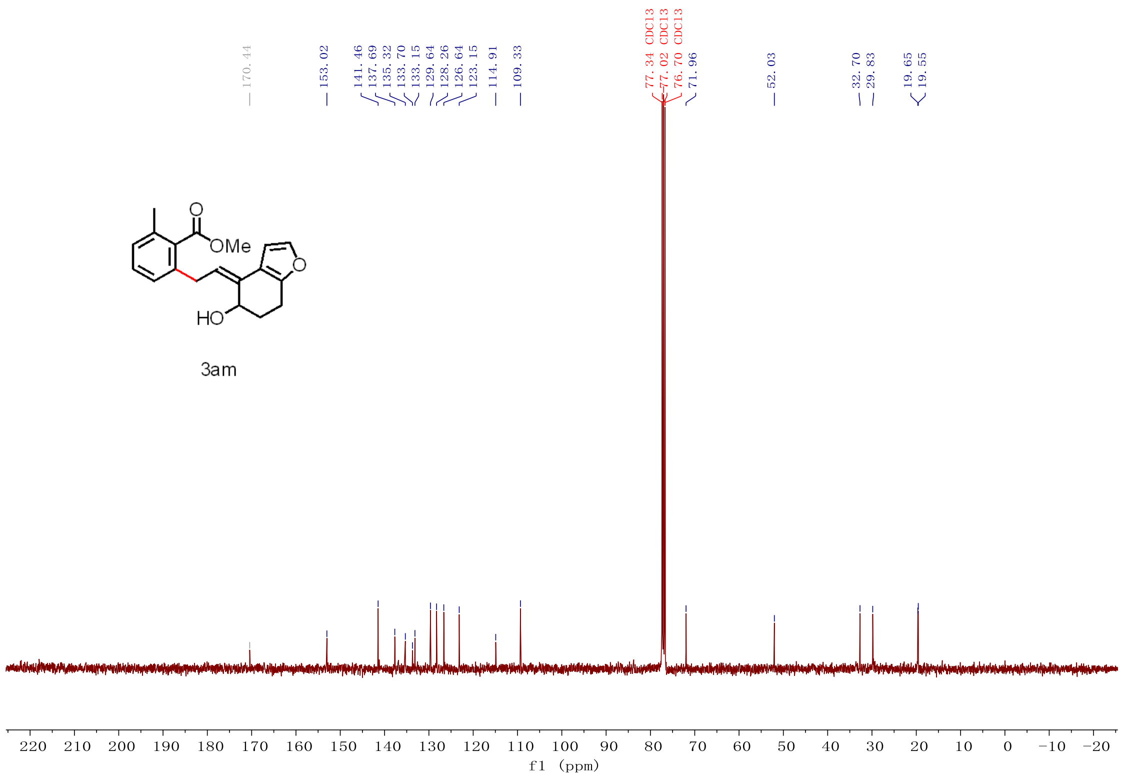

Supplementary Figure 68 ^{13}C NMR (101 MHz, $CDCl_3$) spectrum of compound **3am**

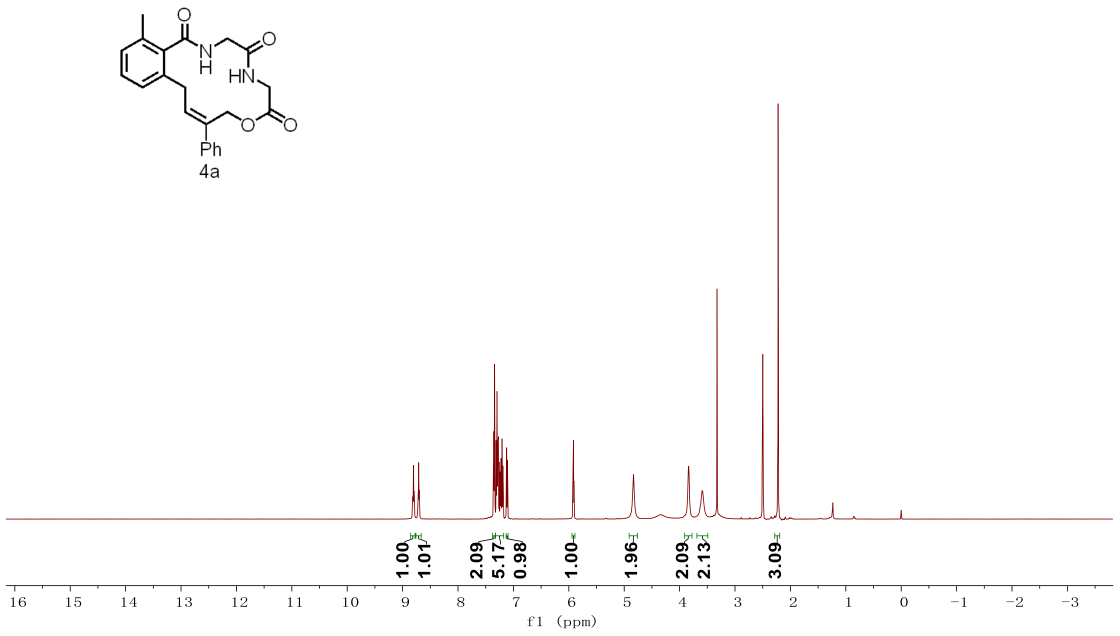

Supplementary Figure 69 ^{1}H NMR (500 MHz, DMSO-d_6) spectrum of compound **4a**

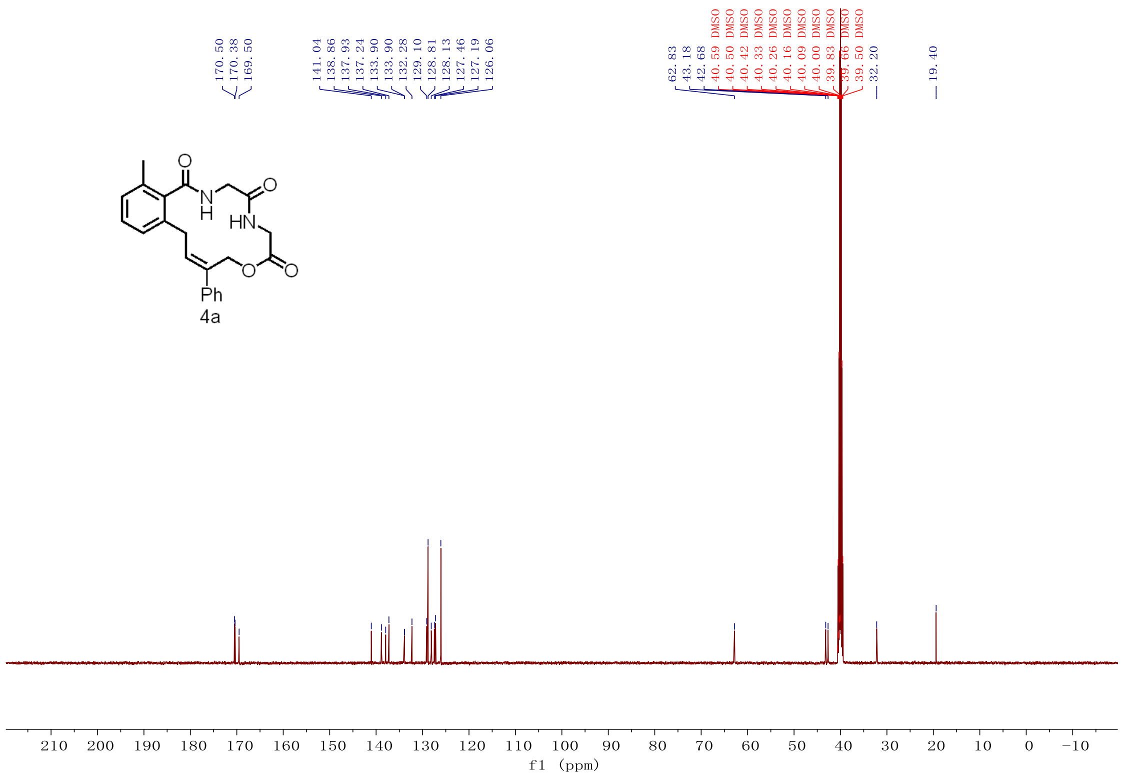

Supplementary Figure 70 ^{13}C NMR (126 MHz, DMSO) spectrum of compound **4a**

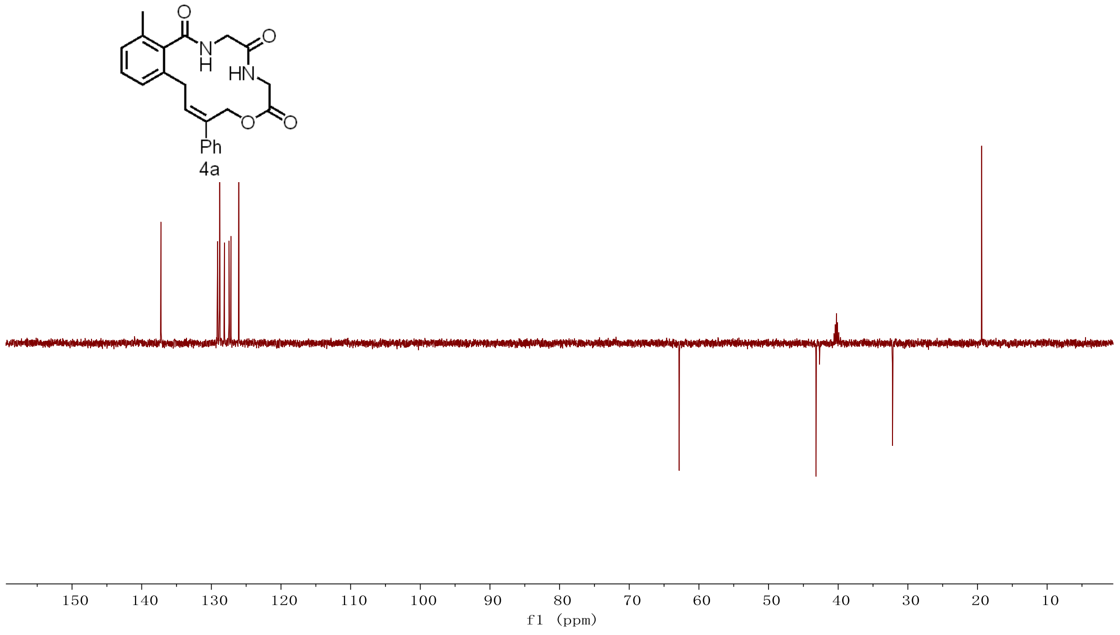

Supplementary Figure 71 ^{13}C NMR (126 MHz, DMSO) spectrum of compound **4a**

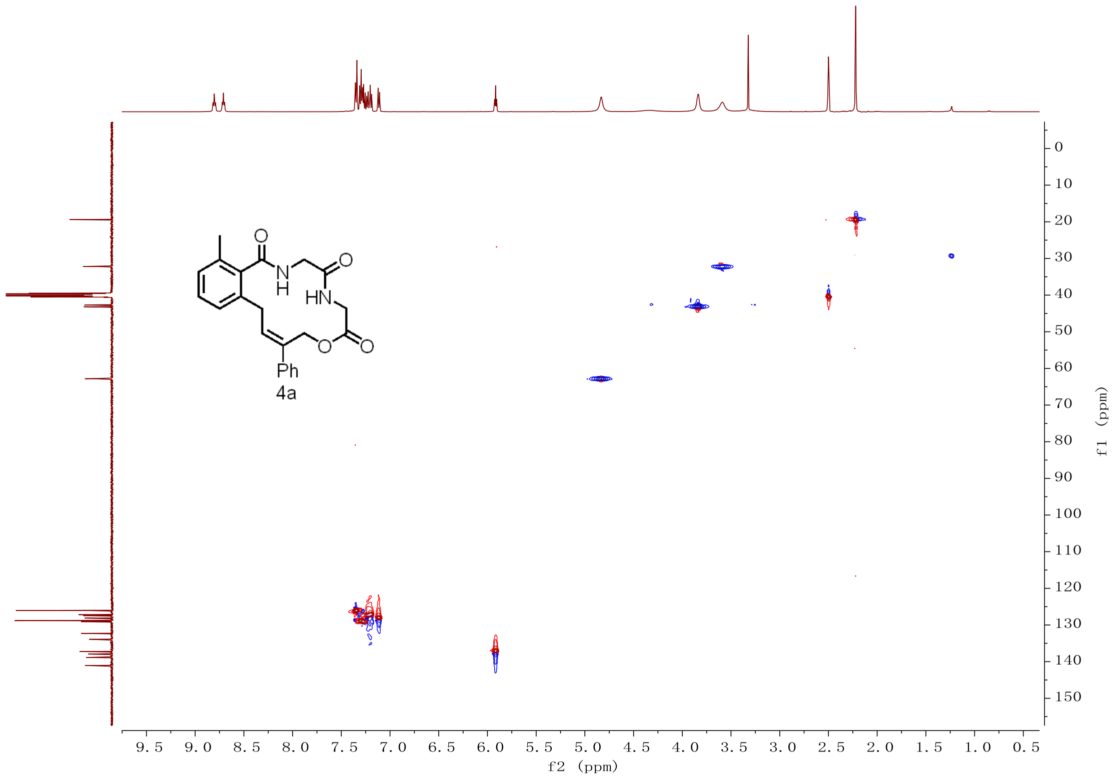

Supplementary Figure 72 HSQC of compound **4a**

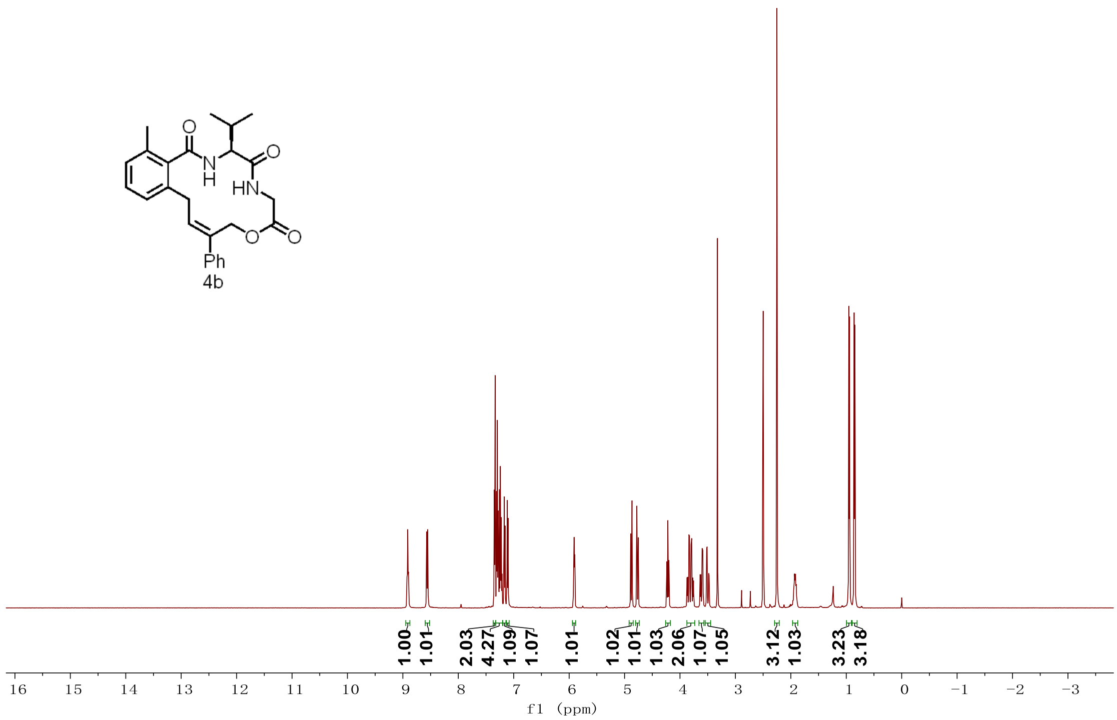

Supplementary Figure 73 ^{1}H NMR (500 MHz, DMSO-d_6) spectrum of compound **4b**

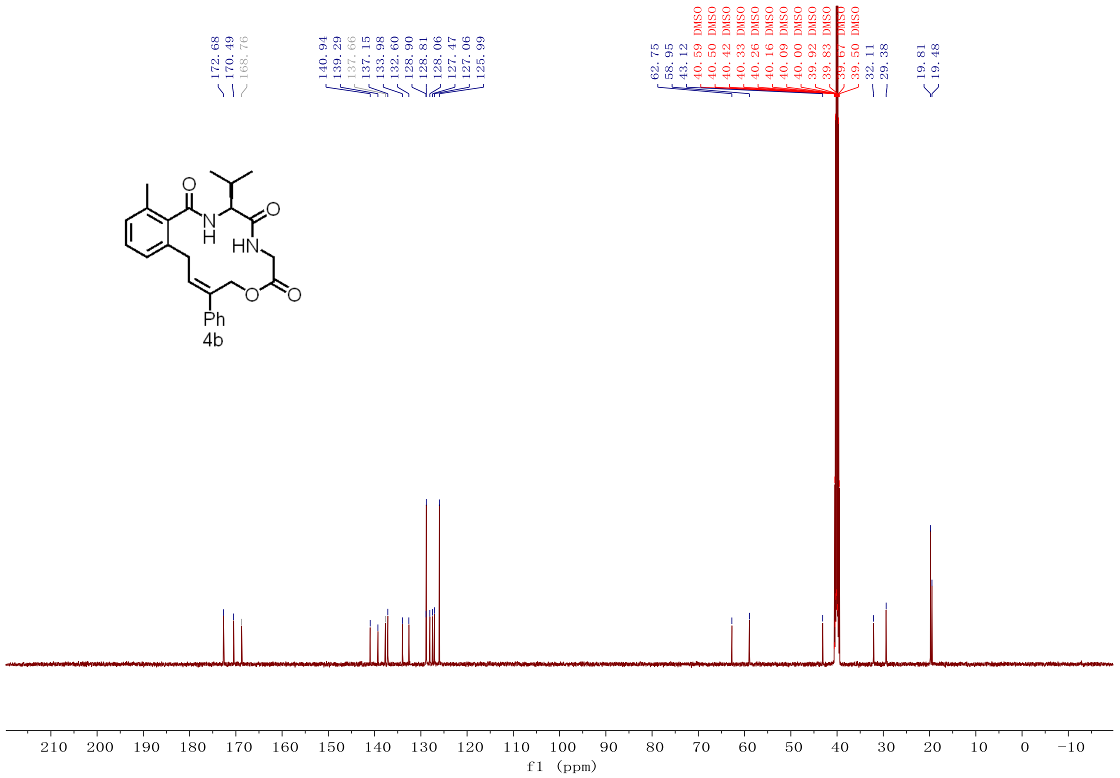

Supplementary Figure 74 ^{13}C NMR (126 MHz, DMSO) spectrum of compound **4b**

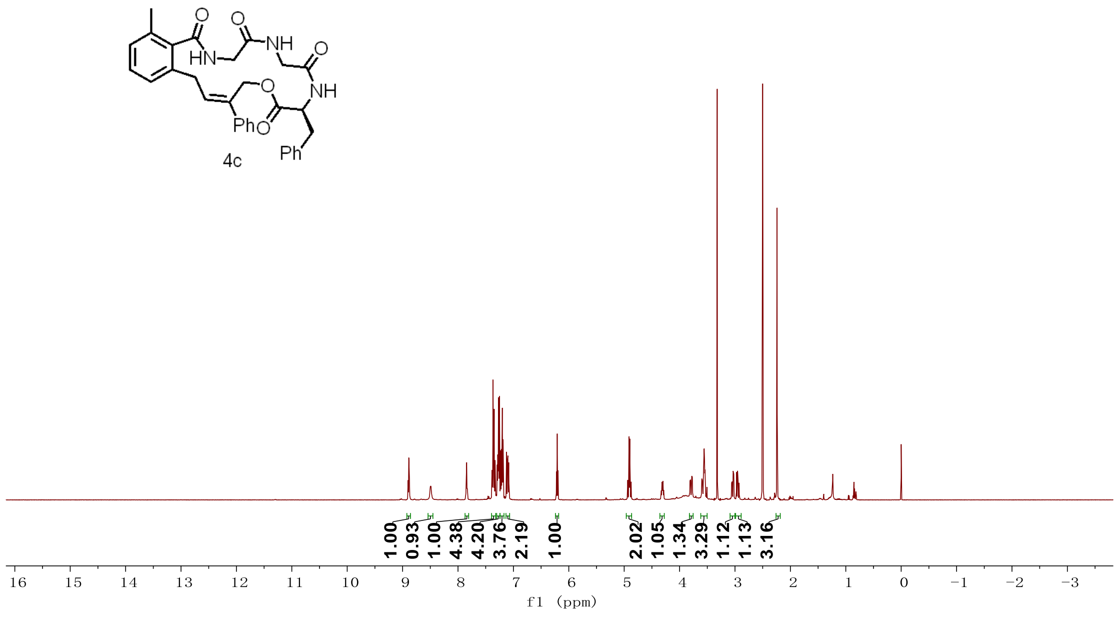

Supplementary Figure 75 ^{1}H NMR (500 MHz, DMSO-d_6) spectrum of compound **4c**

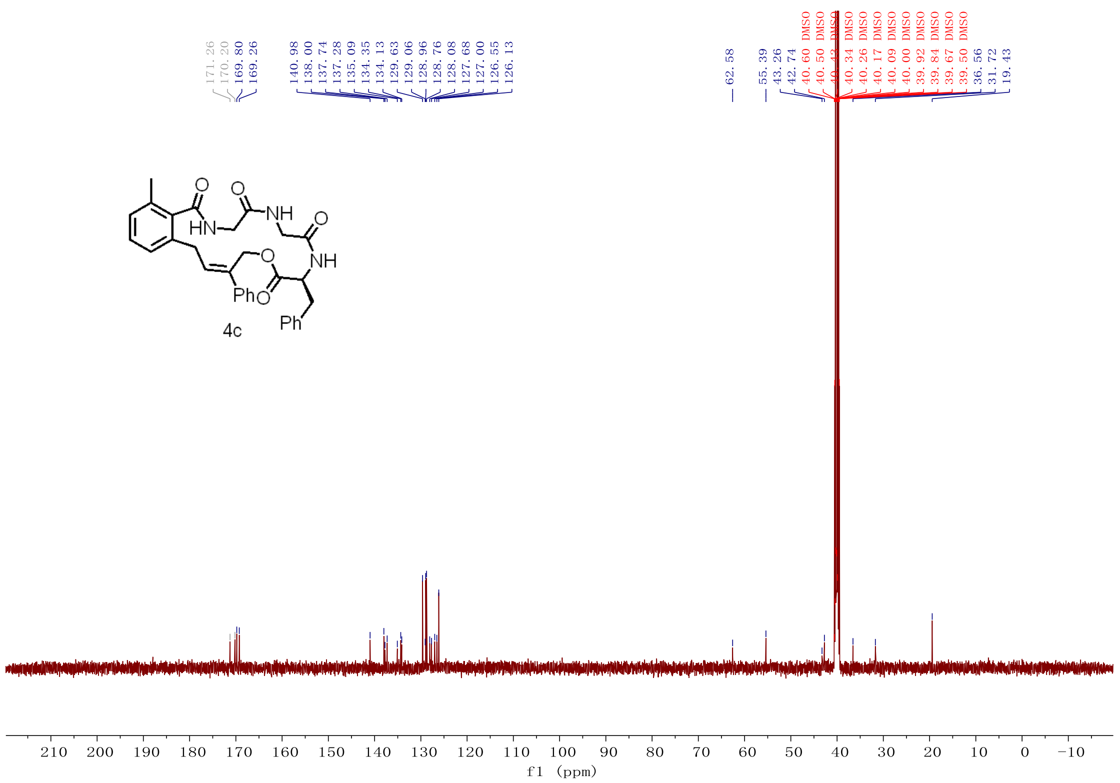

Supplementary Figure 76 ^{13}C NMR (126 MHz, DMSO) spectrum of compound **4c**

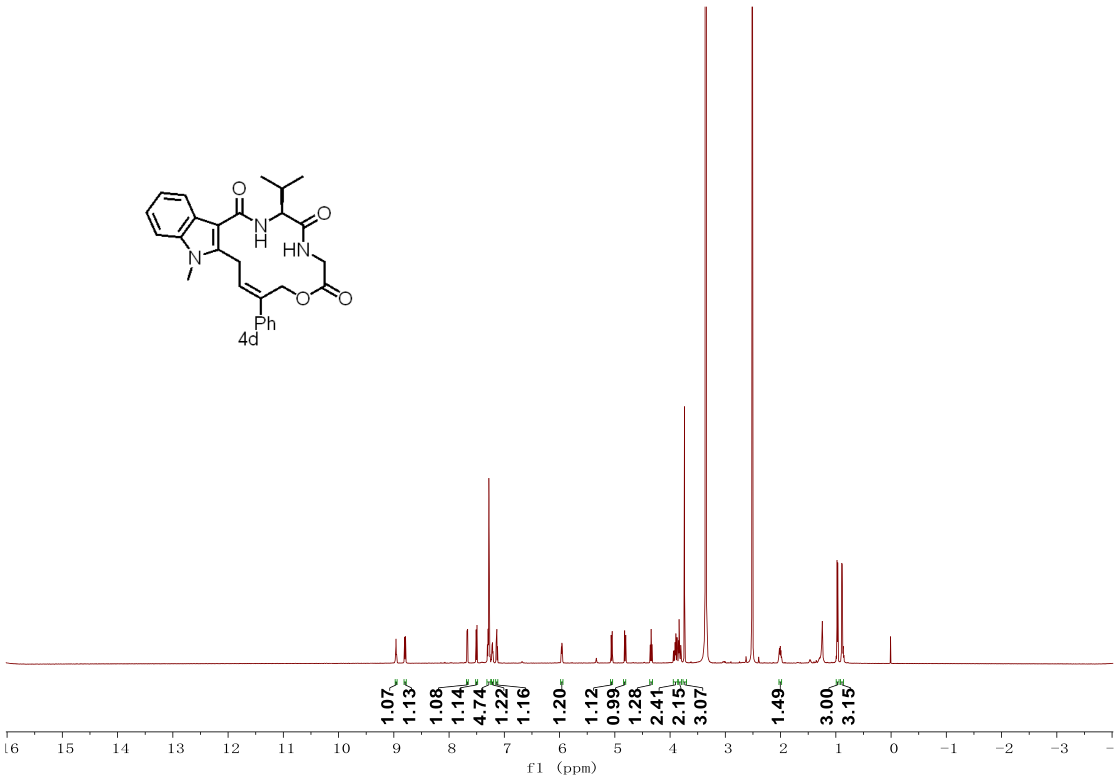

Supplementary Figure 77 ^{1}H NMR (600 MHz, DMSO-d_6) spectrum of compound **4d**

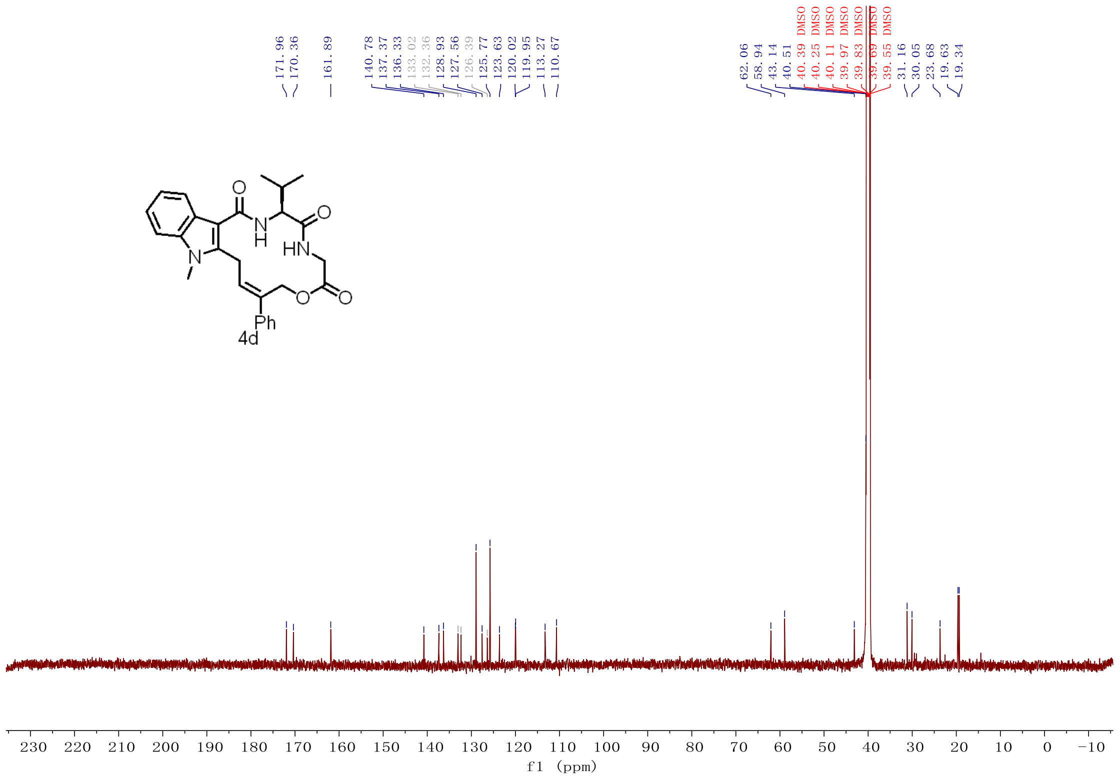

Supplementary Figure 78 ^{13}C NMR (151 MHz, DMSO) spectrum of compound **4d**

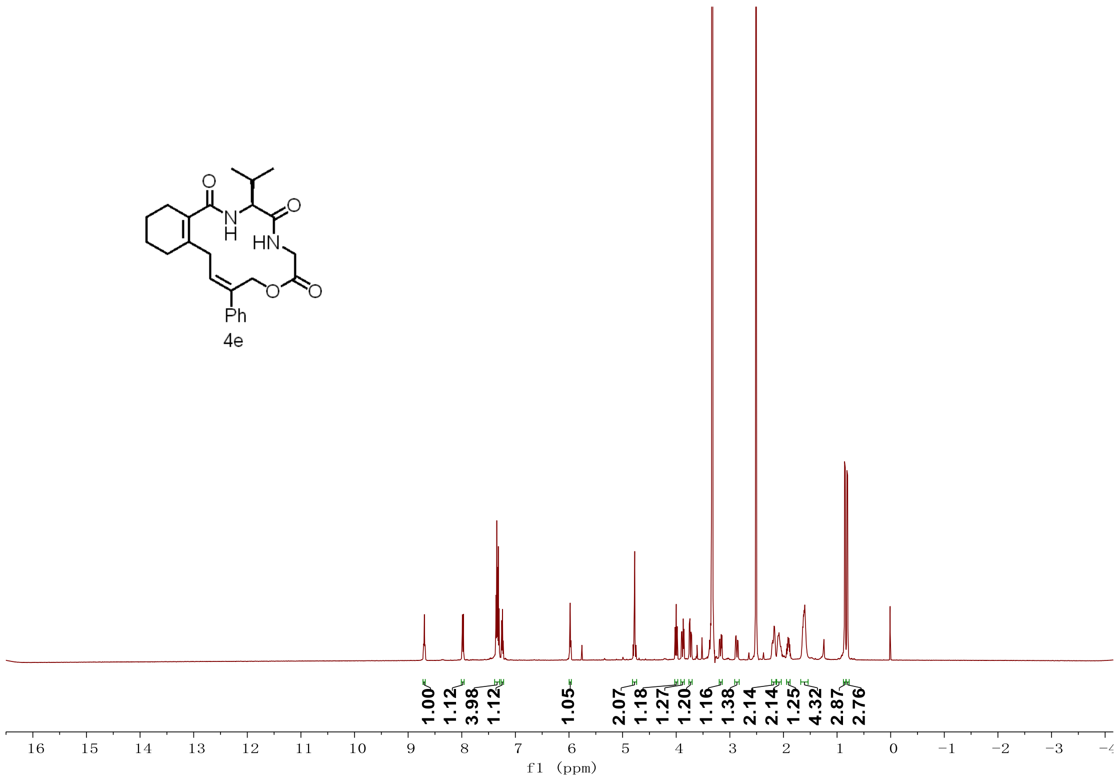

Supplementary Figure 79 ^{1}H NMR (500 MHz, DMSO-d_6) spectrum of compound **4e**

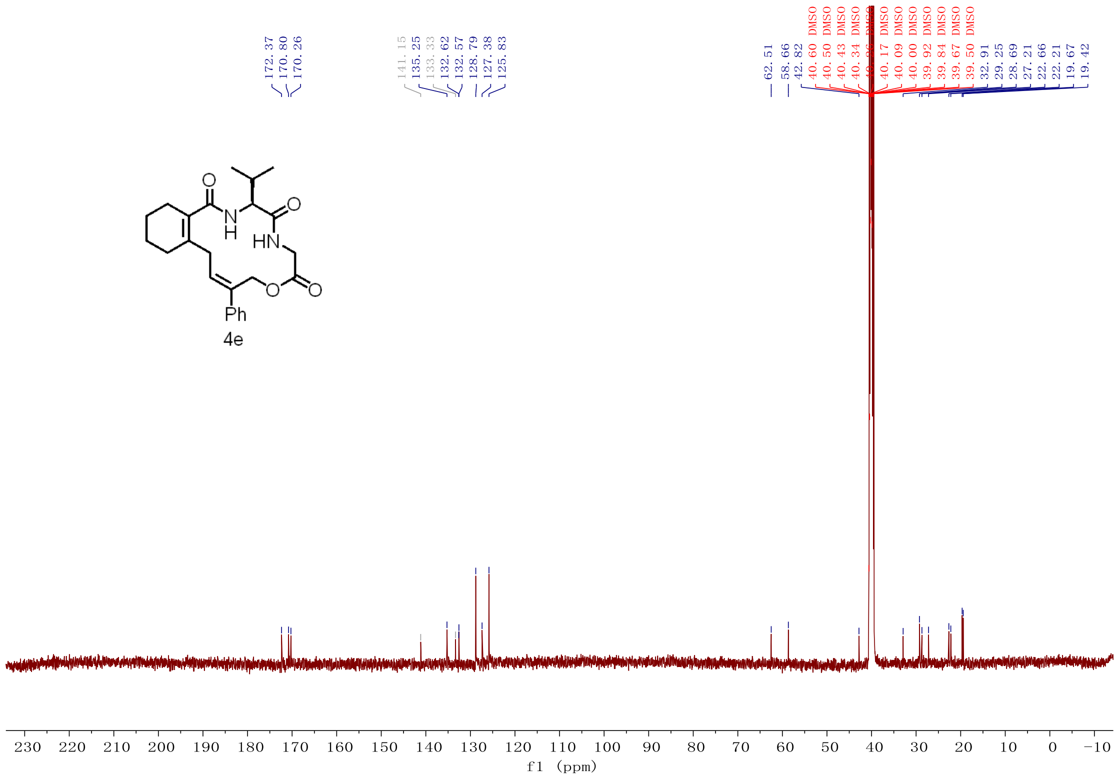

Supplementary Figure 80 ^{13}C NMR (126 MHz, DMSO) spectrum of compound **4e**

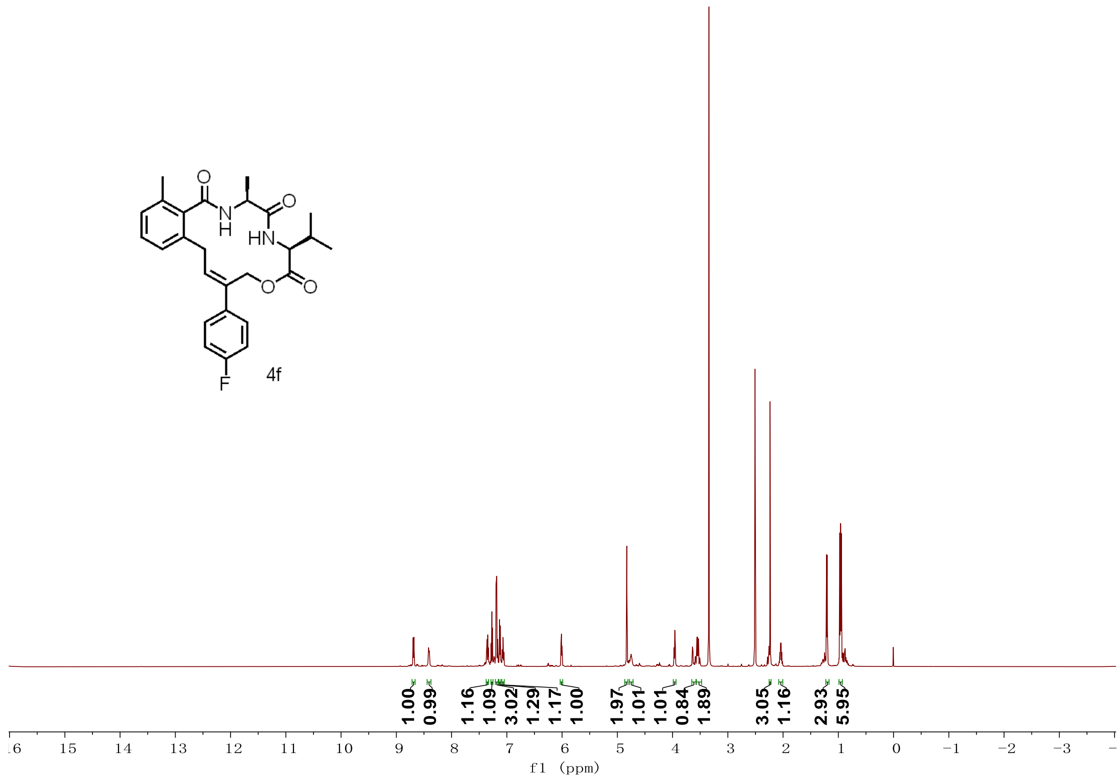

Supplementary Figure 81 ^{1}H NMR (600 MHz, DMSO-d_6) spectrum of compound **4f**

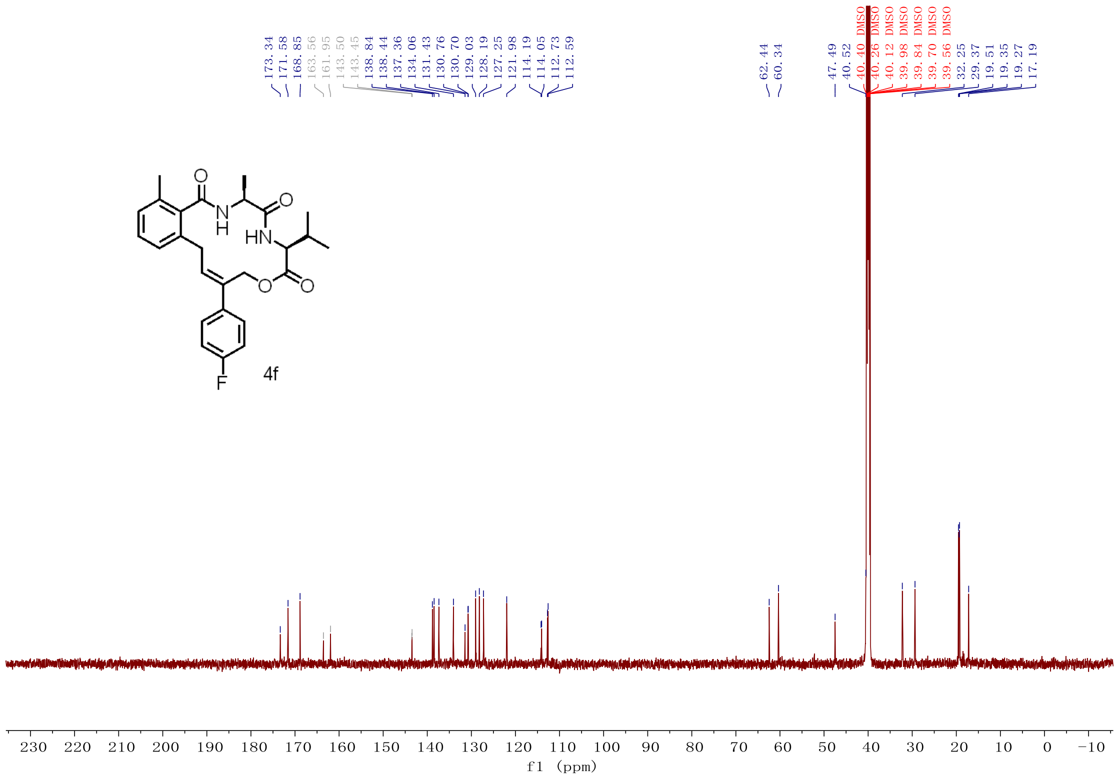

Supplementary Figure 82 ^{13}C NMR (151 MHz, DMSO) spectrum of compound **4f**

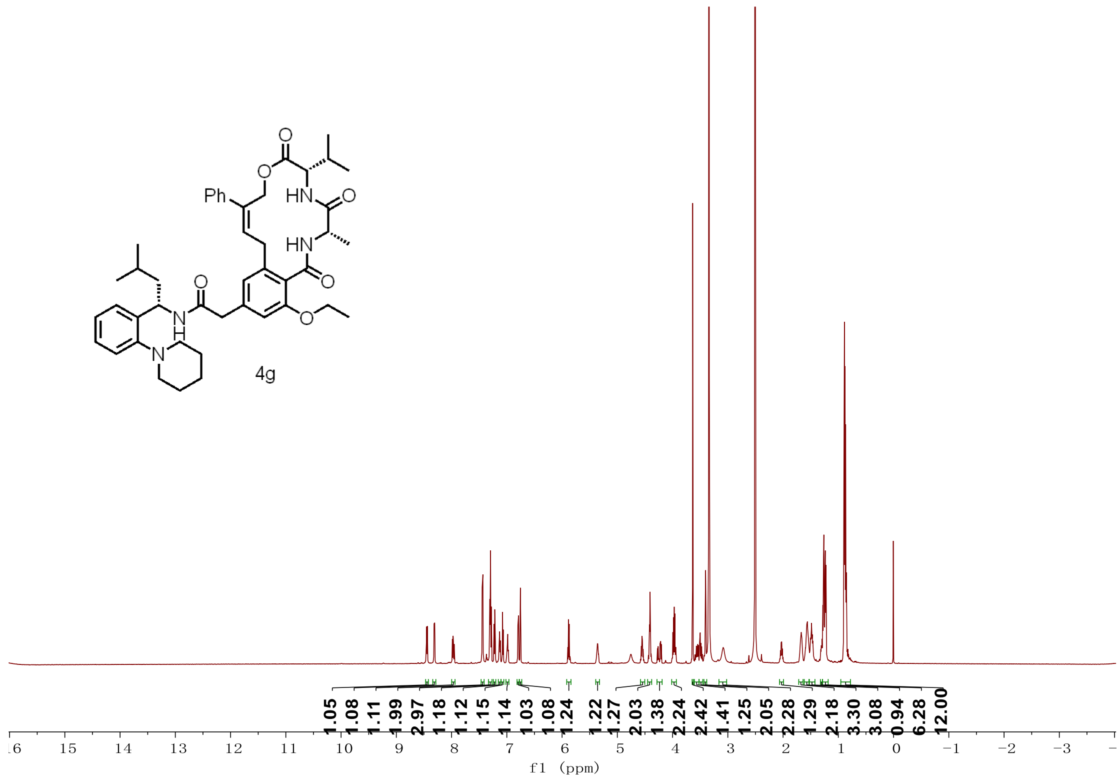

Supplementary Figure 83 ^{1}H NMR (600 MHz, DMSO-d_6) spectrum of compound **4g**

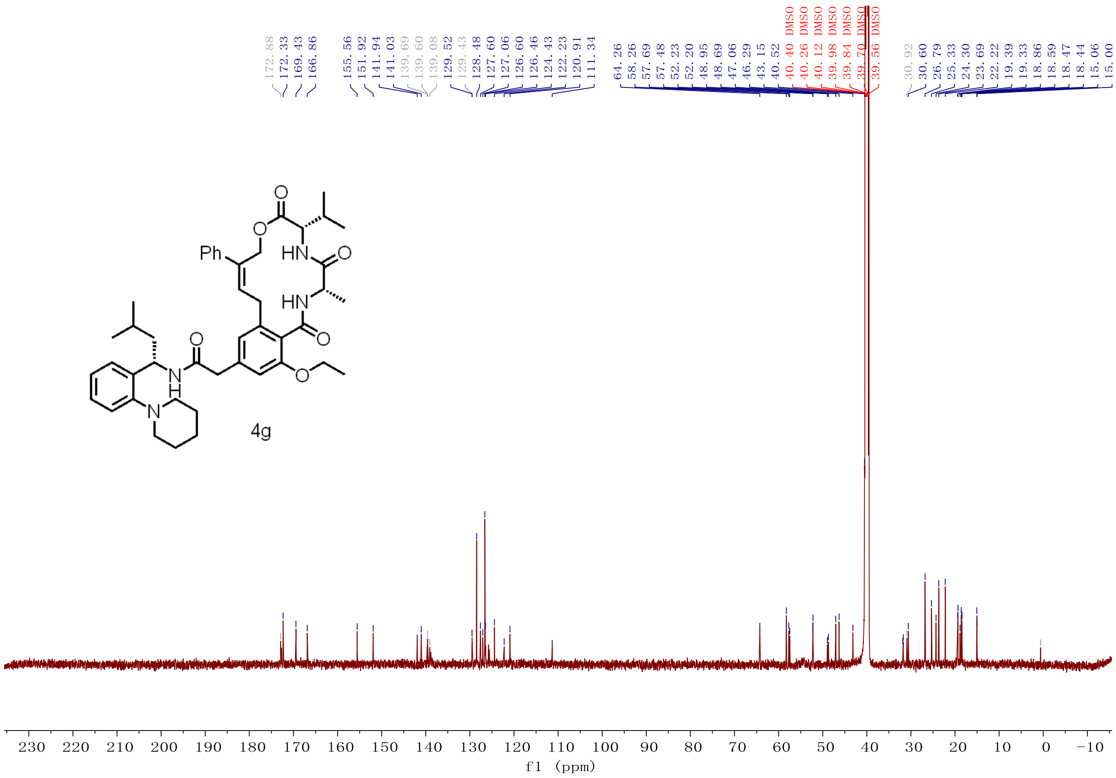

Supplementary Figure 84 ^{13}C NMR (151 MHz, DMSO) spectrum of compound **4g**

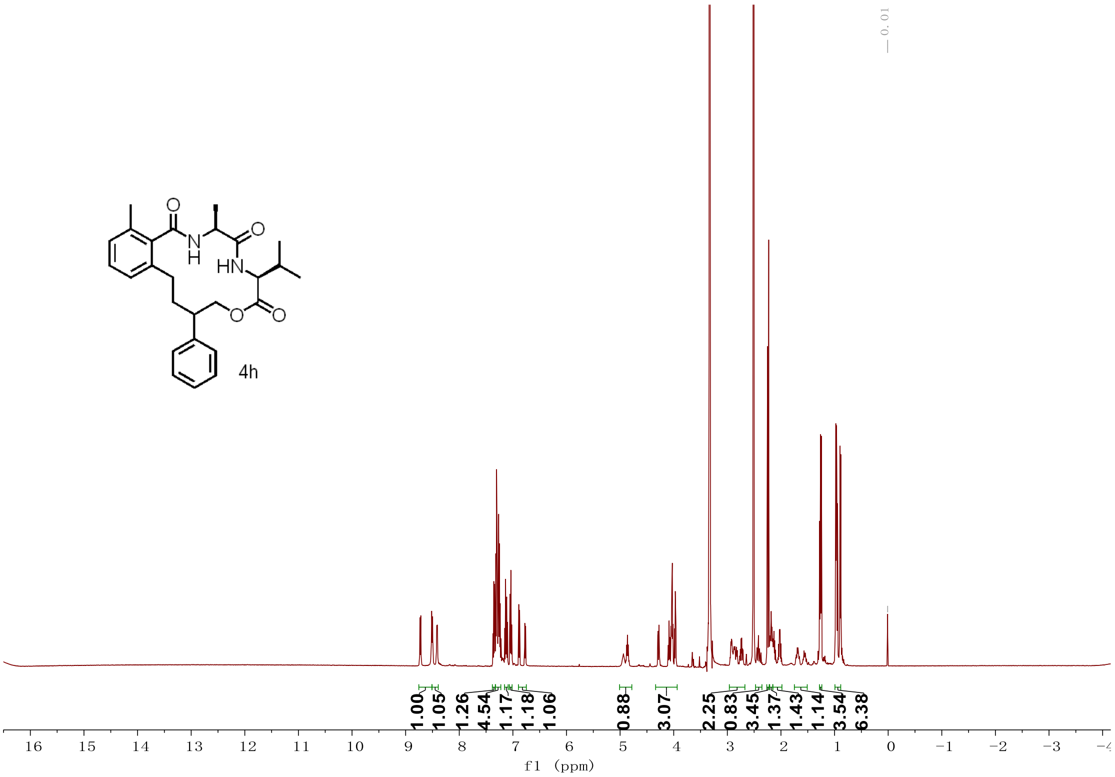

Supplementary Figure 85 ^{1}H NMR (500 MHz, DMSO-d_6) spectrum of compound **4h**

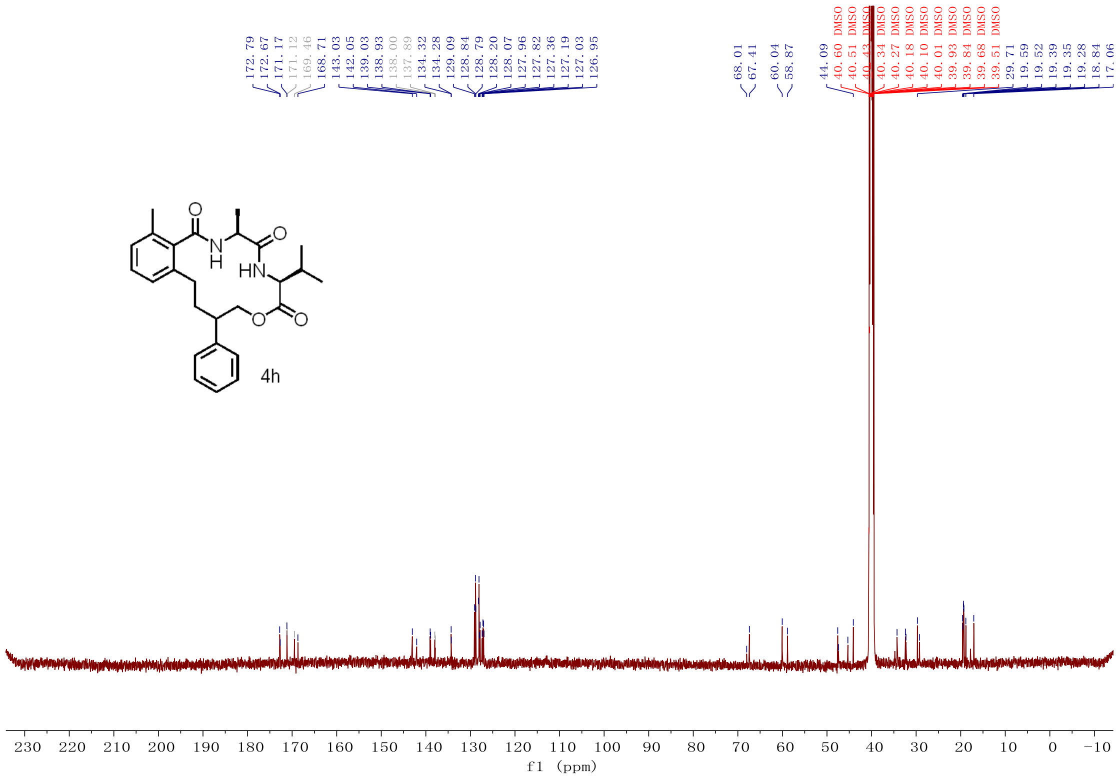

Supplementary Figure 86 ^{13}C NMR (126 MHz, DMSO) spectrum of compound **4h**

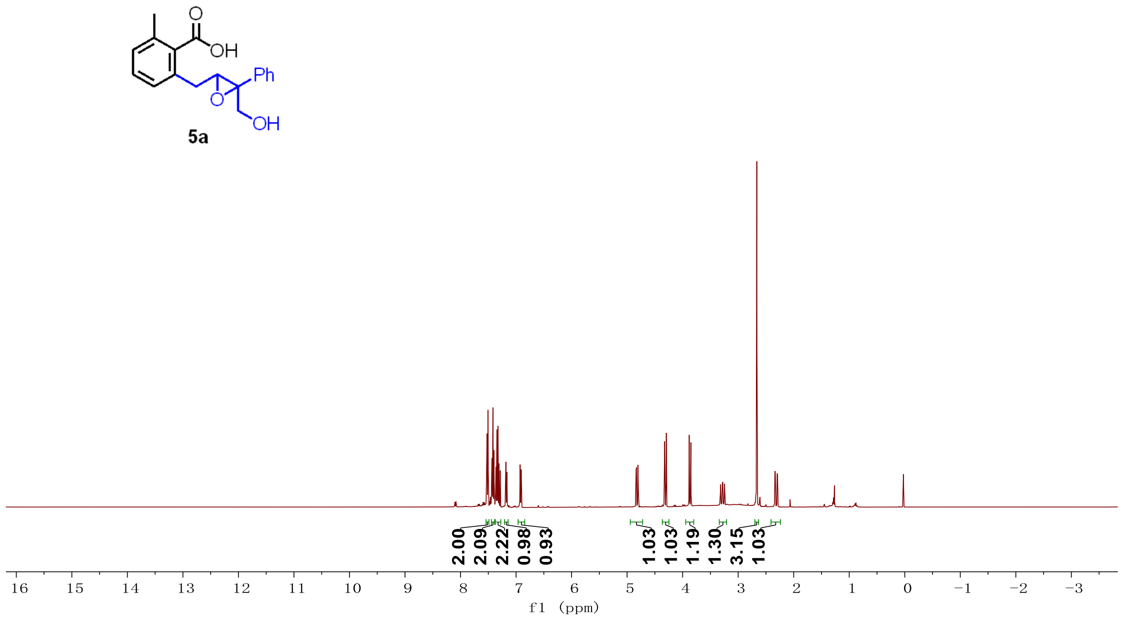

Supplementary Figure 87 ^{1}H NMR (400 MHz, $CDCl_3$) spectrum of compound **5a**

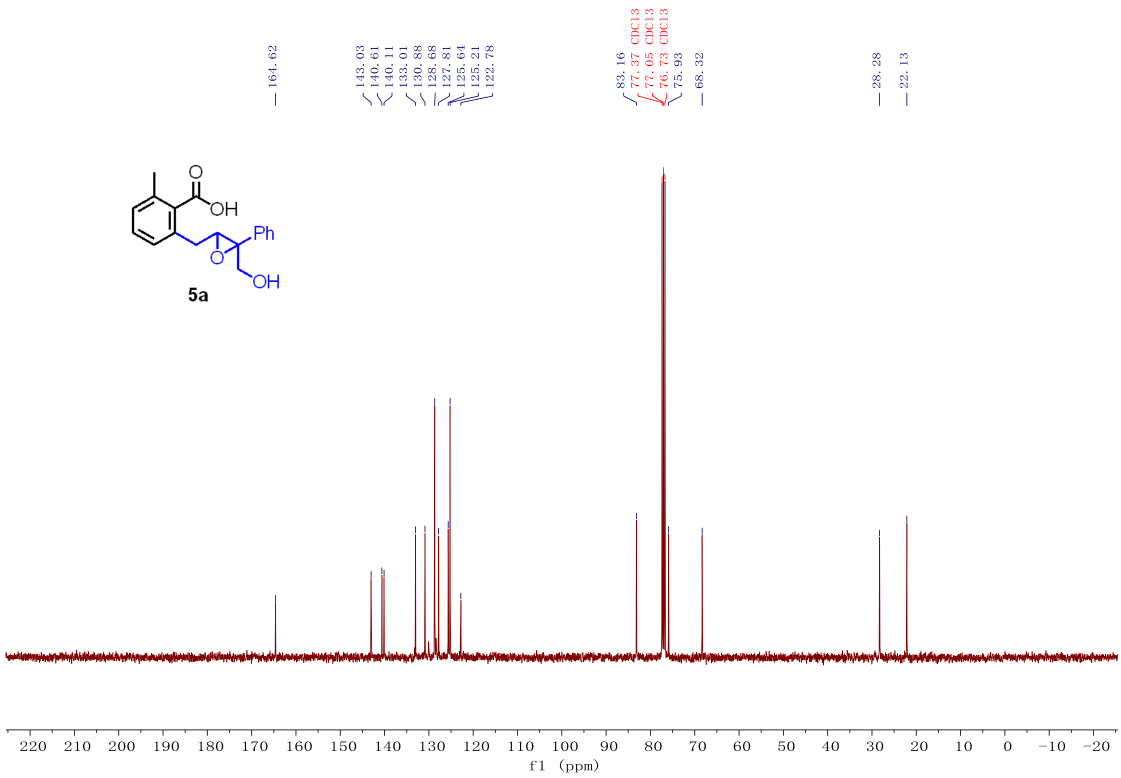

Supplementary Figure 88 ^{13}C NMR (101 MHz, $CDCl_3$) spectrum of compound **5a**

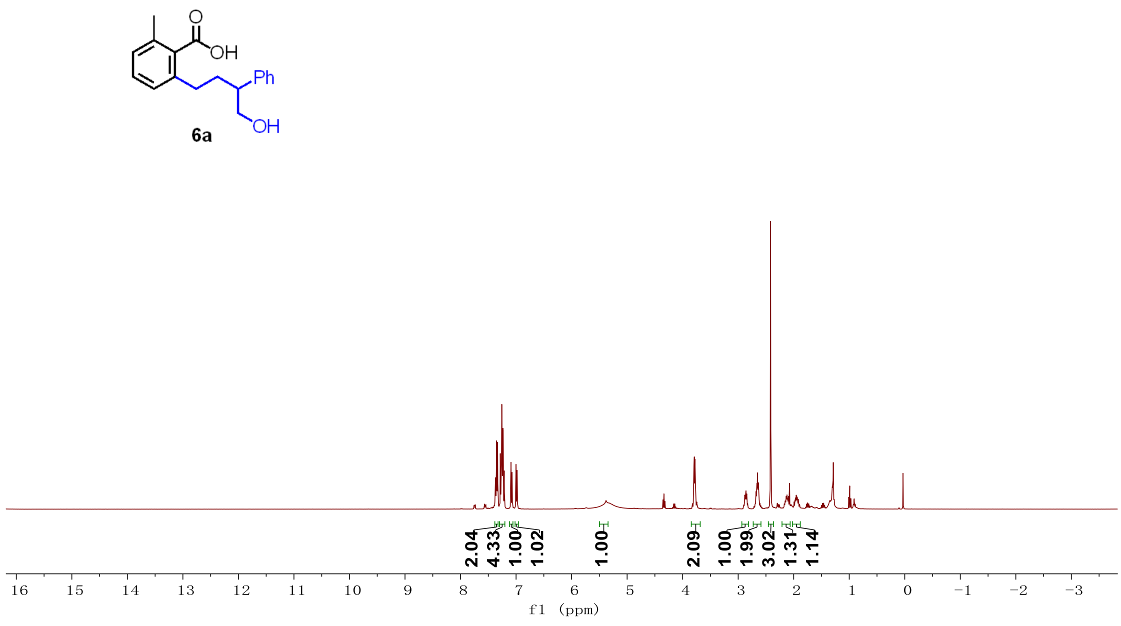

Supplementary Figure 89 ^{1}H NMR (400 MHz, $CDCl_3$) spectrum of compound **6a**

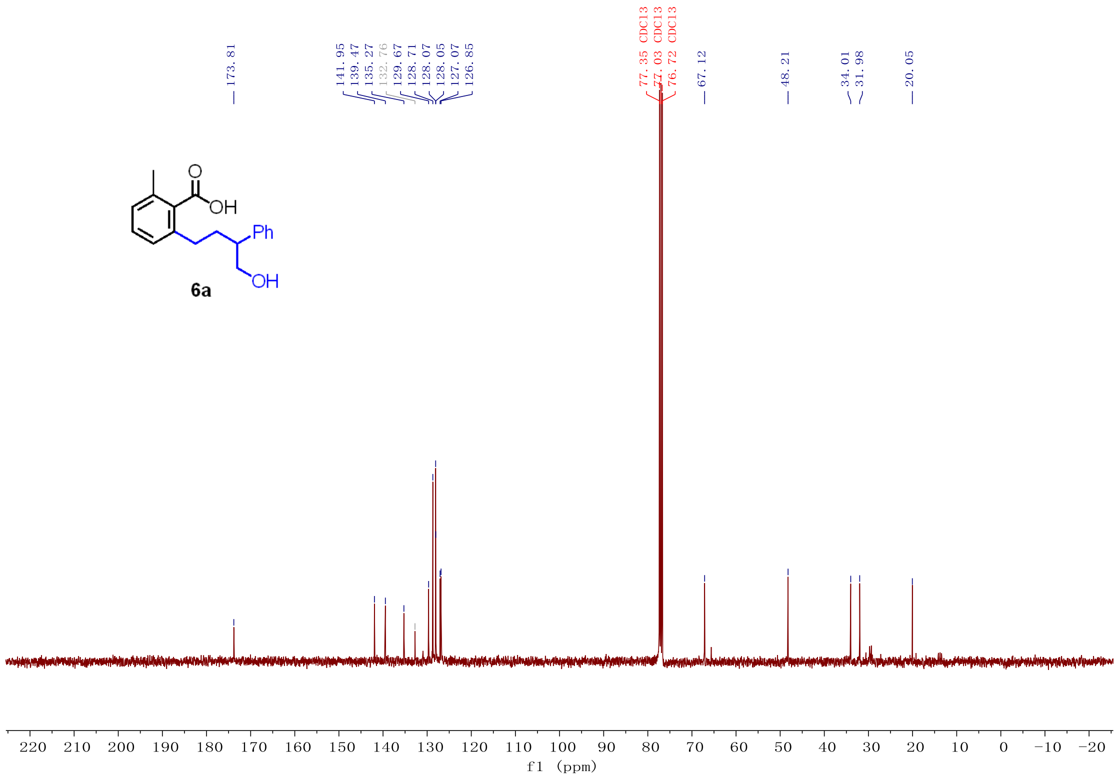

Supplementary Figure 90 ^{13}C NMR (101 MHz, $CDCl_3$) spectrum of compound **6a**

Supplementary References

[1] J. Chen, D. Liu, N. Butt, C. Li, D. Fan, Y. Liu, W. Zhang. Palladium-catalyzed asymmetric hydrogenation of α-acyloxy-1-arylethanones. *Angew. Chem. Int. Ed.* **2013**, *52*, 11632-11636.

[2] W. Guo, L. Martinez-Rodriguez, E. Martin, E. C. Escudero-Adan, A. W. Kleij. High efficient catalytic formation of (*Z*)-1,4-But-2-ene diols using water as a nucleophile. *Angew. Chem. Int. Ed.* **2016**, *55*, 11037-11040.

[3] Xu, L.; Zhu, Q.; Huang, G.; Cheng, B.; Xia, Y. Computational elucidation of the internal oxidant-controlled reaction pathways in Rh(III)-catalyzed aromatic C-H functionalization. *J. Org. Chem.* **2012**, *77*, 3017-3024.

[4] Xie, P.; Guo, W.; Chen, D.; Xia, Y. Multiple pathway for C-H cleavage in cationic Cp*Rh(III)-catalyzed C-H activation without carboxylate assistance: a computational study. *Catal. Sci. Technol.* **2018**, *8*, 4005.
