# Supplementary material for: A modular biomimetic strategy for the synthesis of macrolide P-glycoprotein inhibitors via Rh-catalyzed C-H activation
Source: Nat Commun. 2020 May 1;11:2151. doi: 10.1038/s41467-020-16084-0 (PMC7195407; doi:10.1038/s41467-020-16084-0)
Supplement: Supplementary file 3 — Description of Additional Supplementary Files [file 41467_2020_16084_MOESM3_ESM.pdf]

Description of Additional Supplementary Files

File Name: Supplementary Data 1

Description: The DFT-calculated cartesian coordinates and energies of reactant complex and CMD TS.
