# Supplementary material for: A modular biomimetic strategy for the synthesis of macrolide P-glycoprotein inhibitors via Rh-catalyzed C-H activation
Source: Nat Commun. 2020 May 1;11:2151. doi: 10.1038/s41467-020-16084-0 (PMC7195407; doi:10.1038/s41467-020-16084-0)
Supplement: Supplementary file 4 — Supplementary Data 1 [file 41467_2020_16084_MOESM4_ESM.zip › 230058_2_supp_4540435_q8gk8x (1).docx]

DFT-calculated cartesian coordinates and energies of reactant complex.

---------------------------------------------------------------------

Center Atomic Atomic Coordinates (Angstroms)

Number Number Type X Y Z

---------------------------------------------------------------------

1 6 0 -3.707357 1.882637 -2.244754

2 6 0 -2.740728 1.512263 -1.306201

3 6 0 -1.433652 1.980716 -1.428012

4 6 0 -1.079410 2.819070 -2.494960

5 6 0 -2.053541 3.180935 -3.436271

6 6 0 -3.361911 2.718892 -3.309833

7 1 0 -4.726544 1.519144 -2.147698

8 1 0 -3.006847 0.857101 -0.481479

9 1 0 -0.675905 1.692066 -0.707971

10 1 0 -1.771248 3.828061 -4.259910

11 1 0 -4.112374 3.008084 -4.040194

12 6 0 0.325169 3.326218 -2.659902

13 8 0 0.660383 3.905358 -3.712089

14 8 0 1.100701 3.117341 -1.642997

15 19 0 2.030877 4.928690 -5.506509

16 6 0 3.822786 4.833064 0.006950

17 6 0 2.916606 3.941582 0.675080

18 6 0 3.465982 2.608328 0.588065

19 6 0 4.994290 4.063762 -0.411857

20 6 0 4.780793 2.704241 -0.040607

21 6 0 3.663622 6.312821 -0.151027

22 1 0 2.611507 6.606993 -0.164438

23 1 0 4.148573 6.833458 0.685699

24 1 0 4.129863 6.667732 -1.074133

25 6 0 6.206132 4.637931 -1.077544

26 1 0 5.934392 5.387642 -1.825815

27 1 0 6.847844 5.126458 -0.332718

28 1 0 6.798567 3.866299 -1.573891

29 6 0 5.708421 1.552231 -0.261411

30 1 0 5.160152 0.643790 -0.524806

31 1 0 6.429847 1.755715 -1.055566

32 1 0 6.269775 1.348004 0.660061

33 6 0 2.855383 1.372089 1.168730

34 1 0 3.127727 0.481920 0.595628

35 1 0 3.204854 1.226458 2.199934

36 1 0 1.765242 1.442719 1.189562

37 6 0 1.593239 4.283275 1.282595

38 1 0 1.581057 3.998442 2.341213

39 1 0 1.381500 5.352324 1.216965

40 1 0 0.788189 3.745060 0.770739

41 45 0 3.170681 3.324784 -1.419978

42 6 0 3.539416 2.738956 -3.894059

43 8 0 3.623473 3.963007 -3.577537

44 8 0 3.424248 1.811150 -3.051430

45 8 0 3.532167 2.460903 -5.222784

46 1 0 3.437973 1.496408 -5.326710

---------------------------------------------------------------------

Sum of electronic and zero-point Energies= -1783.723645

Sum of electronic and thermal Energies= -1783.695038

Sum of electronic and thermal Enthalpies= -1783.694094

Sum of electronic and thermal Free Energies= -1783.785742

DFT-calculated cartesian coordinates and energies of CMD TS.

---------------------------------------------------------------------

Center Atomic Atomic Coordinates (Angstroms)

Number Number Type X Y Z

---------------------------------------------------------------------

1 6 0 -2.356872 0.525021 -3.117744

2 6 0 -0.964290 0.584894 -3.211725

3 6 0 -0.268591 1.797431 -3.027032

4 6 0 -1.044085 2.948924 -2.728960

5 6 0 -2.434097 2.903490 -2.661755

6 6 0 -3.090909 1.682334 -2.843359

7 1 0 -2.871363 -0.418747 -3.276329

8 1 0 -0.416959 -0.310028 -3.495089

9 1 0 0.549925 1.843219 -4.118938

10 1 0 -2.989124 3.813238 -2.452676

11 1 0 -4.174201 1.635690 -2.778672

12 6 0 -0.300952 4.217190 -2.451334

13 8 0 -0.726353 5.337844 -2.755369

14 8 0 0.882004 4.051366 -1.905790

15 19 0 1.804496 6.009035 -3.437280

16 6 0 3.777772 1.875613 -1.267322

17 6 0 2.813960 2.059896 -0.239722

18 6 0 1.802936 1.017216 -0.372196

19 6 0 3.400684 0.682427 -2.027250

20 6 0 2.235118 0.119356 -1.429623

21 6 0 4.994780 2.705142 -1.540686

22 1 0 4.954796 3.666568 -1.022898

23 1 0 5.897871 2.182514 -1.199345

24 1 0 5.110966 2.897583 -2.611775

25 6 0 4.196047 0.107206 -3.158934

26 1 0 4.606384 0.895208 -3.796790

27 1 0 5.041138 -0.475995 -2.769259

28 1 0 3.593138 -0.556756 -3.783206

29 6 0 1.622492 -1.205382 -1.764293

30 1 0 0.565475 -1.246542 -1.495347

31 1 0 1.720368 -1.448753 -2.825781

32 1 0 2.139282 -1.992473 -1.198575

33 6 0 0.672086 0.780652 0.582370

34 1 0 -0.158236 0.258628 0.100639

35 1 0 1.011338 0.166586 1.427667

36 1 0 0.288776 1.720645 0.988021

37 6 0 2.772583 3.148246 0.787303

38 1 0 2.972017 2.731666 1.782692

39 1 0 3.519323 3.921443 0.593640

40 1 0 1.788426 3.625929 0.815061

41 45 0 1.748131 2.160472 -2.202856

42 6 0 1.999970 2.834837 -5.151583

43 8 0 2.413645 3.309452 -4.056567

44 8 0 1.112704 1.932848 -5.277258

45 8 0 2.533502 3.359837 -6.266695

46 1 0 2.109611 2.930900 -7.032527

---------------------------------------------------------------------

Sum of electronic and zero-point Energies= -1783.679452

Sum of electronic and thermal Energies= -1783.651912

Sum of electronic and thermal Enthalpies= -1783.650968

Sum of electronic and thermal Free Energies= -1783.737198

DFT-calculated cartesian coordinates and energies of CMD TS.
